# Ureidopeptide GLP-1 analogues with prolonged activity *in vivo via* signal bias and altered receptor trafficking†
†Electronic supplementary information (ESI) available. See DOI: 10.1039/c9sc02079a


**DOI:** 10.1039/c9sc02079a

**Published:** 2019-09-11

**Authors:** Juliette Fremaux, Claire Venin, Laura Mauran, Robert Zimmer, Florian Koensgen, Didier Rognan, Stavroula Bitsi, Maria A. Lucey, Ben Jones, Alejandra Tomas, Gilles Guichard, Sébastien R. Goudreau

**Affiliations:** a UREkA – ImmuPharma Group , 2 rue Robert Escarpit , 33607 Pessac , France . Email: sebastien.goudreau@immupharma.com; b Univ. Bordeaux , CNRS , CBMN , UMR 5248 , Institut Européen de Chimie et Biologie , 2 rue Robert Escarpit , 33607 Pessac , France . Email: g.guichard@iecb.u-bordeaux.fr; c Laboratoire d'Innovation Thérapeutique , UMR7200 CNRS-Université de Strasbourg , 74 route du Rhin , 67400 Illkirch , France; d Section of Cell Biology and Functional Genomics , Imperial College London , London W12 0NN , UK; e Section of Investigative Medicine , Imperial College London , London W12 0NN , UK

## Abstract

The high demand of the pharmaceutical industry for new modalities to address the diversification of biological targets with large surfaces of interaction led us to investigate the replacement of α-amino acid residues with ureido units at selected positions in peptides to improve potency and generate effective incretin mimics. Based on molecular dynamics simulations, N-terminally modified GLP-1 analogues with a ureido residue replacement at position 2 were synthesized and showed preservation of agonist activity while exhibiting a substantial increase in stability. This enabling platform was applied to exenatide and lixisenatide analogues to generate two new ureidopeptides with antidiabetic properties and longer duration of action. Further analyses demonstrated that the improvement was due mainly to differences in signal bias and trafficking of the GLP-1 receptor. This study demonstrates the efficacy of single α-amino acid substitution with ureido residues to design long lasting peptides.

## Abstract 2

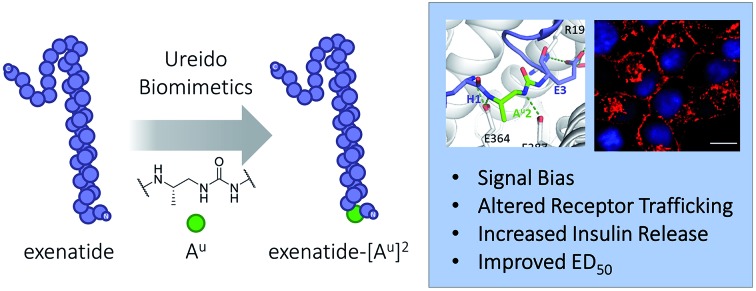
This study demonstrates the efficacy of single α-amino acids substitution with ureido residues to design long lasting peptides.

## Introduction

Backbone modifications are a powerful strategy to improve peptide properties as they generally ameliorate their protection against proteolysis and therefore their duration of action.^1^,^2^ Such an approach is far from trivial as modifying the backbone of peptides has generally drastic negative impacts on binding properties, and consequently, potency. Noteworthily aza-amino acids^3^,^4^ and β-amino acids^5^–^7^ have proved to be efficient α-amino acid substitutions. Despite the potential of these approaches, new enabling platforms based on backbone modifications are still needed to meet the increasingly demanding requirements of the pharmaceutical industry in peptide mimicry.^8^

**Fig. 1:**
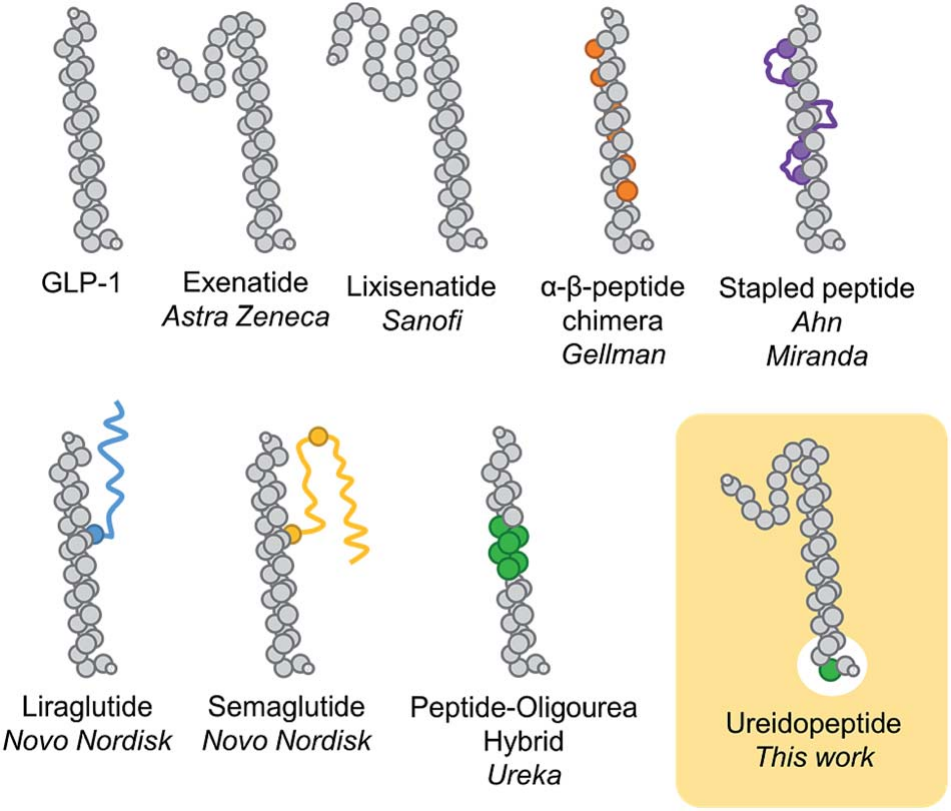
Schematic representation of different GLP-1 analogues previously reported and the present approach (ureidopeptide). GLP-1 analogue modifications are highlighted in colour; orange: β-amino acids, purple: macrocycles, blue and yellow: lipidation, green: ureido residue.

In the past decade, the incretin glucagon-like peptide-1 (GLP-1) has been largely studied because of its blood glucose control properties which have led to multiple new treatments for type 2 diabetes mellitus (T2DM).^9^ This pharmacological target remains of considerable ongoing interest as several studies indicate that GLP-1 analogues could be used for other important indications such as obesity, major adverse cardiac events, Alzheimer's disease, or non-alcoholic steatohepatitis (NASH).^9^ One particular problem with the use of native GLP-1, a 29 amino acid natural peptide hormone, is its short *in vivo* half-life of 2–3 min.^10^ Many modifications have therefore been developed to prolong the GLP-1 lasting period, notably, sequence remodelling and extension (exenatide and lixisenatide),^11^ fatty acid acylation to promote binding to plasma albumin (liraglutide and semaglutide),^11^ bonding or fusion to large proteins (dulaglutide, albiglutide, and efpeglenatide),^11^ non-natural amino acid replacements,^12^ side chain cross linking,^13^ and more recently α → β-residue replacements,^14^ thioamide,^15^ and peptide–oligourea hybrids^16^ (Fig. 1). A common modification of GLP-1 analogues is the replacement of Ala2 with another amino acid (*i.e.* Gly) to prevent proteolytic cleavage by DPP-4.^10^,^11^ However, despite having a glycine in position 2, exenatide, an FDA approved once-weekly treatment for T2DM, is still susceptible to degradation at its N-terminus.^17^ We therefore hypothesize that modifying the backbone of exenatide might improve its proteolytic stability and prolong its activity *in vivo*. However, backbone modifications at the N-terminal part of incretins and other ligands of class B GPCRs have scarcely been reported^15^,^18^–^20^ which reflects the difficulty to mimic the complex network of interactions in the binding pocket of the receptor transmembrane domain.^21^

Herein we report the utilization of a ureido residue replacement at position 2 of GLP-1 analogues to improve their pharmacodynamic properties *via* selective enhancement of G protein-dependent cAMP signalling and altered GLP-1R trafficking.

## Results and discussion

### Peptide design, synthesis and functional assays

Although oligourea foldamers^16^,^22^–^25^ have recently been reported to be effective α-helix mimics, their constitutive units – ureido residues – have scarcely been studied in the context of single α-amino acid replacements.^26^ Therefore, the ability of GLP-1 analogues with a ureido unit (X^u^) (Table 1) at position 2 (*e.g.* GLP-1[A^u^]^2^ (**3**)) to interact with the GLP-1 receptor (GLP-1R) was first tested *in silico* by molecular dynamics simulation (see the ESI†). The cryo-EM structure of GLP-1R (PDB ID: ; 5VAI),^21^ which contains GLP-1 as the ligand, was used both as a starting point and as a comparator to evaluate the binding mode of GLP-1[A^u^]^2^ (**3**).

**Table 1:** Bioactivity in cAMP production functional assays using HEK293 cells transfected with GLP-1R and the mouse plasma half-life of GLP-1, exenatide, lixisenatide and cognate monosubstituted analogues

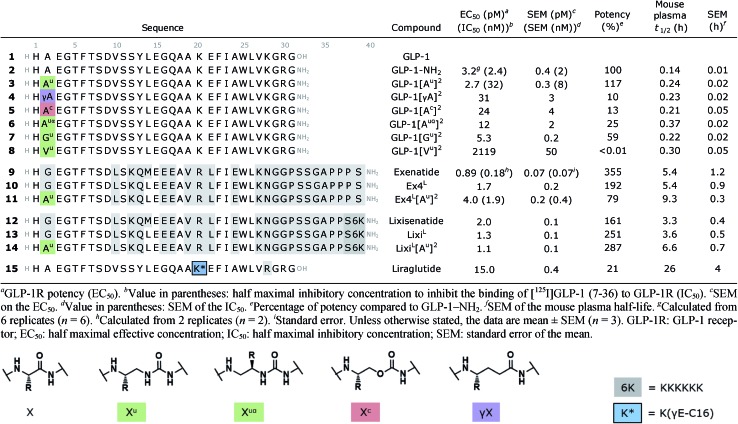

**Fig. 2:**
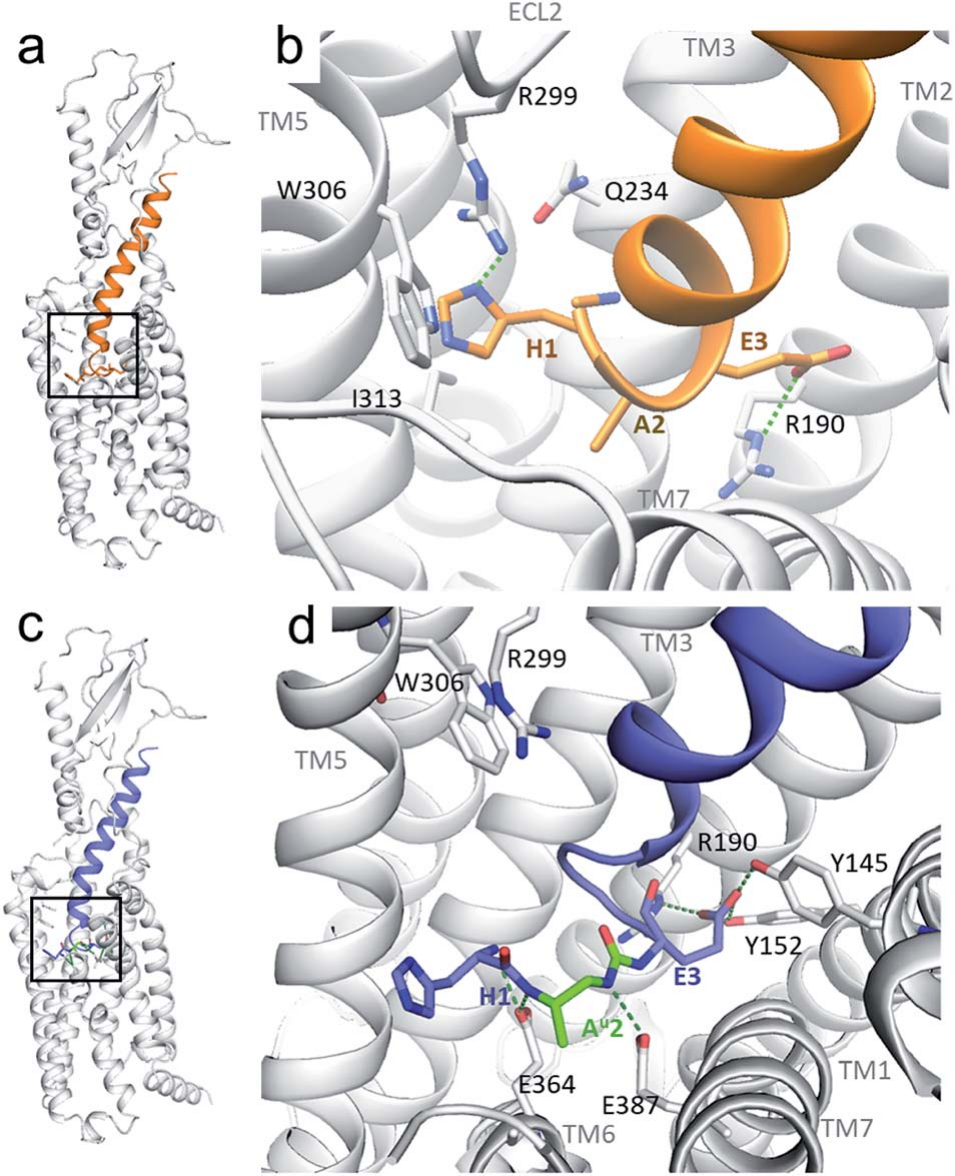
Predicted structure of the GLP-1R/GLP-1[A^u^]^2^ complex compared to the cryo-EM structure of the GLP-1R/GLP-1 complex. (a and b) GLP-1 (in orange) in the GLP-1R TMD and an enlarged image (b) of the N-terminal part (extracted from the cryo-EM structure of the GLP-1R/GLP-1 complex (PDB ; 5VAI));^21^ (c and d) predicted structure of GLP-1[A^u^]^2^ (**3**) (in blue with Ala^u^2 in green) in the GLP-1R TMD and an enlarged image (d) of the N-terminal part.

As seen from the predicted model, the ureido peptide **3** sits deeper in the cavity of the transmembrane domain (TMD) compared to native GLP-1, and its N-terminus is engaged in new H-bond interactions with the receptor (Fig. 2). In particular, the terminal amino group of His1 and main chain NHs of the Ala^u^2 unit are H-bonded to Glu364 and Glu387 located in transmembrane helices TM6 and TM7, respectively. Moreover, the salt bridge between Glu3 and Arg190 is supplemented by two new H-bonds to Tyr145 and Tyr152. On the basis of these results, we synthesized compound **3** to further investigate to what extent agonist activity is retained upon Ala → Ala^u^ replacement at position 2. The synthesis of GLP-1[A^u^]^2^ (**3**) was readily achieved using standard solid phase synthesis with *N*-Fmoc protected amino acids and succinimidyl-[(2*S*)-2-azidopropyl]carbamate for ureido insertion.^27^ The agonistic activity of the oligomer was determined *in vitro* using HEK293 cells transfected with GLP-1R (Table 1) and βTC6 cells which express GLP-1R endogenously (see ESI, Table S1†) by measuring the receptor-mediated cAMP production in the presence of the agonist. The results show that **3** is active with an EC_50_ of 2.7 pM which is equivalent to that of GLP-1–NH_2_ (**2**) (3.2 pM). An agonist radioligand assay was then used to further evaluate the interaction of **3** with GLP-1R (Table 1). The data show that **3** binds GLP-1 in the nM range (IC_50_ = 32 nM) but less tightly than **2** (2.4 nM). It is noteworthy that despite a 10-fold reduction in binding efficacy, the agonist activity (EC_50_) of **3** is largely retained.

To validate our hypothesis that the additional NH of urea is involved in the binding, we synthesized two new analogues in which the urea NH in **3** was replaced by either a CH_2_ group or an oxygen (O) to generate γ-amino-acid- and carbamate-containing peptides GLP-1[γA]^2^ (**4**) and GLP-1[A^c^]^2^ (**5**). These modifications cause a 5- to 10-fold loss in potency relative to **3**. Attempts to vary the nature of the side chain of the ureido unit in **3** (*e.g.* Val^u^, Ile^u^, Phe^u^, Glu^u^, Ser^u^, *etc.*) led to analogues with significantly reduced biological activity (see the ESI, Table S1†). These results are consistent with those of the previously reported SAR studies^28^ and with the model presented in Fig. 2b which shows the limited space available in the pocket around the ureido residue to accommodate larger side chains. Exchanging the methyl side chain for an isopropyl (Val^u^, **8**), removing the methyl side chain (Gly^u^, **7**) and shifting the methyl group of the ureido residue from the β- to the α-carbon (Ala^uα^, **6**) were also tried, but again with a loss of potency (>1000-, 5- and 24-fold respectively), demonstrating that the methyl side chain on the β-carbon is optimal to stabilize GLP-1R in the fully active state.

We then investigated the scope of this approach by incorporating the Ala^u^2 modification in exenatide (**9**) and lixisenatide (**12**), two GLP-1 analogues currently approved by the FDA for the treatment of T2DM. More precisely, Ala^u^ was introduced in the Leu14 analogues Ex4^L^ (**10**) and Lixi^L^ (**13**), which are less susceptible to oxidation and show similar potency.^29^ As before, the resulting monosubstituted ureido analogues **11** and **14** displayed potent agonist activities, comparable to that of the native peptide in both cases. Binding studies were also performed on exenatide (**9**) and the ureidopeptide analogue **11** and again a ten-fold difference in binding (IC_50_ of 0.18 and 1.9 nM, respectively) was observed, consistent with the results obtained for **3** (Table 1).

### Mouse plasma and *in vivo* studies in mice

**Fig. 3:**
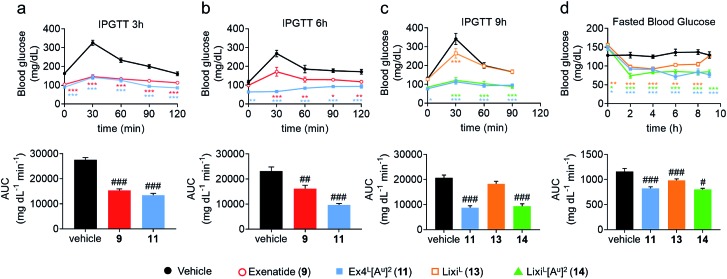
Pharmacodynamic studies in healthy mice (C57BL/6J, male, 20–25 g). Dosage: 1 μg per mouse (10 nmol kg^–1^) i.v. Formulation: 4 μg mL^–1^ in PBS 1×. IPGTT: glucose 2 g kg^–1^ i.p. at T0. (a) IPGTT 3 h after dosing: trace and AUC. Fasted 6 h. (b) IPGTT 6 h after dosing: trace and AUC. Fasted 6 h. (c) IPGTT 9 h after dosing: trace and AUC. Fasted 9 h. (d) Fasted blood glucose before and after dosing and before the IPGTT 9 h in (c). Data are mean ± SEM (*n* = 6). Statistics by two-way ANOVA and Bonferroni post-test: *p < 0.05, **p < 0.01, ***p < 0.001, comparing the vehicle to oligomers; one way ANOVA with Dunnett's multiple comparison test: ^#^*p* < 0.05, ^##^*p* < 0.01, ^###^*p* < 0.001, comparing the vehicle to oligomers. IPGTT: intraperitoneal glucose tolerance test; AUC: area under the curve; i.v.: intra venous; i.p.: intra peritoneal.

To further evaluate the potential benefits of the Ala2 → Ala^u^2 replacement in GLP-1R activating peptides, a comparative stability study in mouse plasma was conducted on compounds **2–14**. Solutions of peptides, ureidopeptides or oligomers were treated with mouse plasma and their stabilities were assessed. Remarkably, in all cases, the introduction of the ureido residue in position 2 resulted in a substantially longer *in vitro* half-life. Ex4^L^[A^u^]^2^ (**11**) was found to persist for significantly longer in the serum (half-life of 9.3 hours) than Ex4^L^ (**10**) for which a half-life of 5.4 hours was measured. So by replacing only one amino acid with a ureido alanine (Ala^u^) we increased the half-life in mouse plasma by 1.7-fold with no impact on the EC_50_. For the next step, we conducted a series of experiments in mice to see if this improvement could be translated *in vivo*. Healthy mice were fasted for 6 hours and acutely treated with 1 μg (10 nmol kg^–1^) of GLP-1 analogues injected i.v. 3, 6, and 9 hours before performing an intra-peritoneal glucose tolerance test (IPGTT) (Fig. 3).

**Fig. 4:**
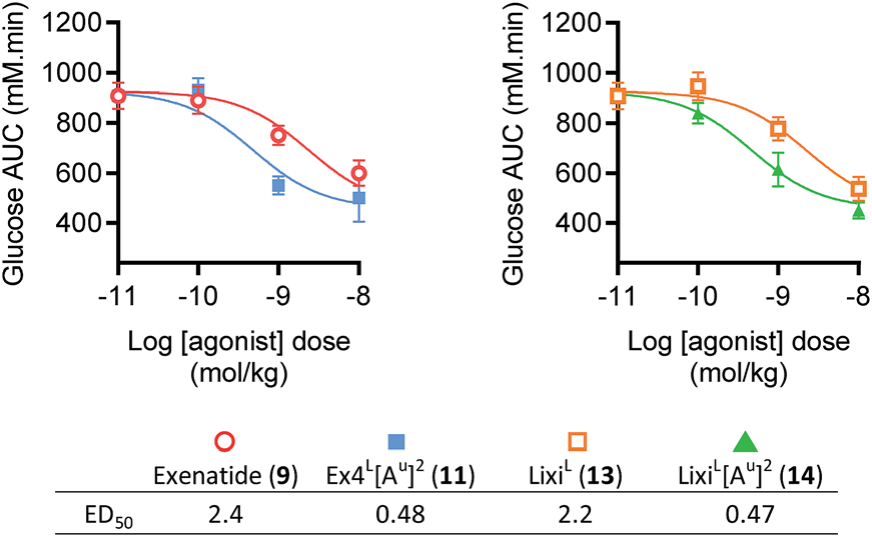
Dose–response relationships of exenatide (**9**), Lixi^L^ (**13**) and their putative ureidopeptides Ex4^L^[A^u^]^2^ (**11**) and Lixi^L^[A^u^]^2^ (**14**) in healthy mice (C57BL/6J, male, 25–30 g). Dose response relationships from IPGTT results shown in the ESI, Fig. S4†, 3-parameter fits of area-under-curve values (*n* = 8). Dosage and formulation: 0.01 to 10 nmol kg^–1^ in 100 μL saline i.p. Fasted 4 h prior to agonist administration. IPGTT (2 mmol kg^–1^ glucose) performed 6 hours after administration of indicated agonist dose in lean C57Bl/6 mice. Data are mean ± SEM.

When the IPGTT is performed 3 hours after the administration of the GLP-1 analogues or the vehicle, we observe a strong control over blood glucose for both exenatide (**9**) and Ex4^L^[A^u^]^2^ (**11**) compared to the vehicle, with a significant decrease even at T0 before the administration of glucose (Fig. 3a). Interestingly, after 6 hours, we can already observe that exenatide has partially lost its efficacy while **11** is still fully active (Fig. 3b). In the experiment shown in Fig. 3c, glucose was administered 9 hours after injection of ureidopeptides **11** and **14** which were directly compared to the lixisenatide analogue Lixi^L^ (**13**) as a reference. Lixisenatide is a once daily GLP-1R agonist with an increased binding affinity to the receptor and a prolonged half-life *in vivo* compared to exenatide.^11^ In this case, Lixi^L^ (**13**) shows a small glucose-lowering effect 9 hours after treatment, compared to the vehicle. In contrast, **11** and **14** demonstrated a strong ability to control blood glucose levels with almost no increase in the IPGTT curves. These results are in accordance with the fasting blood glucose curves covering the 9 hours preceding the IPGTT where we can see that the effect of the ureidopeptides **11** and **14** is maintained while in the case of **13** the blood glucose tends to increase after 4 hours (Fig. 3d). In order to quantify the improvements of the ureidopeptides *in vivo*, a dose–response relationship study was conducted to determine the ED_50_ of **9**, **11**, **13** and **14** (Fig. 4). Mice were treated with different doses of analogues and IPGTTs were performed after 6 h. From these results dose–response curves were obtained and *in vivo* ED_50_ values were calculated. Exenatide (**9**) and Lixi^L^ (**13**) showed ED_50_ values of 2.4 and 2.2 nM, respectively, while the ureidopeptide analogues Ex4^L^[A^u^]^2^ (**11**) and Lixi^L^[A^u^]^2^ (**14**) demonstrated a five-fold improvement with ED_50_ values of 0.48 and 0.46 nM, respectively.

These studies clearly show that introducing a ureido alanine at position 2 of a GLP-1 analogue can significantly prolong its efficacy *in vivo*.

**Fig. 5:**
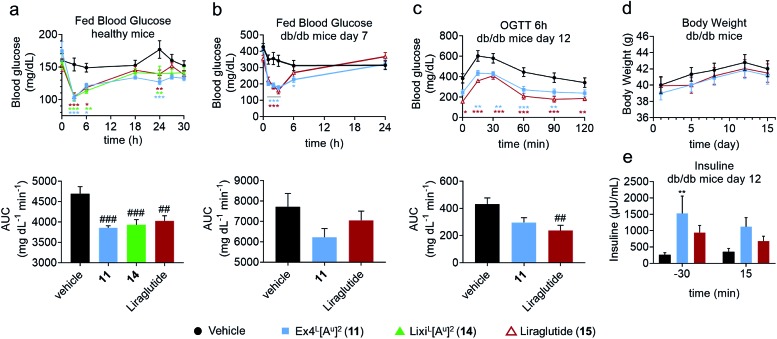
Comparative pharmacodynamics of Ex4^L^[A^u^]^2^ (**11**), Lixi^L^[A^u^]^2^ (**14**) and liraglutide (**15**) in mice. (a) Fed blood glucose in healthy mice (C57BL/6J, male, 20–25 g): trace and AUC (*n* = 6). Dosage: 1 μg per mouse (10 nmol kg^–1^) i.v. Formulation: 4 μg mL^–1^ in PBS 1×. (b–e) Study on db/db mice treated over 15 days (*n* = 10). Dosage: 100 μg kg^–1^ (25 nmol kg^–1^) s.c. once a day (*n* = 10). Formulation: 20 μg mL^–1^ in PBS 1×. (b) Fed blood glucose before and after treatment on day 7: trace and AUC. (c) OGTT 6 hours after dosing on day 12: trace and AUC. OGTT: glucose 1 g kg^–1^ i.p. at T0. (d) Body weight across the study. (e) Plasma insulin before and after the OGTT on day 12. Data are mean ± SEM. Statistics by two-way ANOVA and Bonferroni post-test: *p < 0.05, **p < 0.01, ***p < 0.001, comparing the vehicle to oligomers; one way ANOVA with Dunnett's multiple comparison test: ^#^*p* < 0.05, ^##^*p* < 0.01, ^###^*p* < 0.001, comparing the vehicle to oligomers. IPGTT: intraperitoneal glucose tolerance test; AUC: area under the curve; i.v.: intra venous; s.c.: subcutaneous; i.p.: intra peritoneal.

In another study in healthy mice, liraglutide (**15**) and ureidopeptides **11** and **14** were administered with the same dose as in the IPGTTs and their fed blood glucose was followed over 30 hours to assess their maximum duration of action (Fig. 5a). Interestingly, all three compounds had similar fed blood glucose curves which suggested that **11** and **14** have a similar lasting period to liraglutide. Liraglutide is a long acting FDA-approved GLP-1 analogue with a fatty acid chain attached on Lys26 to increase albumin binding and is used as a once daily treatment (Table 1).^11^ To further validate the potential relevance of ureido analogues of GLP-1 for treating T2DM, we next conducted a study on diabetic db/db mice. The mice were treated *via* the subcutaneous route, once daily over 15 days with **11** (25 nmol kg^–1^) or with liraglutide (25 nmol kg^–1^). A number of parameters were measured during the experiment such as body weight, plasma insulin, fed blood glucose, and glycated haemoglobin (HbA1c) levels (Fig. 5 and the ESI†). Fed blood glucose showed similar reductions as in healthy mice, confirming the results while suggesting that **11** might be even better than liraglutide (Fig. 5b). However liraglutide tends to have a better control on the fasted blood glucose as demonstrated by the blood glucose at T0 in the IPGTT after 6 hours of administration of the GLP-1 analogues, which is reflected in a better AUC (Fig. 5c). Interestingly, treatment with **11** led to a significant increase in insulin production compared to that with the vehicle and is superior to liraglutide (Fig. 5e).

Overall, the data obtained in this study showed that **11** and liraglutide have similar activities in the db/db mouse model. This is remarkable considering that liraglutide's longer lasting period is due to it binding to serum albumin through its lipid side chain, while it is unlikely that Ex4^L^[A^u^]^2^**11** binds to albumin to the same extent.

### Pharmacokinetic studies

In light of these interesting results, we sought to understand the underlying causes of such improvements of exenatide and lixisenatide by the substitution of one amino acid over 39 and 45, respectively. Our hypothesis was that the cleavage between positions 1 and 2 of exenatide (**9**) and lixisenatide (**12**) was a determining factor for their *in vivo* half-life^17^ and, as we managed to improve the proteolytic stability of the peptides *in vitro*, as demonstrated by the increased mouse plasma stability, this would be reflected in improved *in vivo* half-life profiles. To validate this hypothesis, we conducted pharmacokinetic studies on healthy mice with Ex4^L^ (**10**) and Ex4^L^[Ala^u^]^2^ (**11**). Surprisingly, no significant difference was observed between the two analogues (ESI†).

### GLP-1R biased signalling and trafficking

**Fig. 6:**
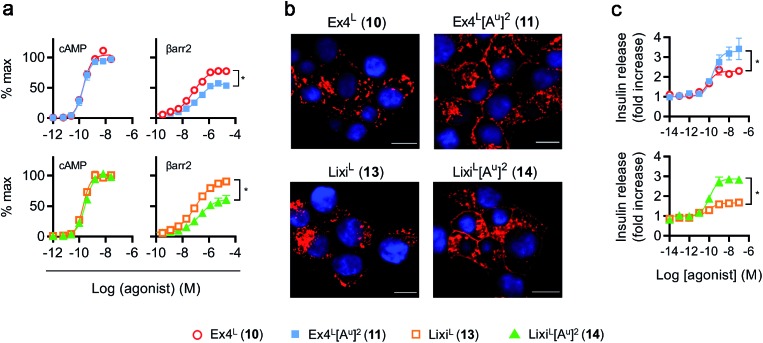
GLP-1R biased signalling and trafficking studies. (a) Cyclic AMP (cAMP) and β-arrestin-2 (βarr2) responses in PathHunter CHO–GLP-1R cells, 30 min stimulation, all ligands and pathways run in parallel, results normalized to global maximum responses (cAMP) or GLP-1–NH_2_ (**2**) maximal response (βarr2), 4-parameter fit shown, *n* = 5 independent experiments. (b) Confocal analysis of SNAP-GLP-1R internalization in SNAP-GLP-1R-expressing INS-1 832/3 cells labeled with SNAP-Surface 549 probe (red) for 30 min and then stimulated with 10 nM of the indicated ligand for a further 30 min. Nuclei (DAPI), blue; size bars, 10 μm. (c) Insulin secretion in INS-1 832/3 cells, 16 h stimulation at 11 mM glucose, all ligands run in parallel, normalised to basal response in each assay, 3-parameter fit shown, *n* = 5 independent experiments, *E*_max_ compared by 2-way repeat measures ANOVA with Tukey's test.

**Fig. 7:**
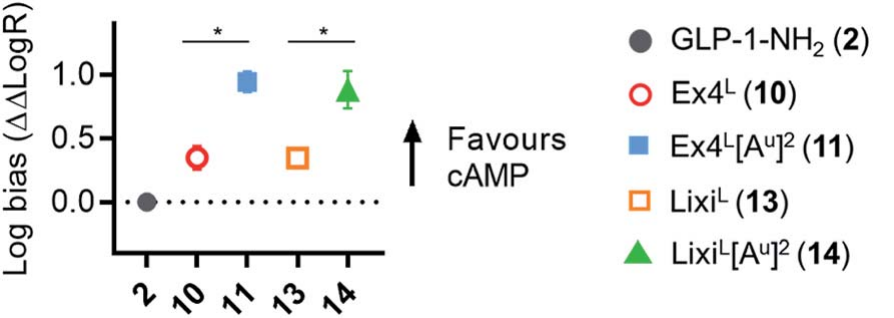
GLP-1R biased signalling. Signal bias calculated from the data shown in Fig. 6a, expressed relative to GLP-1–NH_2_ (**2**), determined using a modified form of the operational model (see Methods for details†), and 1-way randomised block ANOVA with Tukey's test of difference between Log *R* values for cAMP *vs.* β-arrestin-2 responses for each ligand.

As it was difficult to explain the full extent of the pharmacodynamic effects solely from the *in vitro* plasma stability, we sought other hypotheses that would help to fully account for our *in vivo* results. It has been reported in recent studies that modifying GLP-1 and GLP-1-related peptides can lead to the selective enhancement of particular intracellular signalling pathways, usually referred to as “signal bias”.^14^,^30^,^31^ In one example, exenatide-derived peptides with amino acid substitutions close to the N-terminus (such as “exenatide-F1”) showed reduced recruitment of β-arrestin whilst maintaining full agonist behaviour for G protein-dependent cAMP signalling;^30^ furthermore, these compounds displayed markedly reduced tendencies to induce GLP-1R endocytosis. These properties allowed for enhancement of insulin secretion *via* a combination of reduced GLP-1R desensitisation and preservation of surface GLP-1Rs available to the extracellular agonist. We hypothesized that a similar phenomenon may apply to the ureidopeptides described herein which also contain modifications at the N-terminal portion of exenatide. Therefore, signal bias and effects on GLP-1R endocytosis were assessed for Ex4^L^ (**10**), Lixi^L^ (**13**) and their ureidopeptide analogues **11** and **14**, respectively (Fig. 6). Interestingly, in both cases, the recruitment of β-arrestin to GLP-1Rs was selectively diminished, as shown in Fig. 6a, with quantification of bias *via* the operational model^32^ confirming a strong preference for cAMP signalling (Fig. 7). Moreover, endocytosis of the GLP-1R was noticeably reduced after treatment with ureido- compared to non-ureido-peptides, as assessed by confocal microscopy in INS-1 832/3 beta cells with endogenous GLP-1R knocked out by CRISPR/Cas9,^33^ modified to express SNAP-tagged human GLP-1R which was surface-labelled prior to agonist treatment (Fig. 6b and the ESI, Fig. S7†). The reduced residence time of **11** suggested by its increased IC_50_, in conjunction with this reduced internalisation propensity, is compatible with the previously described relationship between agonist binding kinetics and GLP-1R endocytosis.^30^ In keeping with the cellular mechanism described by Jones *et al.*,^30^ both exenatide and lixisenatide ureidopeptide analogues **11** and **14** showed significantly increased insulin release (Fig. 6c) assessed *in vitro*, mirroring our previous observations in the db/db mouse study (Fig. 5e). The combination of reduced β-arrestin recruitment and GLP-1R endocytosis, associated with enhanced insulin release *in vitro* and *in vivo*, is remarkably consistent with the characteristics of exenatide-F1,^30^ suggesting a similar mechanism of action. Moreover, we note similarities with another biased GLP-1R agonist, exendin-P5,^31^ which shows selectively increased coupling to cAMP signalling in spite of somewhat reduced binding affinity *versus* exenatide.^34^ Further investigations are required to fully understand the mechanism of action of these analogues and will be reported in due course.

## Conclusions

In summary, we rationalized the design of singly substituted ureido analogues of GLP-1 using molecular modelling, showing that the replacement of the amino acid in position 2 of GLP-1 with an ureido alanine (Ala^u^) does not impair its agonist activity. Moreover, the same modification introduced in exenatide and lixisenatide considerably prolongs their efficacy *in vivo* with a significant reduction of blood glucose levels observed over at least 9 hours in healthy mice. Additionally, we have shown that this monosubstituted analogue is also active in db/db mice, a type 2 diabetes mellitus animal model, giving similar result to the FDA approved liraglutide. A key mechanism for the improved biological efficacy of these compounds appears to be the enhancement of insulin secretion *via* effects on signal bias and GLP-1R trafficking on pancreatic β-cells. Indeed, introducing a ureido residue at position 2 of exenatide and lixisenatide preserves their efficacy while diminishing their affinity, which may result in a shorter residence time in the receptor and therefore reduced endocytosis of the GLP-1 receptor. Further studies are required to fully elucidate this mechanism.

All in all, this study demonstrates and validates the approach of substituting simple α-amino acids with their ureido counterparts to improve the pharmacological properties of biologically relevant peptides. The evaluation of replacing multiple amino acids to generate ureidopeptides with multiple isolated ureido residues and/or the combination of this approach with other strategies like acylation with fatty acids or combination with oligoureas to generate ureidopeptide–oligourea hybrids will be reported in due course.

## Author contributions

J. F., C.·V., R. H. Z., G. G. and S. R. G. designed the analogues. J. F. and C. V. synthesized the analogues. L. M. performed the mouse plasma degradation assays and the functional assays on the HEK293 cells. F. K. and D. R. performed the molecular dynamics simulations. M. A. L. and B. J. performed the dose–response study. S. B., B. J. and A. T. performed the biased signalling and trafficking studies. G. G. and S. R. G conceived the project. S. R. G. supervised the project. J. F., G. G. and S. R. G. analyzed the data and wrote the manuscript.

## Conflicts of interest

The authors declare the following competing financial interests. J. F., C.·V., L. M., R. H. Z., G. G., and S. R. G. are inventors on a patent application covering the GLP-1 analogues described here. J. F., C.·V., L. M., R. H. Z. and S. R. G. work for UREKA Sarl, which is pursuing biomedical applications of ureidopeptides.

## Supplementary Material

Supplementary information

## References

[cit1] Erak M., Bellmann-Sickert K., Els-Heindl S., Beck-Sickinger A. G. (2018). Bioorg. Med. Chem..

[cit2] Valeur E., Guéret S. M., Adihou H., Gopalakrishnan R., Lemurell M., Waldmann H., Grossmann T. N., Plowright A. T. (2017). Angew. Chem., Int. Ed..

[cit3] Chingle R., Proulx C., Lubell W. D. (2017). Acc. Chem. Res..

[cit4] Zhang Y., Malamakal R. M., Chenoweth D. M. (2015). J. Am. Chem. Soc..

[cit5] Johnson L. M. and Gellman S. H., in Methods in Enzymology, Elsevier, 2013, vol. 523, pp. 407–429. 10.1016/B978-0-12-394292-0.00019-9 PMC3928965 23422441

[cit6] Seebach D., Dubost E., Mathad R. I., Jaun B., Limbach M., Löweneck M., Flögel O., Gardiner J., Capone S., Beck A. K., Widmer H., Langenegger D., Monna D., Hoyer D. (2008). Helv. Chim. Acta.

[cit7] Koglin N., Zorn C., Beumer R., Cabrele C., Bubert C., Sewald N., Reiser O., Beck-Sickinger A. G. (2003). Angew. Chem., Int. Ed..

[cit8] Mimmi S., Maisano D., Quinto I., Iaccino E. (2019). Trends Pharmacol. Sci..

[cit9] Drucker D. J. (2018). Cell Metab..

[cit10] Jessen L., Aulinger B. A., Hassel J. L., Roy K. J., Smith E. P., Greer T. M., Woods S. C., Seeley R. J., D'Alessio D. A. (2012). Endocrinology.

[cit11] Cheang J. Y., Moyle P. M. (2018). ChemMedChem.

[cit12] Meng H., Krishnaji S. T., Beinborn M., Kumar K. (2008). J. Med. Chem..

[cit13] Murage E. N., Gao G., Bisello A., Ahn J.-M. (2010). J. Med. Chem..

[cit14] Hager M. V., Johnson L. M., Wootten D., Sexton P. M., Gellman S. H. (2016). J. Am. Chem. Soc..

[cit15] Chen X., Mietlicki-Baase E. G., Barrett T. M., McGrath L. E., Koch-Laskowski K., Ferrie J. J., Hayes M. R., Petersson E. J. (2017). J. Am. Chem. Soc..

[cit16] Fremaux J., Venin C., Mauran L., Zimmer R. H., Guichard G., Goudreau S. R. (2019). Nat. Commun..

[cit17] Liao S., Liang Y., Zhang Z., Li J., Wang J., Wang X., Dou G., Zhang Z., Liu K. (2015). PLoS One.

[cit18] Bai X., Niu Y., Zhu J., Yang A.-Q., Wu Y.-F., Ye X.-S. (2016). Bioorg. Med. Chem..

[cit19] Liu S., Cheloha R. W., Watanabe T., Gardella T. J., Gellman S. H. (2018). Proc. Natl. Acad. Sci. U. S. A..

[cit20] Cheloha R. W., Chen B., Kumar N. N., Watanabe T., Thorne R. G., Li L., Gardella T. J., Gellman S. H. (2017). J. Med. Chem..

[cit21] Zhang Y., Sun B., Feng D., Hu H., Chu M., Qu Q., Tarrasch J. T., Li S., Sun Kobilka T., Kobilka B. K., Skiniotis G. (2017). Nature.

[cit22] Teyssières E., Corre J.-P., Antunes S., Rougeot C., Dugave C., Jouvion G., Claudon P., Mikaty G., Douat C., Goossens P. L., Guichard G. (2016). J. Med. Chem..

[cit23] Pasco M., Dolain C. and Guichard G., in Comprehensive Supramolecular Chemistry II, Elsevier, 2017, pp. 89–125.

[cit24] Checco J. W., Gellman S. H. (2016). Curr. Opin. Struct. Biol..

[cit25] Fremaux J., Mauran L., Pulka-Ziach K., Kauffmann B., Odaert B., Guichard G. (2015). Angew. Chem..

[cit26] Burgess K., Shin H., Linthicum D. S. (1995). Angew. Chem., Int. Ed. Engl..

[cit27] Douat-Casassus C., Pulka K., Claudon P., Guichard G. (2012). Org. Lett..

[cit28] de Graaf C., Donnelly D., Wootten D., Lau J., Sexton P. M., Miller L. J., Ahn J.-M., Liao J., Fletcher M. M., Yang D., Brown A. J. H., Zhou C., Deng J., Wang M.-W. (2016). Pharmacol. Rev..

[cit29] Hargrove D. M., Kendall E. S., Reynolds J. M., Lwin A. N., Herich J. P., Smith P. A., Gedulin B. R., Flanagan S. D., Jodka C. M., Hoyt J. A., McCowen K. M., Parkes D. G., Anderson C. M. (2007). Regul. Pept..

[cit30] Jones B., Buenaventura T., Kanda N., Chabosseau P., Owen B. M., Scott R., Goldin R., Angkathunyakul N., Corrêa Jr I. R., Bosco D., Johnson P. R., Piemonti L., Marchetti P., Shapiro A. M. J., Cochran B. J., Hanyaloglu A. C., Inoue A., Tan T., Rutter G. A., Tomas A., Bloom S. R. (2018). Nat. Commun..

[cit31] Zhang H., Sturchler E., Zhu J., Nieto A., Cistrone P. A., Xie J., He L., Yea K., Jones T., Turn R., Di Stefano P. S., Griffin P. R., Dawson P. E., McDonald P. H., Lerner R. A. (2015). Nat. Commun..

[cit32] Kenakin T., Watson C., Muniz-Medina V., Christopoulos A., Novick S. (2012). ACS Chem. Neurosci..

[cit33] Naylor J., Suckow A. T., Seth A., Baker D. J., Sermadiras I., Ravn P., Howes R., Li J., Snaith M. R., Coghlan M. P., Hornigold D. C. (2016). Biochem. J..

[cit34] Liang Y.-L., Khoshouei M., Glukhova A., Furness S. G. B., Zhao P., Clydesdale L., Koole C., Truong T. T., Thal D. M., Lei S., Radjainia M., Danev R., Baumeister W., Wang M.-W., Miller L. J., Christopoulos A., Sexton P. M., Wootten D. (2018). Nature.

