# Supplementary material for: Ureidopeptide GLP-1 analogues with prolonged activity in vivo via signal bias and altered receptor trafficking
Source: Chem Sci. 2019 Sep 11;10(42):9872–9. doi: 10.1039/c9sc02079a (PMC6977461; doi:10.1039/c9sc02079a)
Supplement: Supplementary file 1 [file SC-010-C9SC02079A-s001.pdf]

Electronic Supplementary Material (ESI) for Chemical Science.
This journal is © The Royal Society of Chemistry 2019

Ureidopeptide GLP-1 analogues with prolonged activity *in vivo* via signal bias and altered receptor trafficking†

Juliette Fremaux,[a] Claire Venin,[a] Laura Mauran,[a] Robert Zimmer,[a] Florian Koensgen,[c] Didier Rognan[c], Stavroula Bitsi,[d] Maria A. Lucey,[e] Ben Jones,[e] Alejandra Tomas,[d] Gilles Guichard,[b*] Sébastien R. Goudreau[a*]

Electronic Supplementary Information

Additional Results

Screening of different urea residues with various side chains

Table S1: Ureido screening of GLP-1 at position 2.

	Sequence	Compound	EC_{50} (nM)[a]	SE (nM)[b]	Potency (%)[c]
**1**	H-H A EGTFTSDVSSYLEGQAA K EFIAWLVKGRG-OH	GLP-1	0.10[d]	0.01[e]	100
**2**	H-H A EGTFTSDVSSYLEGQAA K EFIAWLVKGRG-NH_2	GLP-1-NH_2	0.19	0.06	53
**3**	H-H A^{u} EGTFTSDVSSYLEGQAA K EFIAWLVKGRG-NH_2	GLP-1[A^{u}]^{2}	0.14	0.05	70
**4**	H-H γA EGTFTSDVSSYLEGQAA K EFIAWLVKGRG-NH_2	GLP-1[γA]^{2}	1.4	0.3	7
**5**	H-H A^{c} EGTFTSDVSSYLEGQAA K EFIAWLVKGRG-NH_2	GLP-1[A^{c}]^{2}	0.8	0.3	13
**6**	H-H A^{ua} EGTFTSDVSSYLEGQAA K EFIAWLVKGRG-NH_2	GLP-1[A^{ua}]^{2}	3.4	0.9	3
**7**	H-H G^{u} EGTFTSDVSSYLEGQAA K EFIAWLVKGRG-NH_2	GLP-1[G^{u}]^{2}	0.8	0.2	13
**8**	H-H V^{u} EGTFTSDVSSYLEGQAA K EFIAWLVKGRG-NH_2	GLP-1[V^{u}]^{2}	>10000	N/A	<0.01
**S1**	H-H S^{u} EGTFTSDVSSYLEGQAA K EFIAWLVKGRG-NH_2	GLP-1[S^{u}]^{2}	1.3	0.4	8
**S2**	H-H R^{u} EGTFTSDVSSYLEGQAA K EFIAWLVKGRG-NH_2	GLP-1[R^{u}]^{2}	>10000	N/A	<0.01
**S3**	H-H C^{u} EGTFTSDVSSYLEGQAA K EFIAWLVKGRG-NH_2	GLP-1[C^{u}]^{2}	65	39	0
**S4**	H-H Q^{u} EGTFTSDVSSYLEGQAA K EFIAWLVKGRG-NH_2	GLP-1[Q^{u}]^{2}	>10000	N/A	<0.01
**S5**	H-H E^{u} EGTFTSDVSSYLEGQAA K EFIAWLVKGRG-NH_2	GLP-1[E^{u}]^{2}	>10000	N/A	<0.01
**S6**	H-H I^{u} EGTFTSDVSSYLEGQAA K EFIAWLVKGRG-NH_2	GLP-1[I^{u}]^{2}	>10000	N/A	<0.01
**S7**	H-H L^{u} EGTFTSDVSSYLEGQAA K EFIAWLVKGRG-NH_2	GLP-1[L^{u}]^{2}	>10000	N/A	<0.01
**S8**	H-H K^{u} EGTFTSDVSSYLEGQAA K EFIAWLVKGRG-NH_2	GLP-1[K^{u}]^{2}	>10000	N/A	<0.01
**S9**	H-H F^{u} EGTFTSDVSSYLEGQAA K EFIAWLVKGRG-NH_2	GLP-1[F^{u}]^{2}	0.5	0.1	21
**S10**	H-H T^{u} EGTFTSDVSSYLEGQAA K EFIAWLVKGRG-NH_2	GLP-1[T^{u}]^{2}	3.3	1.0	3
**S11**	H-H W^{u} EGTFTSDVSSYLEGQAA K EFIAWLVKGRG-NH_2	GLP-1[W^{u}]^{2}	2.2	0.6	5
**S12**	H-H G EGTFTSDVSSYLEGQAA K EFIAWLVKGRG-NH_2	GLP-1[G]^{2}	0.24	0.06	42
**9**	H-H G EGTFTSDLSKQMEEEAV R LFIEWLKNGGPSSGAPPPS-NH_2	Exenatide	0.19	0.08	53
**10**	H-H G EGTFTSDLSKQLEEEAV R LFIEWLKNGGPSSGAPPPS-NH_2	$Ex4^{L}$	0.28	0.12	36
**11**	H-H A^{u} EGTFTSDLSKQLEEEAV R LFIEWLKNGGPSSGAPPPS-NH_2	$Ex4^{L}$[A^{u}]^{2}	0.08	0.05	125
**12**	H-H G EGTFTSDLSKQMEEEAV R LFIEWLKNGGPSSGAPPS6K-NH_2	Lixisenatide	0.26	0.11	38
**13**	H-H G EGTFTSDLSKQLEEEAV R LFIEWLKNGGPSSGAPPS6K-NH_2	$Lixi^{L}$	0.06	0.03	164
**14**	H-H A^{u} EGTFTSDLSKQLEEEAV R LFIEWLKNGGPSSGAPPS6K-NH_2	$Lixi^{L}$[A^{u}]^{2}	0.09	0.03	118
**15**	H-H A EGTFTSDVSSYLEGQAA K* EFIAWLVRGRG-OH	Liraglutide	58	20	0.2

[a] GLP-1R potency (EC_{50}). [b] SE on the EC_{50}. [c] Percentage of potency compare to GLP-1-NH_2 (**2**). [d] Mean value of 22 experiments. [e] SEM on the EC_{50}. Unless otherwise stated, the data are EC_{50} values ± SE obtained by non-linear regression on points concentration-response curves performed in duplicates (n=2). GLP-1R: GLP-1 receptor; EC_{50}: half maximal effective concentration; SE: standard error; SEM: standard error of the mean.

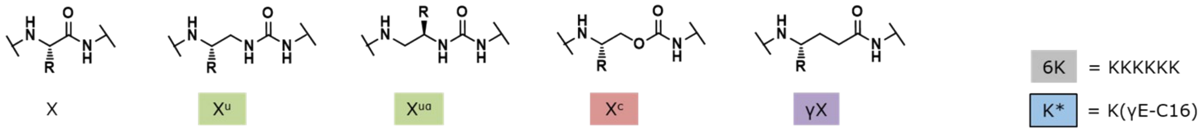

In vitro Mouse plasma stability studies

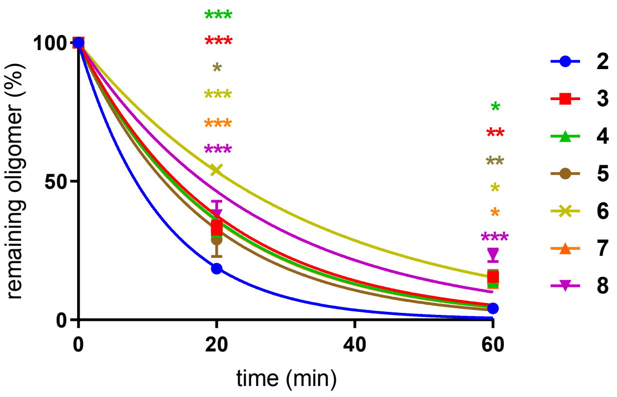

Figure S1: Mouse plasma degradation assay (Method MP1). Stability of selected GLP-1 analogues in mouse plasma. Data are mean ± SEM, n = 3. Statistic by two-way anova and Bonferroni post-test: *p<0.05, **p<0.01, ***p<0.001, comparing peptide **2** to oligomers.

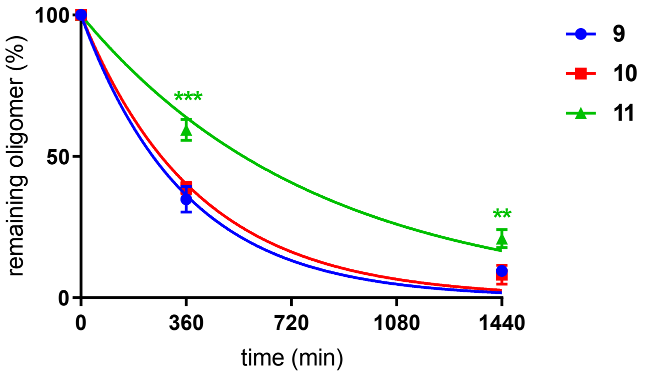

Figure S2: Mouse plasma degradation assay (Method MP2). Stability of selected exenatide analogues in mouse plasma. Data are mean ± SEM, n = 3. Statistic by two-way anova and Bonferroni post-test: *p<0.05, **p<0.01, ***p<0.001, comparing peptide **9** to oligomers.

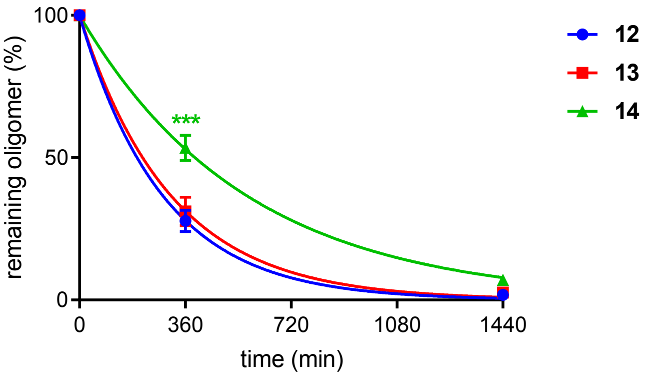

Figure S3: Mouse plasma degradation assay (Method MP3). Stability of selected lixisenatide analogues in mouse plasma. Data are mean ± SEM, n = 3. Statistic by two-way anova and Bonferroni post-test: *p<0.05, **p<0.01, ***p<0.001, comparing peptide **12** to oligomers.

Dose-response study in healthy mice

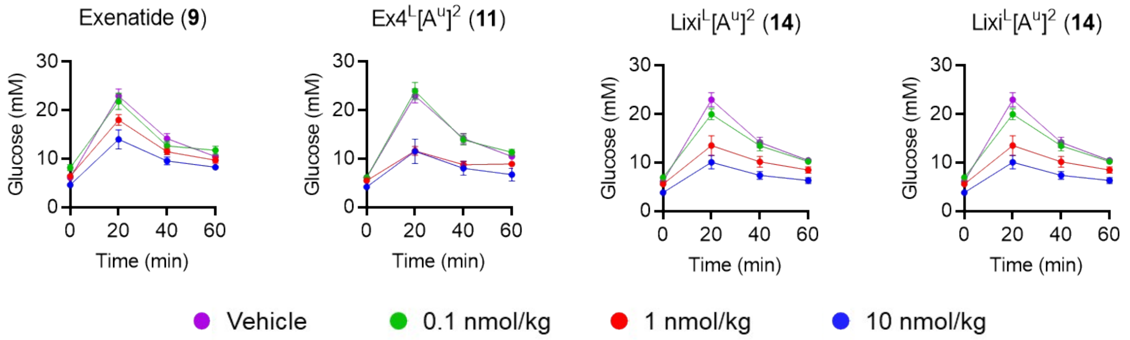

Figure S4 : Dose-response relationships of exenatide (**9**), Lixi^{L} (**13**) and their putative ureidopeptides Ex4^{L}[A^{u}]^{2} (**11**) and Lixi^{L}[A^{u}]^{2} (**14**) in healthy mice (C57BL/6J, male, 25-30 g). IPGTT (2 mmol/kg glucose) performed 6 hours after administration of indicated agonist dose in lean C57Bl/6 mice (n=8). Dosage and formulation: 0.01 to 10 nmol·kg^{-1} in 100 µL saline *i.p.* Fasted 4 h. Data are mean ± SEM.

Study in db/db mice treated over 15 days

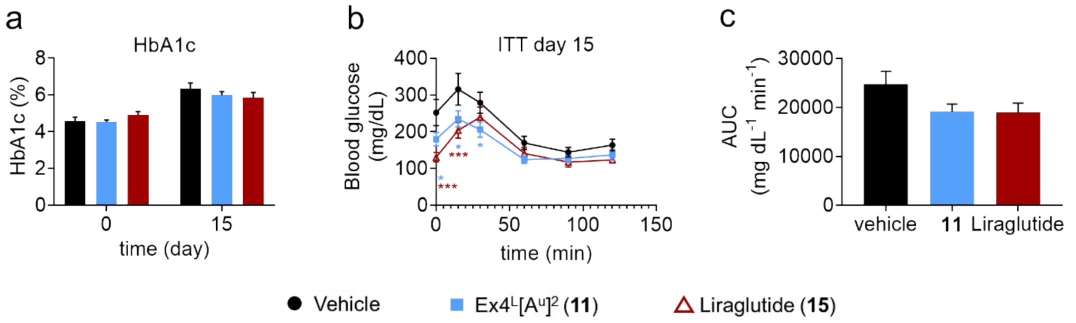

Figure S5 : Comparative pharmacodynamic of Ex4^{L}[A^{u}]^{2} (**11**), Lixi^{L}[A^{u}]^{2} (**14**) and liraglutide (**15**) in db/db mice treated over 15 days. Dosage: 100 µg kg^{-1} (25 nmol kg^{-1}) *s.c.* once a day. Formulation: 20 µg mL^{-1} in PBS 1×. (a) HbA1c level at the beginning (day 0) and at the end (day 15) of the treatment. (b) ITT after 6 h of the dosing at day 15: trace and AUC. ITT: 2 U/kg of insulin *i.p.* at T0. Data are mean ± SEM (n=10). Statistic by two-way anova and Bonferroni post-test: comparing vehicle to oligomers; one way anova with Dunnett's multiple comparison test: comparing vehicle to oligomers. ITT: Insulin tolerance test; AUC: Area under the curve; *s.c.*: subcutaneous; *i.p.:* intra peritoneal.

PK Studies

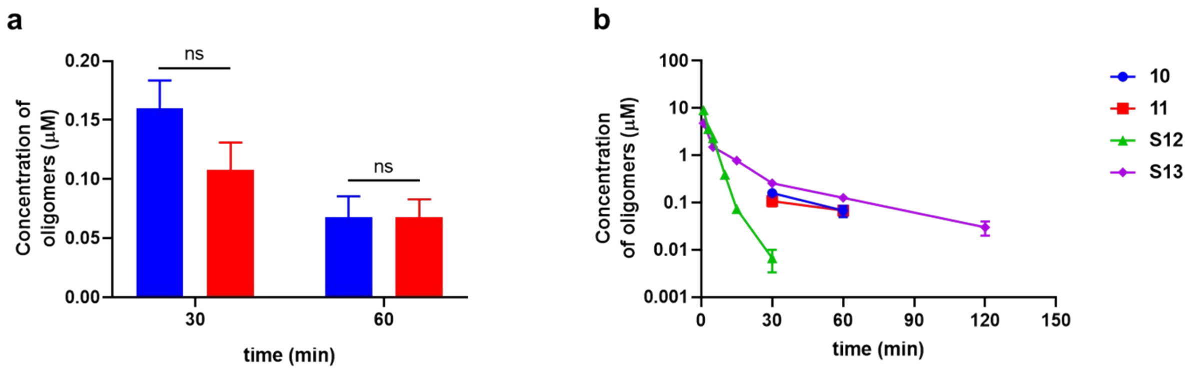

Figure S6 : Pharmacodynamic of Ex4^{L} (**10**) and Ex4^{L}[A^{u}]^{2} (**11**), GLP-1-G^{2}-NH_2 (**S12**)[1] and GLP-1-G^{2}[A^{u}A^{u}A^{u}]$^{28\text{-}31}$ (**S13**)[1] in healthy mice. Mice: male C57BL/6J); dose: 2 mg k^{-1}; formulation: 1 mg mL^{-1} in PBS 1X; route: *i.v.* Data are mean ± SEM (n=4 for **10** and **11**, n=3 for **S13** and **S14**). Statistic by two-way anova and Bonferroni post-test: ns = p>0.05 (non-significant). *i.v.* intra venous. The PK data of **S12** and **S13** were taken from our previously reported work.[1]

SNAP-GLP-1R internalization quantification

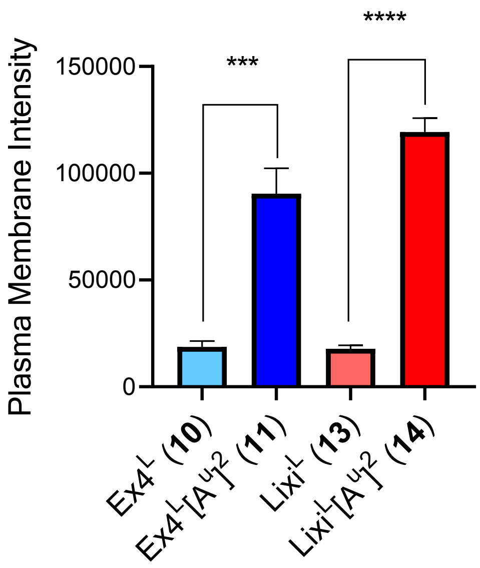

Figure S7 : Quantification of SNAP-GLP-1R internalisation from Figure 6b data: Plasma membrane intensities quantified from area under the curve (AUC) of baseline-subtracted full-width at half-maximum peaks of membrane intensity profiles for the different conditions measured. 3 profiles analysed per image of a total of 2-3 images per experiment, n=3 experiments. ***p<0.001 and ****p<0.0001, one-way ANOVA with Sidak's multiple comparison test. Note that increased plasma membrane intensity corresponds to decreased internalisation propensity.

Methods

Statistical analysis.

GraphPad Prism 7.0 and 8.0 were used for all analyses. Replicate measurements were taken from different samples. Statistical analyses (two-way t-test, two-way ANOVA and Bonferroni post-test, one-way ANOVA with Dunnett's multiple comparison test, two-way ANOVA with Tukey’s test, one-way ANOVA with Sidak’s multiple comparison test) were applied when indicated. P values lower than 0.05 were considered significant.

Synthesis of oligomers 2-14 and S1-S12

General Procedure P1

Oligomers **2-14** and **S1-S12** were synthesized using the following general procedure as previously described:[1]

Sieber resin (≈ 160mg, loading 0.62mmol/g) was swelled in DMF (3 mL) for 30 min. All steps were performed under microwave irradiation. The synthesis were conducted with microwave irradiation using the Liberty Blue™ microwave peptide synthesizer from CEM.

A1: Fmoc deprotection

The *N*-Fmoc protecting group was removed with 20% piperidine in DMF (3 ml) with the standard liberty blue methods.[2]

A2: Coupling of Fmoc-amino acid

N-Fmoc-α amino acid (5 eq. relative to the resin loading) were coupled with PyBOP or DIC/Oxyma (5 eq. relative to the resin loading) and DIEA (10 eq. relative to the resin loading) as coupling reagent using the standard liberty blue methods.[2]

A3: Coupling of activated N_3-building bloc

Each activated monomer (3 eq. relative to the resin loading) was coupled twice using DIEA (10 eq. relative to the resin loading) under microwave irradiation (70°C, 50W, 20 min) in DMF (4 mL).

A4: Reduction of azide group

The reduction of the azido group was performed twice in a mixture of 1,4-dioxane/H_2O (7:3 v/v) (5 mL) with a 1M PMe_3 solution in THF (10 eq. relative to the resin loading) under microwave irradiation (50°C, 50W, 30 min).

A5: Cleavage from the resin

After completion of the synthesis, the resin was transferred into a syringe with a frit, and washed three times with DMF, three times with CH_2Cl_2 and three times with Et_2O. Cleavage from the resin was performed using 95% TFA with 2.5% triisopropylsilane and 2.5% water (3 mL). After 2h the resin was filtered and discarded. Diethyl ether was added to precipitate the oligomer and the solid was triturated and filtrated.

Purification and characterization:

Analytical RP-HPLC analyses were performed on a Dionex U3000SD using a Macherey-Nagel Nucleodur C18ec column (4 x 100 mm, 3 µm) at a flow rate of 1 mL/min with UV detection at 200 nm. The mobile phase was composed of 0.1% (v/v) TFA-H_2O (Solvent A) and 0.1% (v/v) TFA-CH_3CN (solvent B).

Semi preparative purification of all compound was performed on a Dionex U3000SD using a Macherey-Nagel Nucleodur C18ec column (10 x 250 mm, 5 µm) at a flow rate of 4 mL/min with UV detection at 200 nm. The mobile phase was composed of 0.1% (v/v) TFA-H_2O (Solvent A) and 0.1% (v/v) TFA-CH_3CN (solvent B).

LC-MS analyses were carried out on a UHPLC (Agilent 1290 Infinity) coupled to a ESI -MS Tof (Agilent 6230 ESI).

Electrospray ionization mass spectrometry (ESI-MS) experiments were performed on an Agilent 6560 DTIMS-Q-TOF spectrometer (Agilent Technologies, Santa Clara, CA), with the dual-ESI source operated in positive ion mode.

Synthesis of oligomers 15

Procedure P2

N-Fmoc-Lys(Boc)-OH at position 20 was replaced by *N*-Fmoc-Lys(Alloc)-OH and *N*-Fmoc-His(Trt)-OH at position 1 was replaced by *N*-Boc-His(Boc)-OH.

The oligomer was synthesized using procedure **P1.** Then the resin was transferred in a 10 mL syringe, 5 mL of DCM was added and the Alloc group was removed using $Pd(Ph_3)_4$ (30 mg, 0.25 equiv relative to the resin loading) and phenylsilane (135 µL, 1.1 equiv relative to the resin loading) at room temperature for 45 min. After filtration and washes with DMF (2x) and DCM (3x), DCM (5 mL), *N*-Fmoc-Glu-O*tBu* (222 mg, 5 equiv relative to the resin loading), PyBop (260 mg, 5 equiv relative to the resin loading), and DIEA (93 µL, 5 equiv relative to the resin loading) were loaded on the resin and it was shaken for 2 hours at room temperature. Fmoc group was removed with piperidine in DMF (20%), 2 times 20 min. The resin was washed with DMF (2x) and DCM (3x), then palmitoyl chloride (93 µL), and DIEA (260 µL) were loaded on the resin and it was shaken for 2 hours at room temperature. The cleavage, the purification and the characterization were the same as procedure **P1**.

Cell lines and culture

- PathHunter CHO-GLP-1R-βarr2 cells (DiscoverX) were grown in the manufacturer's proprietary medium 6. INS-1 832/3 cells (a kind gift from Prof Christopher Newgard, Duke University, USA) were grown in RPMI supplemented with 11 mM D-glucose, supplemented with 10% FBS, 10 mM HEPES, 2 mM L-glutamine, 1 mM pyruvate, µM β-mercaptoethanol and 1% penicillin/streptomycin.
- Stable SNAP-GLP-1R-expressing INS-1 832/3 cells were generated from INS-1 832/3 cells were endogenous GLP-1R was deleted by CRISPR/Cas9 (a gift from Dr. Jacqui Naylor, MedImmune)[5] by transfection of pSNAP-GLP-1R (Cisbio) and G418 (1 mg/ml) selection, and cultured as above in the presence of G418.
- HEK-293T cells (Multispan inc., lot#.DC1267-062017) were suspended in cell culture media (DMEM 1X + GlutaMAX (Gibco 31966-021)) complemented with FBS 10% (Sigma Aldrich F7524), Pen/Strep 1% (Sigma Aldrich P4333).
- βTC6 cells were suspended in HBSS buffer (Invitrogen) complemented with 20 mM HEPES (pH 7.4) and 500 µM IBMX.

Cyclic AMP measurements

In vitro pharmacology: Affinity (IC_{50})

The affinity of oligomers at the mouse GLP-1 receptor endogenously expressed in βTC6 cells was determined in a radioligand binding assay (performed by Cerep S.A., catalog 2015, ref. 0228) as previously described.[4] Cell membrane homogenates (20 µg protein) are incubated for 120 min at 37°C with 0.025 nM [^{125}I]GLP-1(7-36) in the absence or presence of the test compound in a buffer containing 50 mM Hepes-NaOH (pH 7.4), 120 mM NaCl, 5 mM KCl, 1.2 mM $MgSO_4$, 1 mM EDTA, 0.025% bacitracin and 1% BSA. Nonspecific binding is determined in the presence of 1 µM GLP-1(7-36). Following incubation, the samples are filtered rapidly under vacuum through glass fiber filters (GF/B, Packard) presoaked with 0.3% PEI and rinsed several times with an ice-cold buffer containing 50 mM Tris-HCl and 500 mM NaCl using a 96-sample cell harvester (Unifilter, Packard). The filters are dried then counted for radioactivity in a scintillation counter (Topcount, Packard) using a scintillation cocktail (Microscint 0, Packard). The results are expressed as a percent inhibition of the control radioligand specific binding. The standard reference compound is exenatide (**9**), which is tested in each experiment at several concentrations to obtain a competition curve from which its IC_{50} is calculated using Graphpad Prism.

In vitro pharmacology on HEK-293T cells (EC_{50}: Method C1)

The agonist activity of compounds at the human GLP-1 receptor exogenously expressed in HEK-293T cells (Multispan inc., lot#.DC1267-062017) was determined by measuring their effects on cAMP production using the HTRF detection method (performed by UREkA SARL). The cells were distributed in 384-well microplates at a density of 1.0x10^4 cells/well. Stock solutions of the test compounds or the reference agonist were prepared at a concentration of 1 mM in DMSO. Then, compounds to be tested were diluted in cell culture media and transferred to the plate containing the cells to reach final assay concentrations in the range 10^{-8} to 10^{-15} M. The cells were incubated for 10 min at 5% CO_2 at 37°C. The reaction was then stopped by addition of fluorescence donor (anti-cAMP antibody labeled with europium cryptate) and the fluorescence acceptor (D2-labeled cAMP) mixed in lysis buffer (cAMP Gs-Dynamic kit, Cisbio). After 120 min at room temperature, a microplate reader (F500 Tecan) was used to measure the fluorescence transfer at λex = 337 nm and λem = 620 nm and 665 nm. The ratio of the signal measured at 665 nm on signal measured at 620 nm is used to determine the cAMP concentration. The results are given as a percent of the control response to 10 nM Forskolin. The standard reference agonist is GLP-1-G2-NH2 (peptide 1), which is tested in each experiment at several concentrations to generate a concentration response curve from which its EC50 value is calculated using GraphPad Prism.

In vitro pharmacology on immortalised beta cells (EC_{50}: Method C2)

The agonist activity of compounds at the mouse GLP-1 receptor endogenously expressed in βTC6 cells was determined by measuring their effects on cAMP production using the HTRF detection method (performed by Cerep S.A., catalog 2015, ref. 2181) as previously described.[1,3] The cells were distributed in microplates at a density of 1.5x10^4 cells/well and incubated for 10 min at room temperature in the presence of HBSS (basal control), the test compound or the reference agonist. Following incubation, the cells were lysed and the fluorescence acceptor (D2-labeled cAMP) and fluorescence donor (anti-cAMP antibody labeled with europium cryptate) are added. After 60 min at room temperature, the fluorescence transfer is measured at λex= 337 nm and λem= 620 nm and 665 nm using a microplate reader (Rubystar, BMG). The cAMP concentration is determined by dividing the

signal measured at 665 nm by that measured at 620 nm (ratio). The results are expressed as a percent of the control response to 10 nM GLP-1(7-37). The standard reference agonist is GLP-1(7-37), which is tested in each experiment at several concentrations to generate a concentration-response curve from which its EC50 value and SE are calculated using GraphPad Prism.

In vitro pharmacology on immortalised CHO-GLP-1R cells (EC_{50}: Method C3)

PathHunter CHO-GLP-1R cells were stimulated for 30 min at 37°C with indicated concentration of agonist in serum free medium without phosphodiesterase inhibitors. The reaction was terminated by addition of cAMP detection reagents (cAMP Dynamic 2, Cisbio) and read by HTRF.

β-arrestin-2 recruitment assay

PathHunter CHO-GLP-1R cells were stimulated for 30 min at 37°C with indicated concentration of agonist in serum free medium. The reaction was terminated by addition of PathHunter detection reagents (DiscoverX) and luminescent signal recorded.

Calculation of signal bias

Biased signalling was quantified using a modified form of the operational model.[6] LogR values were calculated for each ligand in each assay, and ΔLogR were determined by subtracting from the corresponding values for URK-062. Bias was then determined as ΔΔLogR by subtracting ΔLogR for cAMP from β-arrestin-2. As all ligands were run in parallel in both pathways, calculations were done on a per-assay basis [3]. Bias was compared statistically by determining the difference between LogR for cAMP and β-arrestin-2 for each ligand and performing a randomised-block 1-way ANOVA on this value.

Insulin secretion *in vitro*

In vitro insulin secretion was performed as previously described.[7] INS-1 832/3 cells were primed at 3 mM glucose overnight before the assay. After washing, cells were added in suspension to the normal growth medium at 11 mM glucose, with or without agonist. In 96 well plates. After overnight incubation, a sample of supernatant was removed and analysed for insulin by HTRF (Insulin Hi Range, Cisbio).

Surface SNAP-GLP-1R labelling and internalization

SNAP-GLP-1R-expressing INS-1 832/3 cells were labeled at 37°C with 1 µM of SNAP-Surface 549 fluorescent probe (New England Biolabs) in full media prior to being stimulated with 10 nM ligands for 30 minutes, fixed in 4% paraformaldehyde, mounted in Prolong Diamond antifade reagent with 4,6-diamidino-2-phenylindole (Life Technologies), and imaged with a Zeiss LSM-780 inverted confocal laser-scanning microscope in a 63x/1.4 numerical aperture oil-immersion objective from the Facility for Imaging by Light Microscopy (FILM) at Imperial College London, and analyzed in Fiji.

Mouse plasma stability (Method MP1)

Stock solutions of the oligomers were prepared at a concentration of 250 µM in mQ water. The oligomer was then diluted 1/50 with a solution of plasma/PBS pH 7.4 (1/1) to afford a final concentration of 5µM and incubated at 37°C. (4µL of the stock solution was diluted with 196 µL of plasma/PBS, pH 7.4, 1/1) Each compound was also incubated in the absence of plasma (196 µL of H2O/PBS, pH 7.4, 1/1)). At the indicated time (20min and 60min) an aliquot of 70 µL was removed from each experimental reaction and pipetting into 175 µL of Acetonitrile at 0°C to quench the reaction. (t = 0 min was determined using the reaction without plasma). The samples were frozen at -80°C before analysis. The frozen sample were defrosted, stirred with a vortex 5 min and finally centrifuged 5 min at 16°C. The supernatant was analyzed by LC-MS. The time course of oligomer degradation was determined by integrating the area of the peak in the extracted ion chromatogram.

Mouse plasma stability (Method MP2)

Stock solutions of the oligomers were prepared at a concentration of 250 µM in DMSO. The oligomer was then diluted 1/50 with a solution of plasma/PBS pH 7.4 (1/1) to afford a final concentration of 5µM and incubated at 37°C. (4µL of the stock solution was diluted with 196 µL of plasma/PBS, pH 7.4, 1/1) Each compound was also incubated in the absence of plasma (196 µL of H2O/PBS, pH 7.4, 1/1)). At the indicated time (20min and 60min) an aliquot of 70 µL was removed from each experimental reaction and pipetting into 175 µL of Acetonitrile at 0°C to quench the reaction. (t = 0 min was determined using the reaction without plasma). The samples were frozen at -80°C before analysis. The frozen sample were defrosted, stirred with a vortex 5 min and finally centrifuged 5 min at 16°C. The supernatant was analyzed by LC-MS. The time course of oligomer degradation was determined by integrating the area of the peak in the extracted ion chromatogram.

Mouse plasma stability (Method MP3)

Stock solutions of the oligomers were prepared at a concentration of 250 µM in DMSO. The oligomer was then diluted 1/50 with a solution of plasma/PBS pH 7.4 (1/1) to afford a final concentration of 5µM and incubated at 37°C. (4µL of the stock solution was diluted with 196 µL of plasma/PBS, pH 7.4, 1/1) Each compound was also incubated in the absence of plasma (196 µL of H2O/PBS, pH 7.4, 1/1)). At the indicated time (20min and 60min) an aliquot of 70 µL was removed from each experimental reaction and pipetting into 10 µL of HCL 12N solution. The remaining mixture was then pipetting into 165 µL of Acetonitrile at 0°C to quench the reaction. (t = 0 min was determined using the reaction without plasma). The samples were frozen at -80°C before analysis. The frozen sample were defrosted, stirred with a vortex 5 min and finally centrifuged 5 min at 16°C. The supernatant was analyzed by LC-MS. The time course of oligomer degradation was determined by integrating the area of the peak in the extracted ion chromatogram.

Animals.

For the pharmacodynamics studies performed by Physiogenex S.A.S., mice were housed in ventilated and enriched housing cages (310 x 125 x 127 mm^3) throughout the experimental phase. The mice were housed in groups of 3 animals during the study, on a normal 12 hours light cycle (at 8:00 pm lights off), 22 ± 2 °C and 50 ± 10 % relative humidity. A standard chow diet (RM1 (E) 801492, SDS) and tap water were provided ad libitum. All animal protocols done by Physiogenex S.A.S were reviewed and approved by the local (Comité régional d'éthique de Midi-Pyrénées) and national (Ministère de l'Enseignement

Supérieur et de la Recherche) ethics committees (protocol number 05049-06). For the dose-response study, animal procedures were approved by British Home Office under the UK Animal (Scientific Procedures) Act 1986 (Project Licence 70/7596). Lean male C57Bl/6J mice (6-8 weeks of age, body weight 25-30 g, obtained from Charles River) were maintained at 21-23°C and light-dark cycles (12:12h schedule, lights on at 07:00). *Ad libitum* access to water and normal chow (RM1, Special Diet Services) was provided unless otherwise stated. Mice were housed in groups of five. For the pharmacokinetics studies performed by TechMedILL service (PCBIS platform, CNRS UMS3286), mice were housed in polycarbonate cages (PCT2L12SHT, Allentown) enriched with play tunnels throughout the experimental phase. The mice were housed in groups of 9 animals during the study, under controlled environment (22 ± 1 °C) with a relative humidity (50 ± 10 %) and a normal 12 hours light cycle (at 8:00 pm lights off). A standard chow diet (A04, SAFE, France) and tap water were provided ad libitum. All animal protocols done by TechMedILL service were reviewed and approved by the agriculture ministry regulating animal research in France (Ethics regional committee for animal experimentation Strasbourg, APAFIS 1341#2015080309399690).

IPGTT experiments in healthy mice (IPGTT 3 h and 6 h).

After the acclimation period of 5 days, 20-25 g male C57Bl/6J mice 8 weeks old (Charles River laboratories) were randomized into groups (6 mice per group) according to their body weight. The mice were acutely treated with vehicle or 1 µg per mouse (10 nmol kg^{-1}) of oligomers via *i.v.* injections (formulation: 4 µg mL^{-1} in PBS 1×) IPGTTs (glucose 2 g kg^{-1} *i.p.*) were performed after 3 or 6 hours of dosing with a fasting period of 6 hours. Blood glucose was measured before the administration of glucose and after at 30, 60, 90 and 120 min.

Fasted Blood Glucose and IPGTT experiments in healthy mice (IPGTT 9 h).

After the acclimation period of 5 days, 20-25 g male C57Bl/6J mice 8 weeks old (Charles River laboratories) were randomized into groups (6 mice per group) according to their body weight. The mice were acutely treated with vehicle or 1 µg per mouse (10 nmol kg^{-1}) of oligomers via *i.v.* injections (formulation: 4 µg mL^{-1} in PBS 1×). The mice were fasted and blood glucose was measure before dosing (T0) and after 2, 4, 6, 8 and 9 hours. An IPGTT (glucose 2 g kg^{-1} *i.p.*) was performed after 9 hours of dosing with a fasting period of 9 hours. Blood glucose was measured before the administration of glucose and after 30, 60 and 90 min.

Fed Blood Glucose experiments in healthy mice (30 h).

After the acclimation period of 5 days, 20-25 g male C57Bl/6J mice 8 weeks old (Charles River laboratories) were randomized into groups (6 mice per group) according to their body weight. The mice were acutely treated with vehicle or 1 µg per mouse (10 nmol kg^{-1}) of oligomers via *i.v.* injections (formulation: 4 µg mL^{-1} in PBS 1×) Fed blood glucose was measure before dosing (T0) and after 3, 6, 18, 24, 27 and 30 hours. An IPGTT (glucose 2 g kg^{-1} *i.p.*) was performed after 9 hours of dosing with a fasting period of 9 hours. Blood glucose was measured before the administration of glucose and after 30, 60 and 90 min.

Dose-response study in healthy mice.

Mice were lightly fasted (4 hours) before IP administration of varying doses of each agonist or vehicle (100 µL saline). After a further 6 hours, blood glucose was measured (GlucoRx) from the tail followed by IP administration of 2 g/kg 20% glucose. Blood glucose was monitored at 20, 40 and 60 minute time-points. To derive the ED_{50} (dose required to give a 50% maximal response), 3-parameter fitting was performed on the area-under-curve for each set of data.

Study in db/db mice (15 days).

After the acclimation period of 5 days, male db/db mice 8 weeks old (Charles River laboratories) were randomized into groups (n=10) according to their body weight, HOMA-IR and HbA1C measured after an overnight fast and 8 non diabetic mice were excluded. Mice were chronically treated during 15 days via *s.c.* route (once daily) at around 8AM with the vehicle or with 100 µg kg^{-1} (25 nmol kg^{-1}) of the oligomers. Formulation: 20 µg mL^{-1} in PBS 1×. On the 7th day of treatment, fed blood glucose was measured at 1, 2, 3, 6 and 24 h after dosing. On the 12th day of treatment, mice were fasted 6 hours and an OGTT (glucose 1 g kg^{-1} *p.o.*) was performed 6 hours (2 PM) after the dosing. Blood glucose was measure 30 and 0 min before the glucose injection and after 15, 30, 60, 90, and 120 min. Plasma insulin was measure 30 min before the glucose injection and 15 min after. On the 15th day of treatment, mice were fasted 6 hours and an ITT (insulin 2 U kg^{-1} *i.p.*) was performed 6 hours (2 PM) after the dosing. Blood glucose was measure before insulin injection and after 15, 30, 60, 90, and 120 min. Blood samples were collected for measuring HbA1c just before the start of the treatments and at the end of the treatment period.

Pharmacokinetics.

Fifteen mice (male C57BL/6J mice (Janvier Labs, France) 9 weeks old, 20-25 g) were treated with GLP-1 analogues via *i.v.* injections (2 mg kg^{-1}) formulated at 1 mg mL^{-1} in PBS 1×. After different time points mice were sacrificed and blood sample were collected. The plasma was separated by centrifugation and the samples were frozen at -80°C before analysis. A volume of 400 µL of each sample of plasma was mixed with 1 ml of acetonitrile to precipitate the protein and extract the compound. The sample were then vortexed and centrifuged (15 000 × g, 5 min, 16 °C) to sediment the precipitated protein. The supernatant was analysed by LC-MS/MS using a UHPLC coupled to LC-MS 8030 Shimadzu triple quadrupole.

Molecular dynamics simulation of GLP-1R in complex with ureidopeptide 3

A three dimensional model of human GLP-1 receptor (GLP-1R) was obtained from the cryo-EM structure of rabbit GLP1-R in complex with human GLP-1 (PDB ID 5VAI)[8]. Human GLP-1R which shares 92% sequence identity with the rabbit template was directly obtained by mutating corresponding residues in SYBYL X2.1.1 (Cetera Inc, Princeton, NJ). GLP-1 bound peptide was then modified to ureido peptide 3 by substituting Ala^u for Ala2. Dihedral angle values for main chain atoms of Ala^u2 were manually assigned to that of a right-handed 2.5 helix, in agreement with our previous work on the NMR structure elucidation of an ureido heptamer in methanol[9]. Hydrogen atoms were added with AMBER16 (University of California, San Francisco). Previously developed AMBER parameters were used to model ureido alanine[9]. The CHARMM-GUI interface[10] was used to orient the receptor-peptide complex in a 114 * 113 *136 Å hydrated phospholipid bilayer (60 cholesterol + 120 POPC +

120 POPE) at a 0.150 M NaCl concentration (75 Na+ and 79 Cl-) and containing 27,692 TIP3P water molecules (Figure S8).

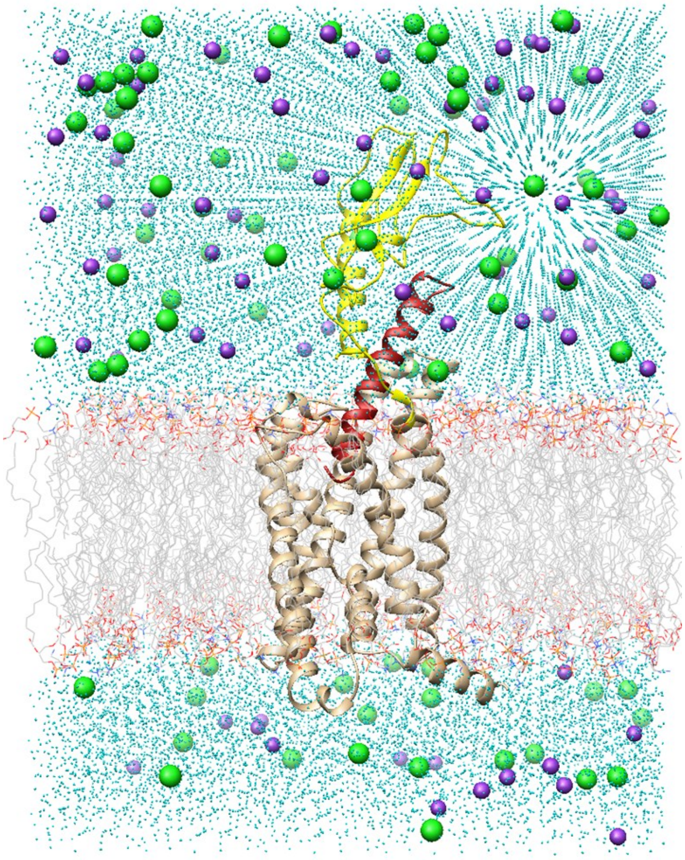

Figure S8: Starting orientation of human GLP-1R (transmembrane domain, tan ribbons; extracellular domain, yellow ribbons) bound to ureido peptide 3 (fire brick ribbons), in a phospholipid bilayer (cpk-colored lines) hydated by a 10 Å shell of water molecules (cyan dots) at 0.150 M NaCl concentration (Na^+, purple spheres; Cl^-, green spheres)

The full system containing 125,694 atoms was first refined in AMBER16 using the ffSB14 force field[11] and the lipid14 parameter set.[12] A harmonic positional restraint of 20 kcal.mol^{-1}A^{-2} was first set on GLP-1R and peptide 3 for 10,000 steps of steepest descent followed by 20,000 steps of conjugate gradient minimization. A non-bonded cut off value of 10 Å was used to compute non-bonded interactions with the particle-mesh Ewald (PME) to handle long-range electrostatic interactions with periodic boundaries. A second minimization step similar to the first one was performed while reducing the positional restraints to a value of 7 kcal.mol^{-1}A^{-2} for the receptor-peptide atoms, excepted for the His1-Ala^{u}2 N-terminal part of the ureido peptide for which the restraint was looser (1.5 kcal.mol^{-1}A^{-2}). A last fully unrestrained minimization protocol (10,000 steps of steepest-descent + 20,000 steps of conjugate gradient) was then applied to the full system.

All atoms were then submitted to a molecular dynamics (MD) protocol consisting of 10 iterative equilibrium steps of 25 fs each using a time step of 1 fs, a Langevin thermostat and a collision frequency of 1 ps^{-1}. In the first three steps, a constant volume was applied and the temperature gradually increased from 100 to 300K while restraining differently the protein-peptide complex and the ions (restraint of 10 kcal.mol^{-1}A^{-2}) from the phospholipid bilayer and water molecules (restraint of 2.5 kcal.mol^{-1}A^{-2}). In the next four steps, a constant pressure of 1 atm was applied with a Berendsen barostat, a isotropic position scaling, and a pressure relaxation time of 2.0 ps. The SHAKE algorithm

was applied to constrain bonds involving hydrogen atoms and harmonic positional restraints were gradually decreased from 5 to 0.5 kcal.mol^{-1}A^{-2} (protein + peptide), and from 2.5 to 0.1 kcal.mol^{-1}A^{-2} for the hydrated phospholipid bilayer. In the last three equilibrium steps of NPT dynamics, the phospholipid bilayer was free to move, while the restraints on the protein-peptide complex were decreased from 1 to 0.5 kcal.mol^{-1}A^{-2} and lastly removed. The system was next simulated for three final steps of 2 ns NPT dynamics each, where the time step was set to 2 fs and the skinnb parameter set to a value of 5 Å. Last, 5 independent production NPT dynamics trajectories of 60 ns each were run to sample the conformational space of the receptor-peptide complex in a hydrated phospholipid bilayer. All simulations were run on a NVIDIA Tesla K80 GPU accelerator using the CUDA version of AMBER PMEMD module.

Recording statistics along the entire trajectory for density, temperature, pressure and total energy shows that all simulations were well equilibrated (Figure S9).

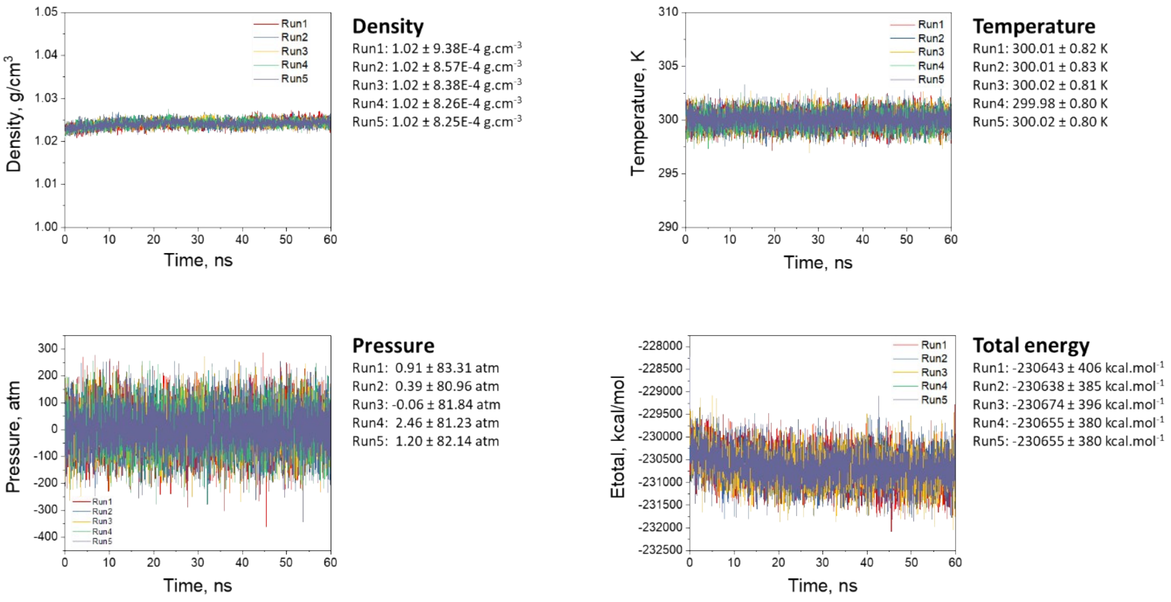

Figure S9: Time course and average value for density, temperature, pressure and total energy along the 60 ns production molecular dynamics trajectories of GLP1-R in complex with the ureido peptide 3 in a fully hydrated phospholipid bilayer.

Atomic coordinates were saved every 20 ps, therefore producing a set of 3,000 snapshots for each trajectory out of which the last 10 ns (50-60 ns simulation time) were kept for producing a time-averaged structure (Figure S10).

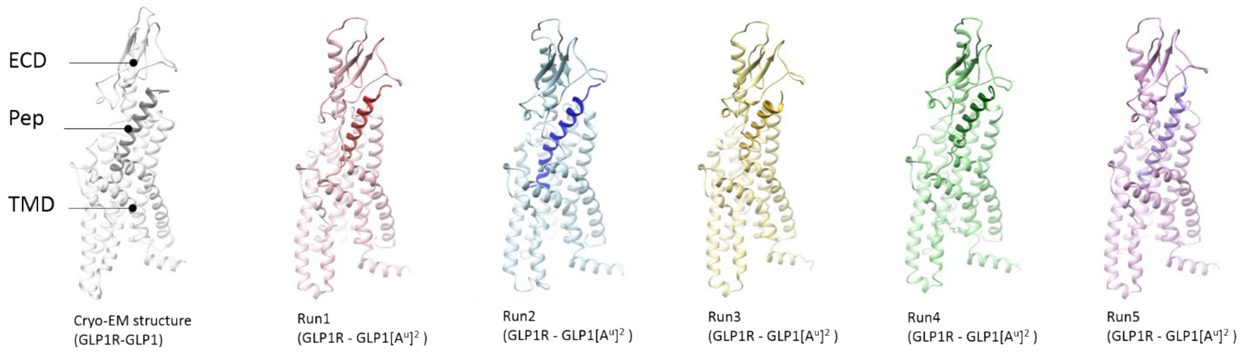

Figure S10: Time-average structure of GLP-1R in complex with ureido peptide 3 (GLP1[A^u]2) for 5 independent molecular dynamics simulations. Receptor and peptide are displayed by ribbons. The relative position of the peptide with respect to the extracellular (ECD) and transmembrane domain (TMD) of GLP-1R is indicated for the GLP-1R/GLP1 cryo-EM structure.

Root-mean square deviations (rmsd) of main chain atomic coordinates to that of the starting conformation shows that neither the GLP-1R nor the peptide main chain atoms encounter major conformational changes (rmsd < 2.0 Å), excepted for the fifth run for which the ureido peptide is drifting away from its initial positions, mostly at its C-terminal end (Figure S11). Altogether, the first four trajectories are remarkably stable considering the large size of the simulated system (125,964 atoms).

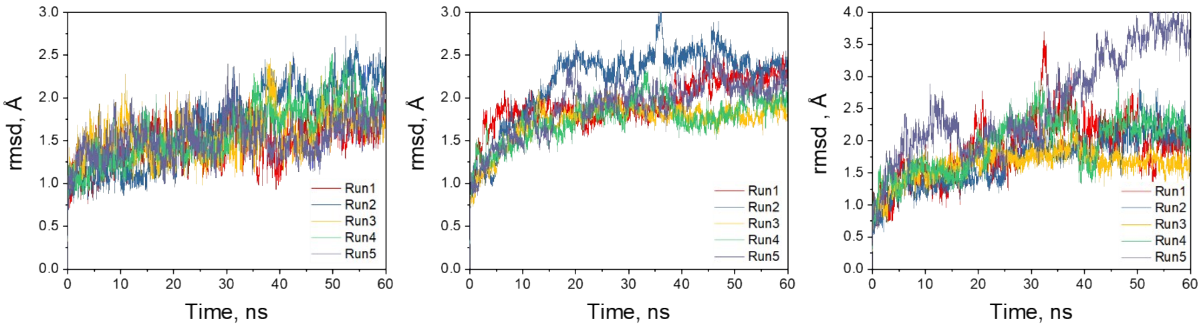

Figure S11: rms deviations of main chain atomic coordinates for the extracellular domain (ECD: Thr24-Pro137), the transmembrane domain (TMD: Glu138-Arg421) and the ureido peptide 3 (His1-Gly31).

Receptor-peptide non-bonded interactions were computed with the in-house developed IChem toolkit[13] and converted into a receptor-ligand interaction fingerprint[14] for ease of comparisons of MD structures with respect to the GLP-1R/GLP-1 cryo-EM template (Figure S12)

ECD | TMD

	S31	L32	W33	T65	D67	E68	R102	K113	S116	S117	R121	E128	E133	S136	E138	E139
GLP1	00000	00000	10000	00000	00000	00000	00000	00000	00000	00000	10000	01010	00000	00000	01000	00000
Run1	10000	10000	00000	00000	00000	01010	00000	00000	10000	10000	10000	00000	00000	00000	00000	01000
Run2	10000	00000	00000	00000	00000	01010	00000	00000	10000	10000	10000	00000	01000	00000	00000	01000
Run3	10000	10000	00000	00000	01000	01010	00000	10100	00000	00000	10000	00000	00000	00000	00000	01000
Run4	10000	10000	00000	00000	01000	01010	11000	00000	00000	00000	10000	00000	00000	01000	00000	00000
Run5	10000	10000	00000	10000	00000	00000	10000	00000	10000	10000	10000	01000	00000	00000	00000	01000

TMD

	Y145	Y152	R190	K197	Q234	Y241	C296	W297	T298	R299	N300	W306	E364	F367	R376	E387
GLP1	00000	10000	10100	00000	10000	01000	00000	01000	10000	10000	10000	10000	00000	00000	10100	00000
Run1	10000	10000	10100	00000	10000	00000	01000	01000	00000	10000	00000	00000	01010	00000	10100	01000
Run2	10000	10000	10100	00000	00000	00000	01000	00000	10100	10100	00000	00000	01010	00000	10100	01000
Run3	00000	10000	10100	10000	00000	00000	00000	01000	00000	10100	00000	00000	01010	00000	10100	01000
Run4	00000	10000	10100	00000	00000	00000	01000	01000	10000	10100	00000	00000	01010	01000	10100	01000
Run5	10000	10000	10100	00000	10000	00000	01000	01000	00000	10000	00000	00000	01010	00000	10100	01000

Figure S12: Protein-peptide interaction fingerprint for the GLP-1R/GLP-1 cryo-EM structure (GLP1) and the 5 time-averaged molecular dynamics trajectory of GLP-1R in complex with ureido peptide 3 (Run1 to Run5). For each peptide-lining residue, a bit is switched on (1) if a polar non-bonded interaction with GLP-1R is observed. The location of the GLP-1R residue in either the extracellular (ECD) or transmembrane domain (TMD) is indicated above the sequence. 5 bits are registered for each residue and color-coded as follows: hydrogen bond with GLP-1R donor (cyan), hydrogen bond with peptide donor (red), ionic bond with GLP-1R positively charged (purple), ionic bond with GLP-1R negatively charged (magenta), metal chelation (green). Interactions are described from topological descriptors (distances, angles) using previously defined settings.[14]

Analysis of the interaction fingerprints reveals that most of key interactions seen in the reference cryo-EM structure between GLP-1R and GLP-1 are conserved in the five MD trajectories of the ureido peptide bound to the same receptor. As to be expected, interactions of the deeply buried N-terminal residues to the TMD are much better conserved than that of the more flexible C-terminal end to the GLP-1R ECD. Notably key hydrogens bonds to Tyr152, Arg190 and Arg299[8] are strictly conserved across the five MD trajectories. Specific interactions, not observed with GLP-1, are also detected upon binding of the ureido peptide. The most significant concerns the N-terminal tripeptide residues (His1-Ala^{u}2-Glu3) and are a direct consequence of the Ala^{u}2 to Ala substitution: (i) an ionic bond between the N-terminal ammonium of His1 and Glu364, (ii) an hydrogen bond of the N nitrogen of Ala^{u}2 to Glu387, (iii) an hydrogen bond of Glu3 side chain to Tyr152.

Synthesis of Azido building block and Fmoc-γ-amino acids

The azido building blocks for the Ala^{u}, Ala$^{u\alpha}$, Ser^{u}, Glu^{u}, Leu^{u}, Lys^{u}, Phe^{u}, Trp^{u}, Val^{u}, Ile^{u} residues were synthesized as previously reported.[1,15–18]

1-(azidosulfonyl)-4,5-dihydro-1H-imidazol-3-ium chloride

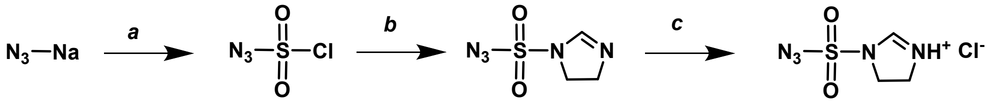

a: SO_2Cl, CH_3CN, 0°C, N_2; ***b:*** Imidazole, CH_3CN, 0°C; ***c:*** HCl (2M) in Et_2O, 0°C

Figure S13 : Synthesis of Imidazolium salt

a: Sodium azide (14.0 g, 215 mmol) was suspended in CH_3CN (150 mL) at 0 °C under N_2 atmosphere. Sulfuryl chloride (17.4 mL, 215 mmol) was added dropwise over 10 min. The mixture was stirred overnight from 0°C to room temperature.

b:The mixture was cooled down to 0°C, and imidazole (27.9 g, 409 mmol) was added. The mixture was stirred for 2.5 h at 0 °C. EtOAc (100ml) and H_2O was added. The organic layer was washed with H_2O and $NaHCO_3$ (sat), dried over $MgSO_4$ and concentrated to remove half of the solvent.

c: The mixture was cooled to 0 °C and the compound was precipitated upon addition of HCl (2M) in Et_2O. The salt was filtered, washed with Et_2O and dried under vacuum (26 g, 125 mmol, 58%).

Monomer M1; 2,5-dioxopyrrolidin-1-yl (2-azidoethyl)carbamate (N_3-G^{u})

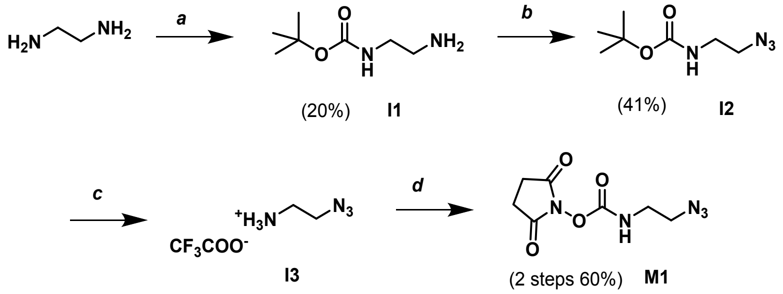

a: Boc_2O, DCM, 0°C; ***b:*** K_2CO_3, $CuSO_4.5H_2O$, Imidazolium salt, CH_3CN/H_2O; ***c:*** TFA; ***d:*** DIEA, DSC, DCM, 0°C

Figure S14 : Synthesis of monomer **M1**

a: Ethylenediamine (6.00 g, 99.8 mmol) was dissolved in anhydrous DCM (100 mL) under N_2 atmosphere at 0 °C. Di-tert-butyl dicarbonate (6.53 g, 29.9 mmol) dissolved in DCM (20ml) was added dropwise over 1 h. The mixture was stirred overnight. Organic phase was washed with brine, dried over $MgSO_4$ and concentrated.

b: **I1** (3.1 g, 19.3 mmol) was dissolved in CH_3CN/H_2O (50:50, 150 ml). Potassium carbonate (4.01 g, 29.0 mmol), Copper(II) sulfate pentahydrate (48.0 mg, 193 umol) and imidazolium salt (4.87 g, 23.2 mmol) were added. The mixture was stirred 1.5 h at RT. EtOAc was added and the layers were separated. The aqueous layer was extracted with EtOAc. Organic layers were combined and washed with $KHSO_4$, brine, dried over $MgSO_4$ and concentrated. The compound was purified by flash chromatography on silica gel (liquid loading). Eluent Cyclo/EtOAc 90:10 and then 80:20.

c: **I2** (1.5 g, 12.3 mmol) was dissolved in Trifluoroacetic acid (30 mL) at 0 °C and stirred 2 h. TFA was evaporated and then coevaporated with cyclohexane. The compound was dissolved in H_2O and lyophilized.

d: N,N'-Disuccinimidyl carbonate (1.99 g, 7.8 mmol) was dissolved in anhydrous DCM (20 mL) and cooled to 0°C. TFA salt (**I3**) (1.3 g, 6.5 mmol) and N,N-Diisopropylethylamine (2.15 mL, 12.3 mmol) dissolved in anhydrous DCM (100 mL) were added and the mixture was stirred for 3 h at room temperature. The organic phase was washed with $KHSO_4$ (1M), brine, dried over $MgSO_4$ and concentrated. The compound was triturated in hexane to afford the monomer **M1** as a white powder (1.1 g, 4.8 mmol, 4.8% yield).

Melting point (*M.p.*) 82.2 °C; **^{1}H NMR** ($CDCl_3$, 300 MHz) δ: 6.00 (s, 1H), 3.51 (m, 4H), 2.87 (s, 4H); **^{13}C NMR** (75 MHz, $CDCl_3$) δ: 169.95, 151.57, 50.41, 41.18, 25.48; **HRMS** (*m/z*) calcd for $C_7H_9N_5O_4Na^+$ [M + Na]$^+$ 250.0547, found 250.0542.

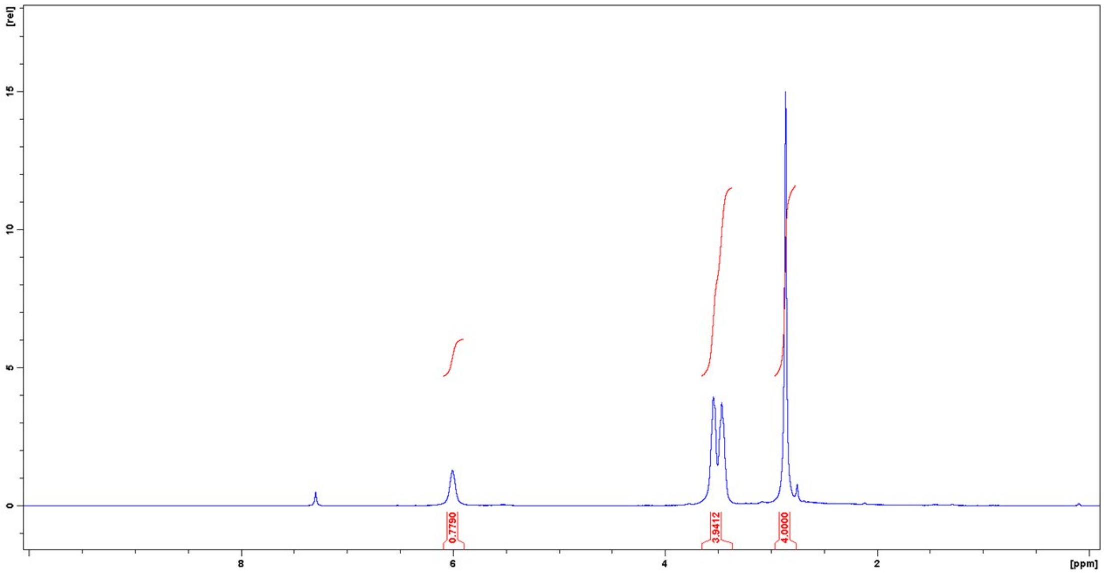

Figure S15 : ^{1}H NMR of monomer **M1** in $CDCl_3$ (300 MHz)

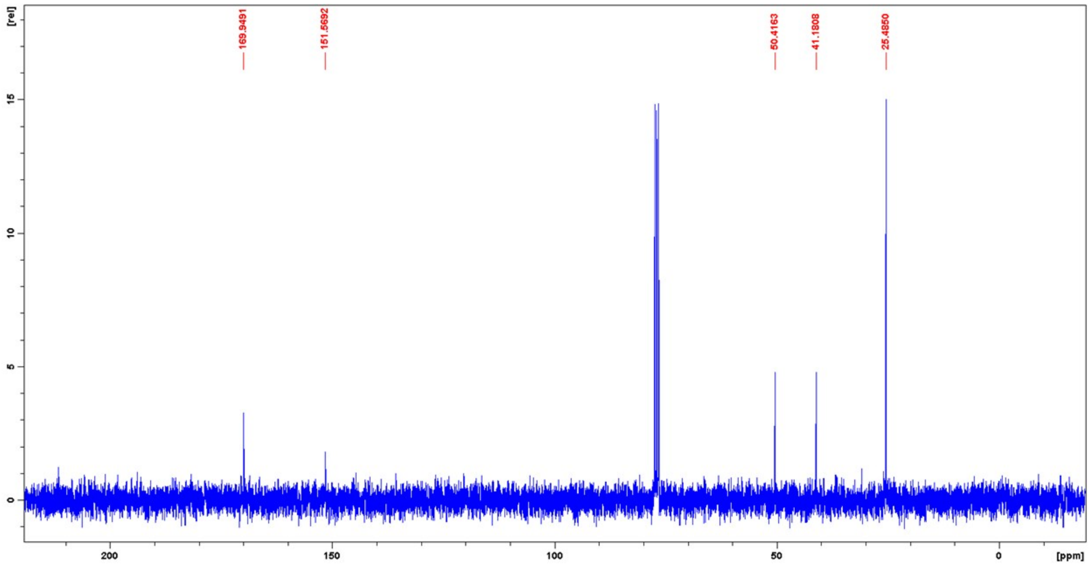

Figure S16 : ^{13}C NMR of monomer **M1** in $CDCl_3$ (75 MHz)

Monomer M2; 2,5-dioxopyrrolidin-1-yl (S)-(2-azido-3-(tritylthio)propyl)carbamate; (N_3-C[u])

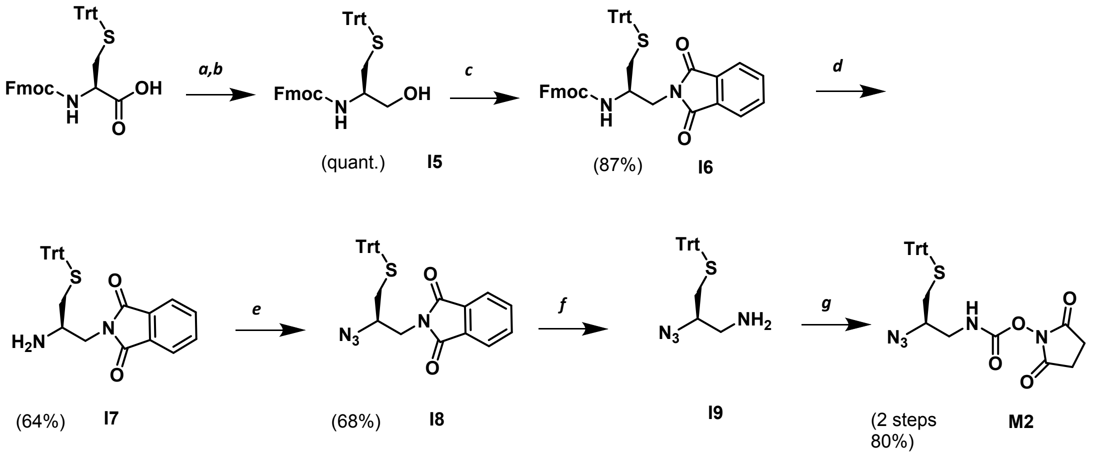

a: iBuOCOCl, NMM, THF, -10°C; *b:* $NaBH_4$, THF, H_2O, 0°C; *c:* PPh_3, DIAD, Phtalimide, THF, 0°C; *d:* piperidine, dioxane; *e:* K_2CO_3, $CuSO_4.5H_2O$, Imidazolium salt, CH_3CN/H_2O ; *f:* $N_2H_4.H_2O$, MeOH, 70°C, *g:* DSC, EtOAc, 0°C

Figure S17 : Synthesis of monomer **M2**

a: Fmoc-L-Cys(Trt)-OH (5.00 g, 8.5 mmol) was dissolved in dry THF (in 500 mL double neck flask) at (-10 °C, ice + brine) under N_2 atmosphere. 4-Methymorpholine (1.0 mL, 9.4 mmol) was added
Isobutyl chloroformate (1.2 mL, 9.0 mmol) was added dropwise via an addition funnel. The mixture was stirred 45 min at -10 °C.
The white precipitate was filtered and washed with THF.
b: Sodium borohydride (600 mg, 17.1 mmol) was dissolved in 5 ml of H_2O and the previous filtrate was added dropwise at 0 °C.
The mixture was stirred for 3 h at RT.
THF was evaporated and the compound was solubilized in EtOAc, washed with $KHSO_4$ (1M), $NaHCO_3$ (sat), brine, dried over $MgSO_4$ and concentrated.
c: **I5** (10.59 g, 8.5 mmol) was dissolved in anhydrous THF (50 ml) at 0 °C and triphenylphosphine (2.68 g, 10.2 mmol) and Phtalimide (1.5 g, 10.2 mmol) were added. DIAD (2 ml, 10.2 mmol) was added dropwise at 0 °C and the mixture was stirred for 48h at RT.
The solvent was evaporated and the compound was purified by flash column chromatography on silica gel. Eluent Cyclohexane/EtOAc 90:10 and then 80:20.
d: **I6** (5.1 g, 7.4 mmol) was dissolved in dioxane and piperidine (1.1 ml, 11.1 mmol) was added. The mixture was stirred overnight at room temperature.
The solvent was evaporated and the compound was purified by flash chromatography on silica gel. Eluent DCM/MeOH; gradient 98:2 to 90:10.
e: **I7** (2.27 g, 4.7 mmol) was dissolved in CH_3CN/H_2O (50:50, 100 ml). Potassium carbonate (0.9 g, 7.2 mmol), Copper(II) sulfate pentahydrate (12.0 mg, 50 umol) and imidazolium salt (1.19 g, 5.7 mmol) were added. The mixture was stirred 1.5 h at RT. EtOAc was added and the layers were separated. The aqueous layer was extracted with EtOAc. The organic layers were combined and washed with $KHSO_4$ and brine, dried over $MgSO_4$ and concentrated. The compound was purified by flash chromatography on silica gel (liquid loading). Eluent Cyclo/EtOAc 90:10 and then 80:20.
f: **I8** (1.62 g, 3.2 mmol) was dissolved in MeOH (15 ml) with DCM (4 ml). Hydrazine monohydrated (470 µL, 9.6 mmol) was added and the mixture was stirred at 70 °C for 3 h. The white precipitate was filtrated and washed with EtOAc. The MeOH was evaporated. The compound was dissolved in EtOAc, washed with water and brine and then dried over $MgSO_4$. The compound was used as is in the next step without evaporation.

g: N,N'-Disuccinimidyl carbonate (0.82 g, 3.2 mmol) was suspended in EtOAc (20 mL) at 0 °C. **I9** (3.2 mmol) previously dissolved in EtOAc (150 mL) was added dropwise at 0 °C and the mixture was stirred for 2 h at room temperature.
Half of the solvent was evaporated. The organic layer was washed with $KHSO_4$ (1M), and brine, dried over $MgSO_4$ and concentrated. The mixture was triturated in hexane to afford the monomer **M2** as a white powder (1.32g, 2.56 mmol, 30% yield).

Melting point (*M.p.*) 65.2 °C; **^{1}H NMR** ($CDCl_3$, 300 MHz) δ: 7.55-7.21 (m, 15H), 5.43 (m, 1H), 3.35-3.24 (m, 1H), 3.06-2.95 (m, 1H), 2.86 (m, 1H), 2.84 (s, 4H), 2.57-2.43 (m, 2H); **^{13}C NMR** (75 MHz, $CDCl_3$) δ: 169.69, 151.38, 144.24, 129.58, 128.19, 127.04, 67.56, 60.87, 44.13, 33.52, 25.47; **HRMS** (*m/z*) calcd for $C_{27}H_{29}N_6O_4S^+$ [M + NH_4]$^+$ 533.1966, found 533.1947

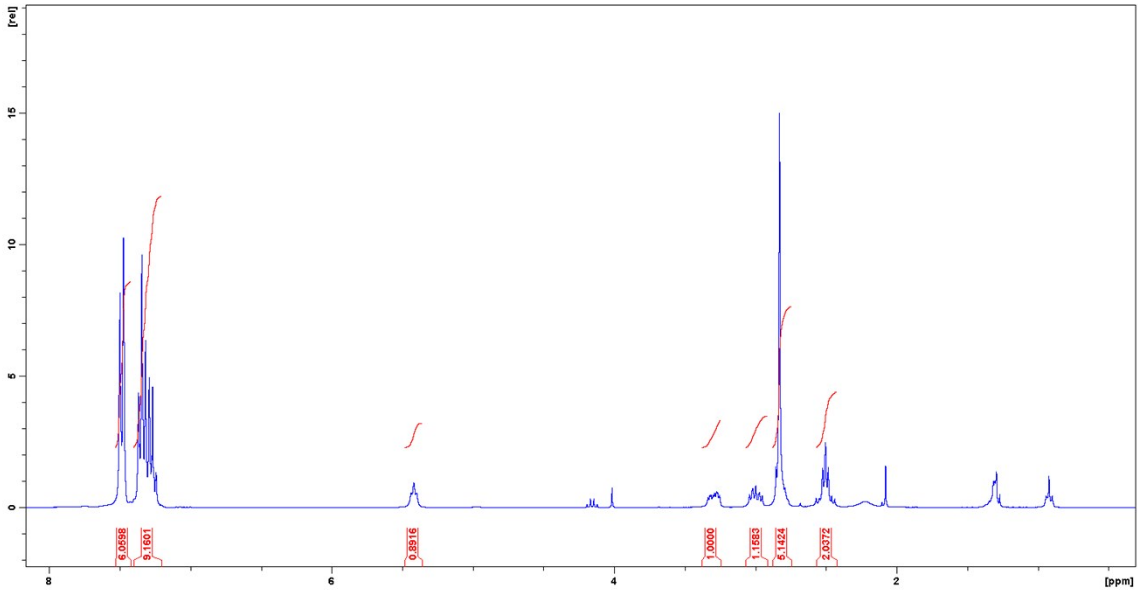

Figure S18 : ^{1}H NMR of monomer **M2** in $CDCl_3$ (300 MHz)

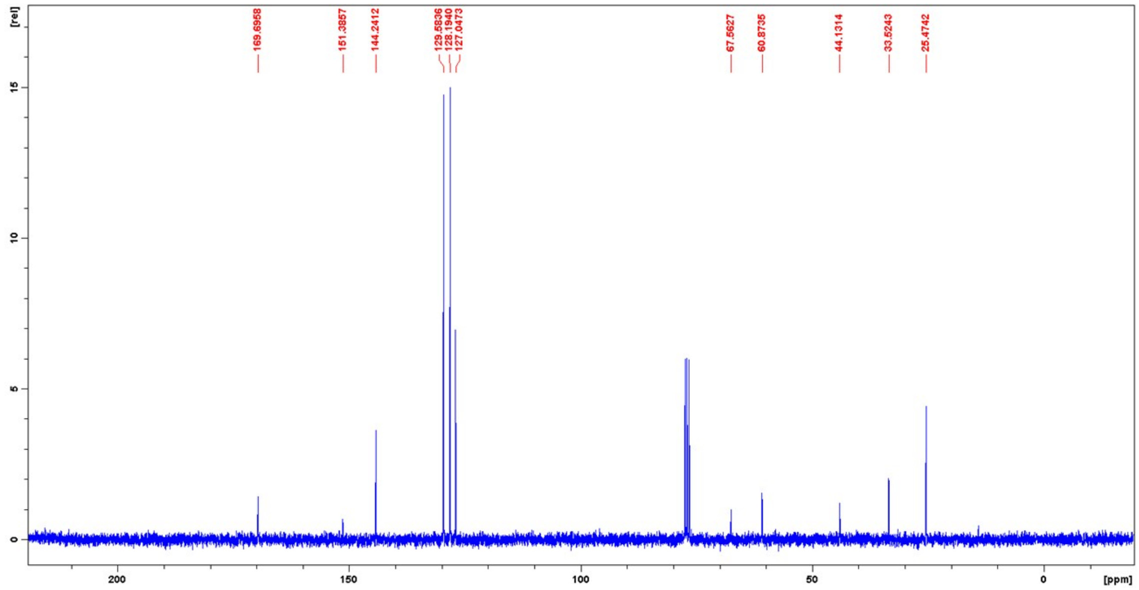

Figure S19 : ^{13}C NMR of monomer **M2** in $CDCl_3$ (75 MHz)

Monomer M3; 2,5-dioxopyrrolidin-1-yl (R)-(2-azido-5-oxo-5-(tritylamino)pentyl)carbamate; (N_3-Q[u])

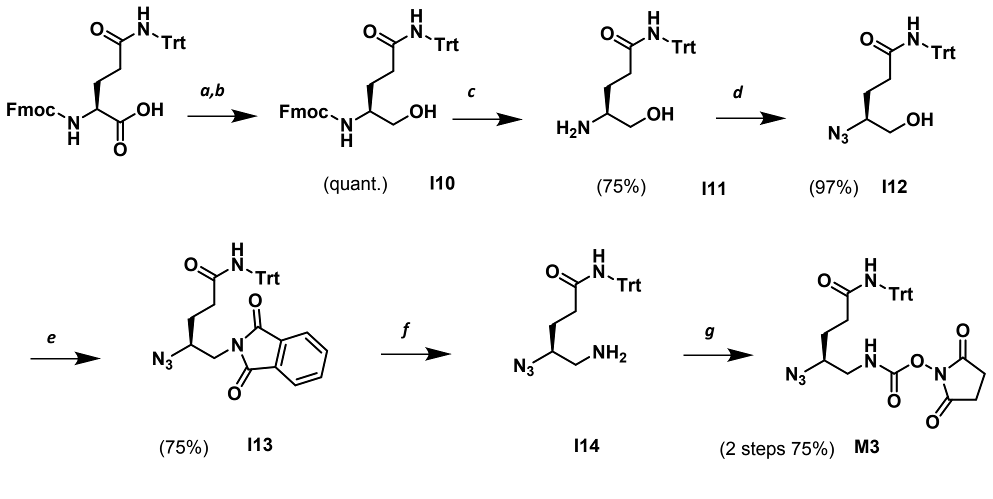

a: iBuOCOCl, NMM, THF, -10°C; ***b:*** $NaBH_4$, THF, H_2O, 0°C; ***c:*** piperidine, dioxane; ***d:*** K_2CO_3, $CuSO_4.5H_2O$, Imidazolium salt, CH_3CN/H_2O ***e:*** PPh_3, DIAD, Phtalimide, THF, 0°C; ; ***f:*** $N_2H_4.H_2O$, MeOH, 70°C, ***g:*** DSC, EtOAc, 0°C

Figure S20: Synthesis of monomer **M3**

a: Fmoc-L-Gln(Trt)-OH (12.6 g, 20.6 mmol) was dissolved in dry THF (in 500 mL double neck flask) at (-10°C, ice + salt bath) under N_2. 4-Methymorpholine (2.5 mL, 22.7 mmol) was added
Isobutyl chloroformate (2.8 mL, 21.6 mmol) was added dropwise via an addition funnel. The mixture was stirred 45 min at -10 °C.
The white precipitate was filtered and washed with THF.
b: Sodium borohydride (1.56 g, 41.3 mmol) was dissolved in 5 ml of H_2O and the previous filtrate was added dropwise at 0°C.
The mixture was stirred 3 h at RT.
THF was evaporated and the compound was solubilized in EtOAc and washed with $KHSO_4$ (1M), $NaHCO_3$ (sat), brine, dried over $MgSO_4$ and concentrated.
c: **I10** (14.4 g, 24.2 mmol) was dissolved in dioxane (80ml) and piperidine (20 ml) was added. The mixture was stirred 2 h at room temperature. The solvent was evaporated and the compound was purified by flash column chromatography on silica gel. Eluent DCM/MeOH ; gradient 100:0 to 80:20.
d: **I11** (5.80 g, 15.5 mmol) was dissolved in CH_3CN/H_2O (50:50, 100 ml) and MeOH (100 ml). Potassium carbonate (2.14 g, 15.49 mmol), Copper(II) sulfate pentahydrate (40.0 mg, 150 µmol) and imidazolium salt (3.90 g, 18.6 mmol) were added. The mixture was stirred for 2 h at RT. MeOH was evaporated. EtOAc was added and the phase were separated. Aqueous phase was extracted with EtOAc. Organic phases were combined and washed with $KHSO_4$ and brine, dried with $MgSO_4$ and concentrated.
e: **I12** (6.00 g, 14.98 mmol) was dissolved in anhydrous THF (50 ml) at 0 °C and triphenylphosphine (4.72 g, 17.98 mmol) and Phtalimide (2.65 g, 17.98 mmol) were added. DIAD (3.56 ml, 17.98 mmol) was added dropwise at 0 °C and the mixture was stirred 2 h at RT.
The solvent was evaporated and the compound was purified by flash column chromatography on silica gel. (liquid loading). Eluent Cyclohexane/EtOAc. Gradient 90:10 to 60:40.
f: **I13** (6.00 g, 11.3 mmol) was dissolved in MeOH (150 ml). Hydrazine monohydrated (1.65 mL, 33.9 mmol) was added and the mixture was stirred at 70 °C for 3 h. MeOH was evaporated. The compound was dissolved in EtOAc and washed with water and brine, dried with $MgSO_4$. The compound was used as is in the next step without evaporation.
g: N,N'-Disuccinimidyl carbonate (5.42 g, 21.1 mmol) was suspended in EtOAc (20 mL) at 0 °C. **I14** (21.1 mmol) previously dissolved in EtOAc (150 mL) was added dropwise at 0 °C and the mixture was stirred 2 h at room temperature.

The solvent was half evaporated. The organic phase was washed with $KHSO_4$ (1M), brine, dried over $MgSO_4$ and concentrated. The mixture was triturated in hexane to afford the monomer **M3** as a white powder (18.5g, 15.8 mmol, 77% yield).

Melting point (*M.p.*) 68.7 °C; **^{1}H NMR** ($CDCl_3$, 300 MHz) δ: 7.37-7.20 (m, 15H), 6.87 (s, 1H), 6.19 (m, 1H), 3.53 (m, 1H), 3.27-3.10 (m, 2H), 2.76 (s, 4H), 2.46 (m, 2H), 1.93-1.76 (m, 2H); **^{13}C NMR** (75 MHz, $CDCl_3$) δ: 170.99, 169.91, 151.88, 144.44, 128.63, 128.03, 127.13, 70.68, 60.63, 44.45, 32.45, 25.44; **HRMS** (*m/z*) calcd for $C_{29}H_{29}N_6O_5^+$ $[M + H]^+$ 541.2194, found 541.2208.

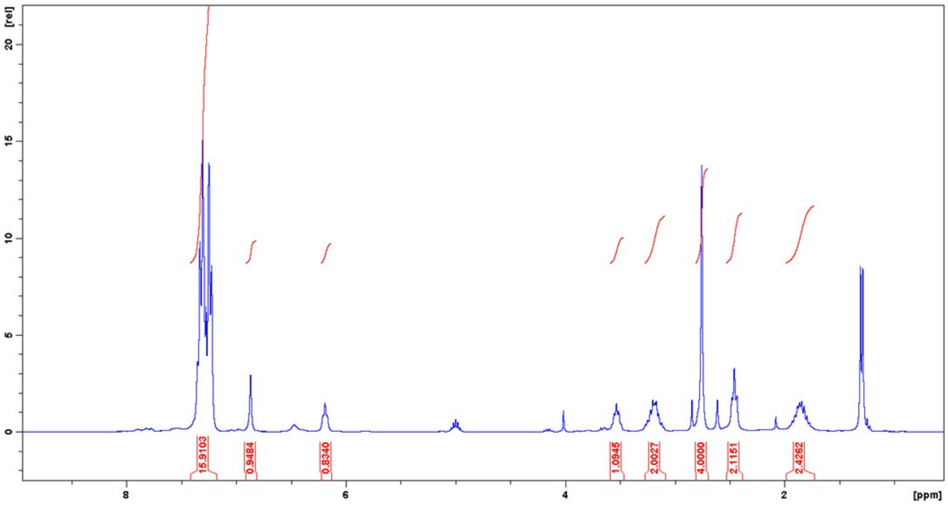

Figure S21 : ^{1}H NMR of monomer **M3** in $CDCl_3$ (300 MHz)

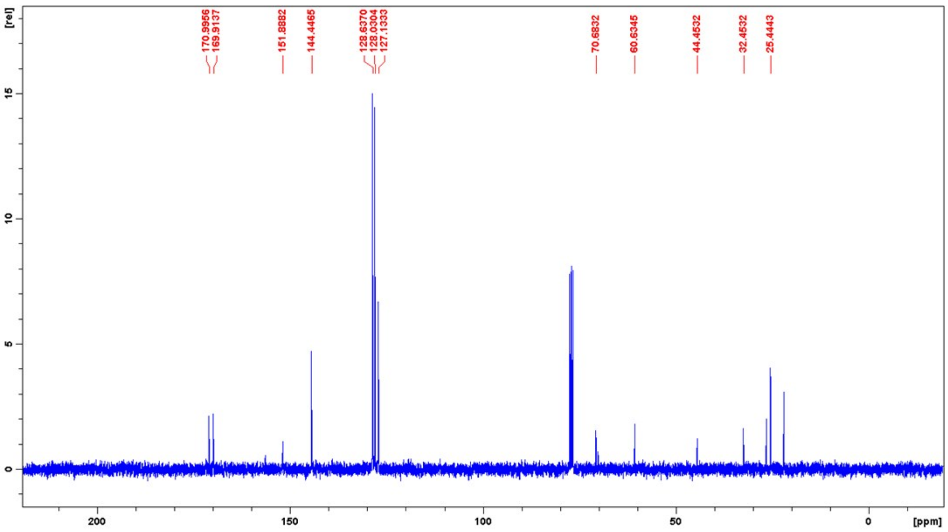

Figure S22 : ^{13}C NMR of monomer **M3** in $CDCl_3$ (75 MHz)

Monomer M4; 2,5-dioxopyrrolidin-1-yl ((2S,3R)-2-azido-3-(tert-butoxy)butyl)carbamate; (N_3-T[u])

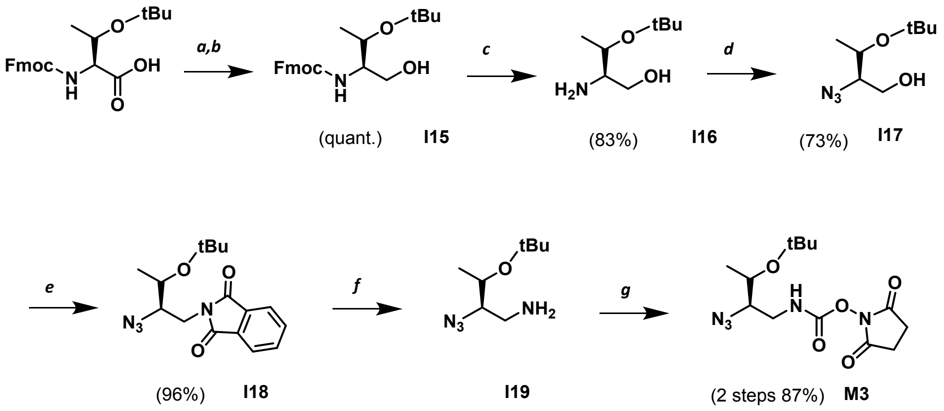

a: iBuOCOCl, NMM, THF, -10°C; ***b:*** $NaBH_4$, THF, H_2O, 0°C; ***c:*** piperidine, dioxane; ***d:*** K_2CO_3, $CuSO_4.5H_2O$, Imidazolium salt, CH_3CN/H_2O ***e:*** PPh_3, DIAD, Phtalimide, THF, 0°C; ; ***f:*** $N_2H_4.H_2O$, MeOH, 70°C, ***g:*** DSC, EtOAc, 0°C

Figure S23 : Synthesis of monomer **M4**

a: Fmoc-L-Thr(tBu)-OH (10.0 g, 25.2 mmol) was dissolved in dry THF (100 ml) at -10 °C, (ice + salt bath) under N_2 atmosphere. 4-Methymorpholine (3.0 mL, 27.7 mmol) was added. Isobutyl chloroformate (3.4 mL, 21.4 mmol) was added dropwise via an addition funnel. The mixture was stirred 45 min at -10 °C. The white precipitate was filtered and washed with THF.

b: Sodium borohydride (1.9 g, 50.3 mmol) was dissolved in 5 ml of H_2O and the previous filtrate was added dropwise at 0 °C. The mixture was stirred 3h at RT. THF was evaporated. The compound was solubilized in EtOAc and washed with $KHSO_4$ (1M), $NaHCO_3$ (sat) and brine (sat), dried with $MgSO_4$ and concentrated.

c: **I15** (9.5 g, 24.8 mmol) was dissolved in dioxane (80ml) and piperidine (20 ml) was added. The mixture was stirred 2h at room temperature. The solvent was evaporated and the compound was purified by flash column chromatography on silica gel. Eluent Cyclohexane/ EtOAc; gradient 50:50 to 0:100. And EtOAc/MeOH, 90:10.

d: **I16** (3.3 g, 20.5 mmol) was dissolved in CH_3CN/H_2O (50:50, 100 ml). Potassium carbonate (6.29 g, 45.5 mmol), Copper(II) sulfate pentahydrate (51.0 mg, 200 umol) and imidazolium salt (5.13 g, 24.6 mmol) were added. The mixture was stirred 2 h at RT. EtOAc was added and the phase were separated. Aqueous phase was extracted with EtOAc. Organic phases were combined and washed with $KHSO_4$ and brine, dried over $MgSO_4$ and concentrated. The solvent was evaporated and the compound was purified by flash column chromatography on silica gel. Eluent Cyclohexane/ EtOAc, 90:10.

e: **I17** (2.80 g, 15.0 mmol) was dissolved in anhydrous THF (50 ml) at 0 °C and triphenylphosphine (4.71 g, 17.9 mmol) and Phtalimide (2.64 g, 17.9 mmol) were added. DIAD (3.5 ml, 17.9 mmol) was added dropwise at 0 °C and the mixture was stirred 2 h at RT. The solvent was evaporated and the compound was purified by flash column chromatography on silica gel. (liquid loading). Eluent Cyclohexane/EtOAc. Gradient 90:10.

f: **I18** (4.56 g, 14.4 mmol) was dissolved in MeOH (50 ml). Hydrazine monohydrated (2.0 mL, 43.2 mmol) was added and the mixture was stirred at 70 °C for 2 h. The white precipitate was filtrated and washed with EtOAc. MeOH was evaporated. The compound was dissolved in EtOAc and washed with water and brine, dried with $MgSO_4$. The compound was used as is in the next step without evaporation.

g: N,N'-Disuccinimidyl carbonate (3.69 g, 14.4 mmol) was suspended in EtOAc (20 mL) at 0 °C. **I19** (14.4 mmol) previously dissolved in EtOAc (150 mL) was added dropwise at 0°C and the mixture was stirred 2 h at room temperature.

The solvent was half evaporated. The organic phase was washed with $KHSO_4$ (1M), brine, dried over $MgSO_4$ and concentrated. The mixture was triturated in hexane to afford the monomer **M4** as a white powder (4.1 g, 12.5 mmol, 50% yield).

Melting point (*M.p.*) 65.3 °C; **^{1}H NMR** ($CDCl_3$, 300 MHz) δ: 6.07 (m, 1H), 3.78 (m, 1H), 3.52-3.42 (m, 2H), 3.27 (m, 1H), 2.85 (s, 4H), 1.25 (s, 9H), 1.21 (d, *J* = 6.18 Hz, 3H); **^{13}C NMR** (75 MHz, $CDCl_3$) δ:169.92, 151.57, 74.93, 68.52, 65.47, 42.21, 28.29, 25.48, 19.37; **HRMS** (*m/z*) calcd for $C_{13}H_{21}N_5O_5Na^+$ $[M + Na]^+$ 350.1435, found 350.1438.

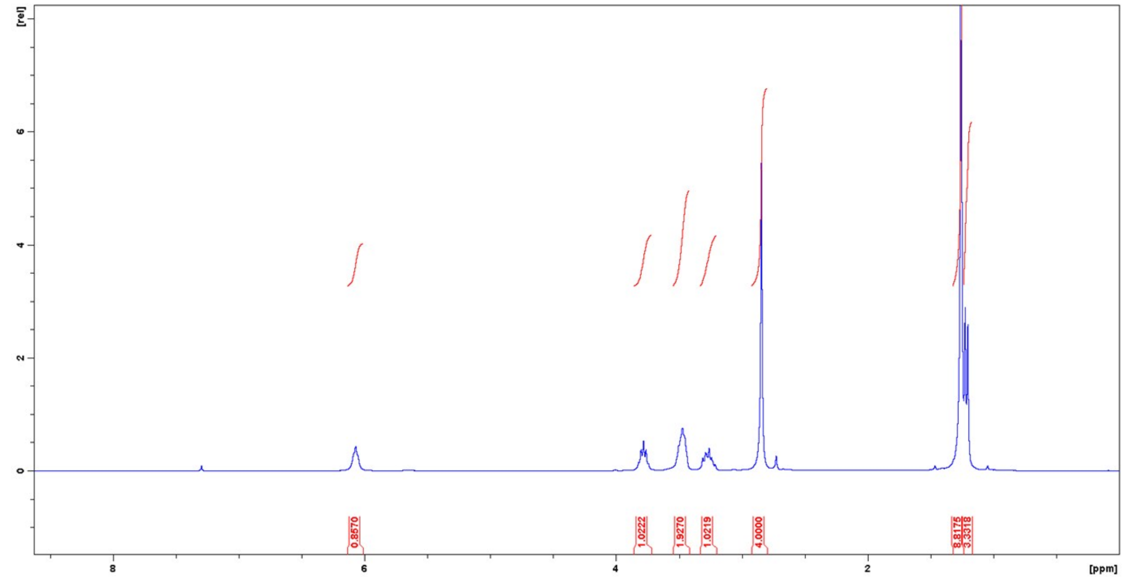

Figure S24 : ^{1}H NMR of monomer **M4** in $CDCl_3$ (300 MHz)

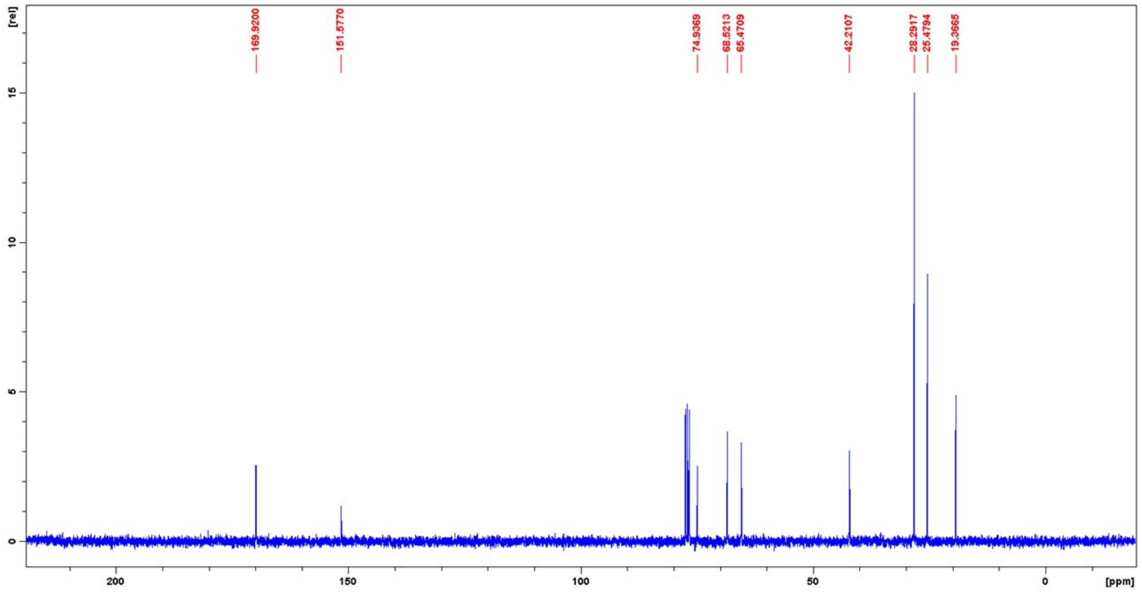

Figure S25 : ^{13}C NMR of monomer **M4** in $CDCl_3$ (75 MHz)

Monomer M5; 2,5-dioxopyrrolidin-1-yl (R)-(2-azido-5-(3-((2,2,4,6,7-pentamethyl-2,3-dihydrobenzofuran-5-yl)sulfonyl)guanidino)pentyl)carbamate; (N_3-R[u])

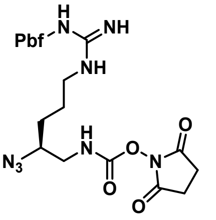

M5

Monomer M5 was synthesized by NovAliX.

Melting point (*M.p.*) 81.4 °C; **^{1}H NMR** ($CDCl_3$, 300 MHz) δ:6.83 (m, 1H), 6.45-6.35 (m, 2H), 3.66-3.56 (m, 1H), 3.39-3.30 (m, 2H), 3.30-3.19 (m, 2H), 2.98 (s, 2H), 2.84 (s, 4H), 2.66 (s, 1H), 2.58 (s, 3H), 2.51 (s, 3H), 2.11 (s, 3H), 1.73-1.53 (m, 4H), 1.48 (s, 6H); **^{13}C NMR** (75 MHz, $CDCl_3$) δ:170.6; 158.96; 156.22; 152.26; 138.43; 132.37; 124.86; 117.68; 86.61; 61.08; 44.85; 43.18; 28.61; 28.45; 25.55; 25.34; 19.30; 17.97; 12.49; **HRMS** (*m/z*) calcd for $C_{24}H_{35}N_8O_7S^+$ [M + H]$^+$ 579.2344, found 579.2341.

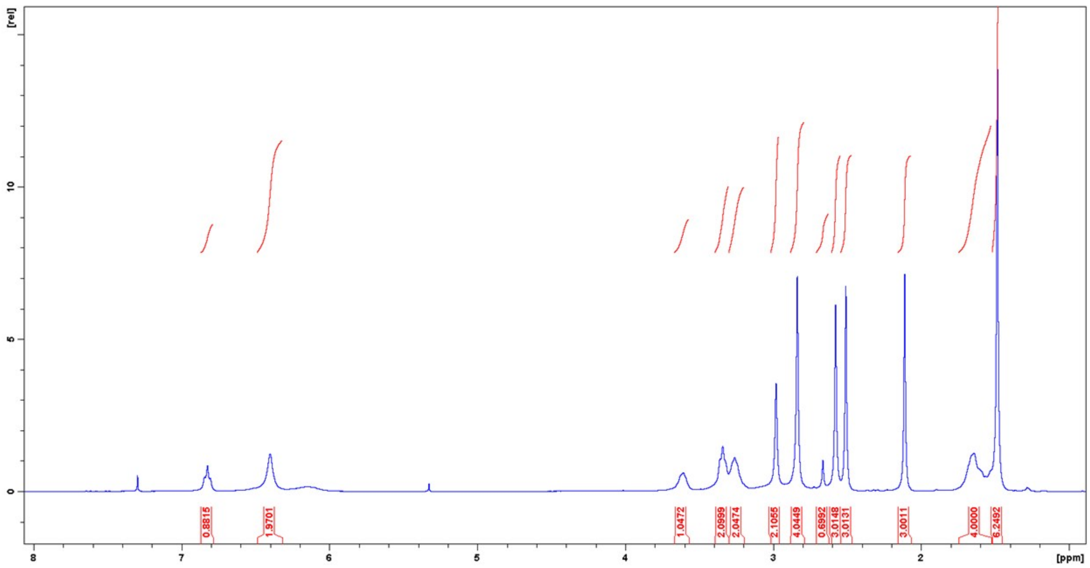

Figure S26 : ^{1}H NMR of monomer **M5** in $CDCl_3$ (300 MHz)

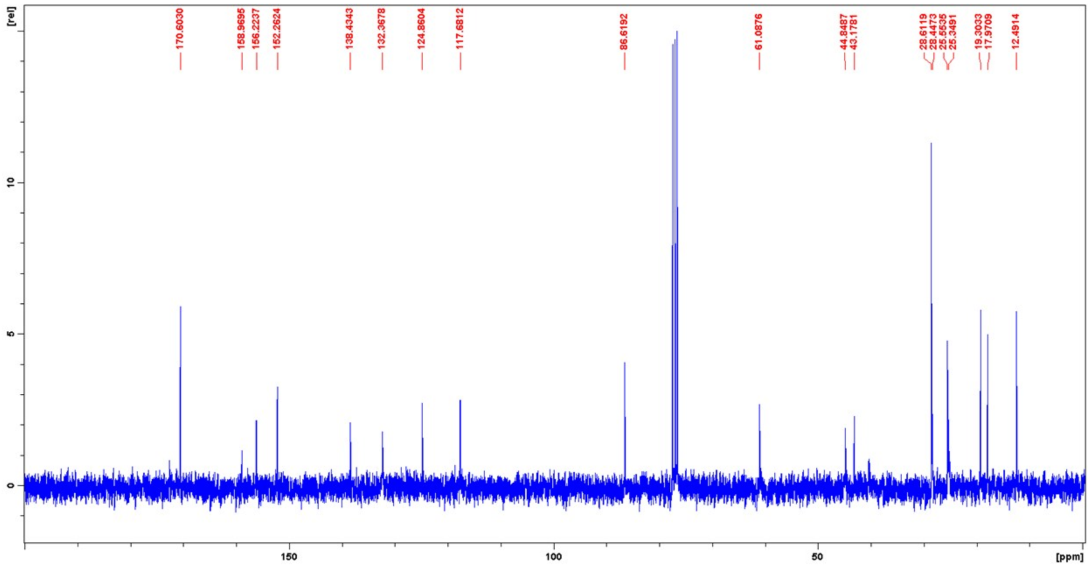

Figure S27 : ^{13}C NMR of monomer **M5** in $CDCl_3$ (75 MHz)

Monomer M6; (S)-2-azidopropyl (4-nitrophenyl) carbonate; (N_3-A^c)

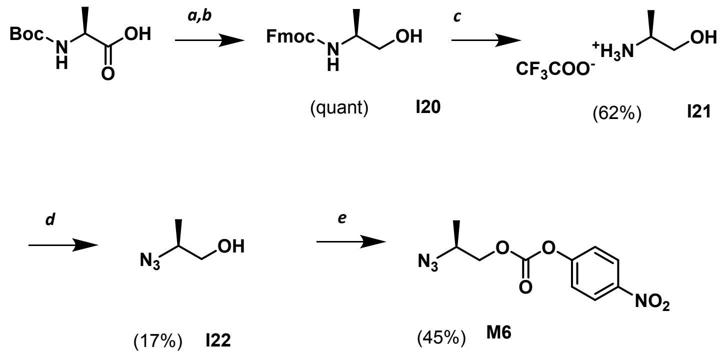

a: iBuOCOCl, NMM, THF, -10°C; ***b:*** $NaBH_4$, THF, H_2O, 0°C; ***c:*** TFA; ***d:*** K_2CO_3, $CuSO_4.5H_2O$, Imidazolium salt, CH_3CN/H_2O ***e:*** Pyridine, 4-nitrophenylchloroformate, DCM, N_2, 0°C;

Figure S28 : Synthesis of monomer **M6**

a: Boc-L-Ala-OH (10.0 g, 52.9 mmol) was dissolved in dry THF (100 ml) at -10 °C, (ice + salt bath) under N_2. 4-Methymorpholine (6.4 mL, 58.1 mmol) was added. Isobutyl chloroformate (7.2 mL, 55.5 mmol) was added dropwise via an addition funnel. The mixture was stirred 45 min at -10 °C. The white precipitate was filtered and washed with THF.
b: Sodium borohydride (4.0 g, 105.7 mmol) was dissolved in 8 ml of H_2O and the previous filtrate was added dropwise at 0 °C. The mixture was stirred overnight at room temperature. THF was evaporated. The compound was solubilized in EtOAc and washed with $KHSO_4$ (1M), $NaHCO_3$ (sat) and brine (sat), dried over $MgSO_4$ and concentrated.
c: **I20** (9.2 g, 52.9 mmol) was dissolved in TFA (40 ml). The mixture was stirred 3h at room temperature. TFA was evaporated and coevaporated with cyclohexane. The compound was dissolved in Et_2O and extracted with H_2O. The aqueous phase was lyophilized to afford **I21**.
d: **I21** (6.2 g, 32.7 mmol) was dissolved in CH_3CN/H_2O (50:50, 100 ml). Potassium carbonate (10.01 g, 72.4 mmol), Copper(II) sulfate pentahydrate (82.0 mg, 330 umol) and imidazolium salt (8.2 g, 39.2 mmol) were added. The mixture was stirred 1.5 h at room temperature. EtOAc was added and the phase were separated. Aqueous phase was extracted with EtOAc. Organic phases were combined and washed with $KHSO_4$ and brine. The solvent was evaporated and the compound was purified by flash column chromatography on silica gel. Eluent Cyclohexane/ EtOAc, 90:10.

e: **I22** (0.57 g, 5.6 mmol), was dissolved in DCM under N_2. Pyridine (0.5 ml, 6.2 mmol) was added and the reaction mixture was cooled to 0°C. 4-nitrophenylchloroformate (2.27 g, 11.3 mmol) dissolved in DCM (5ml) was added dropwise and the reaction mixture was stirred 5h. The organic phase was washed with $NaHCO_3$ (1M), dried over $MgSO_4$ and concentrated. The compound was purified by flash column chromatography on silica gel. Eluent Cyclohexane/ EtOAc, 90:10. Monomer **M6** (0.67 g, 2.5 mmol, 5% yield).

Melting point (*M.p.*) 35.3 °C; **^{1}H NMR** ($CDCl_3$, 300 MHz) δ: 8.35-8.29 (m, 2H),7.46-7.39 (m, 2H), 4.33 (dd, 1H, J= 10.89Hz, J = 4.26 Hz), 4.24 (dd, 1H, J= 11.45Hz, J= 7.01 Hz), 3.96-3.87 (m, 1H) 1.37 (d, 3H,J= 6.75 Hz) ; **^{13}C NMR** (75 MHz, $CDCl_3$) δ: 155.35, 152.26, 145.55, 125.38, 121.79, 71.42, 55.59, 15.81; **HRMS** (*m/z*) calcd for $C_{10}H_{11}N_4O_5^+$ [M + H]$^+$ 267.0724, found 267.0725.

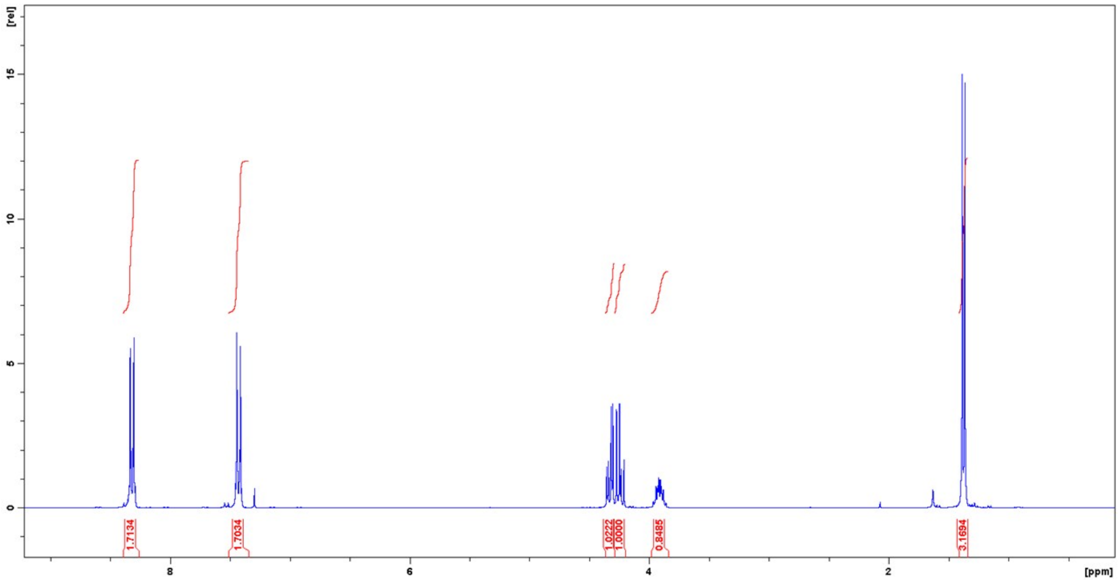

Figure S29 : ^{1}H NMR of monomer **M6** in $CDCl_3$ (300 MHz)

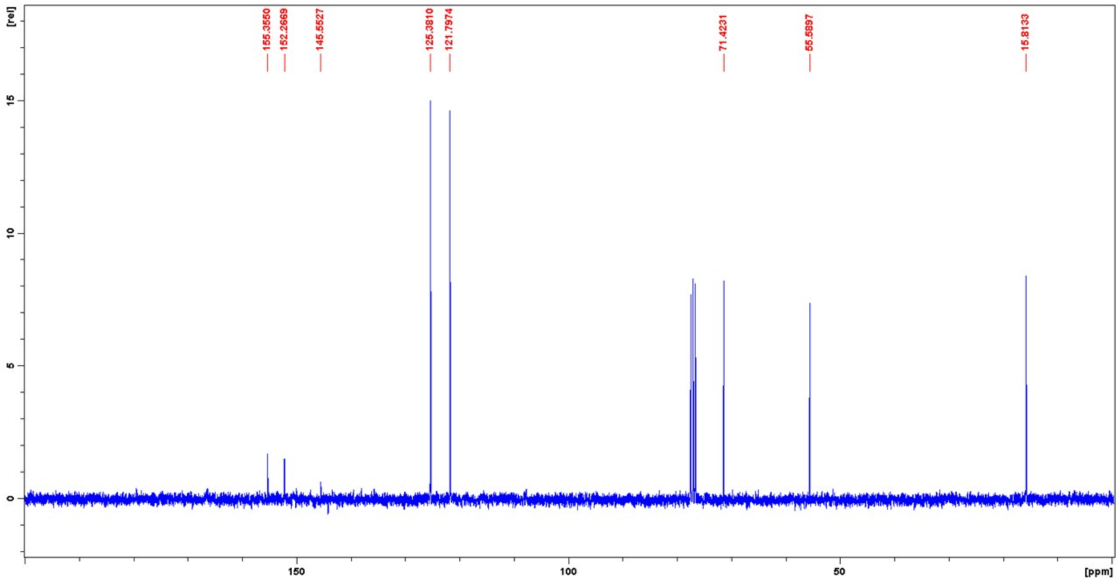

Figure S30 : ^{13}C NMR of monomer **M5** in $CDCl_3$ (75 MHz)

γ-Amino acid AA1; (S)-4-((((9H-fluoren-9-yl)methoxy)carbonyl)amino)pentanoic acid; (Fmoc-γA)

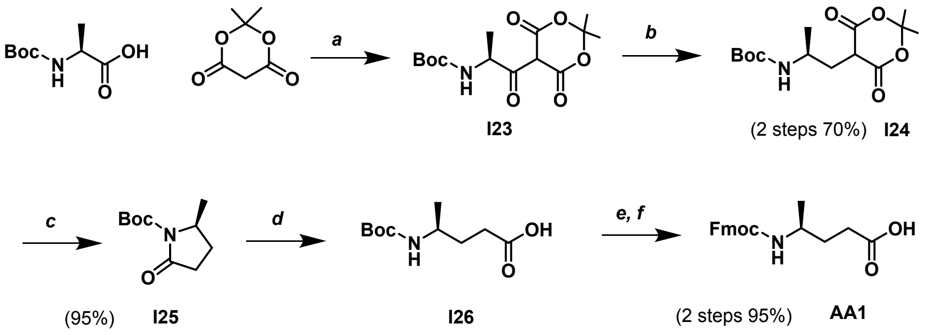

a: DCC, DMAP, DIEA, DCM, 0°C; ***b:*** Acetic acid, $NaBH_4$, DCM, 0°C; ***c:*** Toluène, 130°C; ***d:*** NaOH, Acetone;
e: TFA; ***f:*** $NaHCO_3$, Fmoc-OSu, Acetone/H_2O, 0°C

Figure S31 : Synthesis of Fmoc-γ-amino acid **AA1**

a: Meldrum's acid (9.14 g, 63.4 mmol) was dissolved in DCM (200 ml). Boc-L-Ala-OH (10.0 g, 52.9 mmol), DMAP (0.59 g, 5.28 mmol) and DIEA (13.93 ml, 81.9 mmol) were added at 0 °C. DCC (13.8 g, 63.4 mmol) dissolved in DCM (60 ml) was added dropwise to the previous mixture. The reaction mixture was stirred over night at 0 °C. The white solid was filtered and discarded. The organic phase was washed with $KHSO_4$ (1M) and brine (sat), dried with $MgSO_4$ and used as a solution in the next step
b: The previous solution of **I23** was cooled to 0°C and Acetic acid (30.2 ml, 528.7 mmol) was added. $NaBH_4$ (4.99 g, 132.2 mmol) was added portionwise and the mixture was stirred at room temperature overnight. Water was added. The organic phase was then washed with water and brine (sat), dried with $MgSO_4$ and concentrated. **I24** was recrystallized from DCM/Hexane.
c: **I24** (8.6 g, 27.3 mmol) was dissolved in toluene (200 ml) and stirred at 130°C for 4h. After completion, the toluene was evaporated.
d: **I25** (5.43g, 27.3 mmol) was dissolved in Acetone (40 ml) and NaOH (1M) (50 ml) was added, the mixture was stirred 1h at room temperature. After completion, acetone was evaporated. The aqueous phase was washed with DCM and then acidified with HCl (6N) (≈15 ml). The compound was extracted twice with DCM. Organic phases were combined, washed with brine, dried over $MgSO_4$ and concentrated.
e, f: **I26** (5.92 g, 27.2 mmol) was dissolved in TFA (40 ml) at 0 °C and stirred 40 min at room temperature. TFA was then evaporated and coevaporated with cyclohexane. The TFA mixture was dissolved in H2O/Acetone (160 ml, 50:50) and cooled to 0°C. $NaHCO_3$ (2.29 g, 27.2 mmol) was added portionwise until pH 9. Fmoc-OSu (10.0 g, 27.3 mmol) was added and the mixture was stirred overnight at room temperature. Acetone was evaporated. The aqueous phase was washed with Et_2O and then acidified with HCl (6N). The compound was extracted three times with DCM. Organic phases were combined, washed with brine, dried over $MgSO_4$ and concentrated. **AA1** was recrystallized from DCM/Hexane with a yield of 95%. (8.8 g, 26 mmol, 35% yield).

Melting point (*M.p.*) 124.5 °C; **^{1}H NMR** ($CDCl_3$, 300 MHz) δ: 7.79 (d, *J*= 7.44 Hz, 2H), 7.62 (d, *J*= 10.07 Hz, 2H), 7.46-7.29 (m, 4H), 5.75 (m, 1H), 4.73 (d, *J*= 8.46 Hz, 1H), 4.52-4.37 (m, 2H), 4.27-4.20 (m, 1H), 3.86-3.73 (m, 1H), 2.45-2.31; (m, 2H), 1.90-1.59 (m, 2H), 1.19 (d, *J*= 6.29 Hz, 3H) **^{13}C NMR** (75 MHz, $CDCl_3$) δ: 168.61, 143.95, 142.45, 141.33, 128.24, 127.46, 125.24, 120.19, 72.96, 47.34, 46.37, 31.86, 30.80, 24.57; **MS** (*m/z*) calcd for $C_{20}H_{22}NO_4^+$ $[M + H]^+$ 340.1543, found 340.2445.

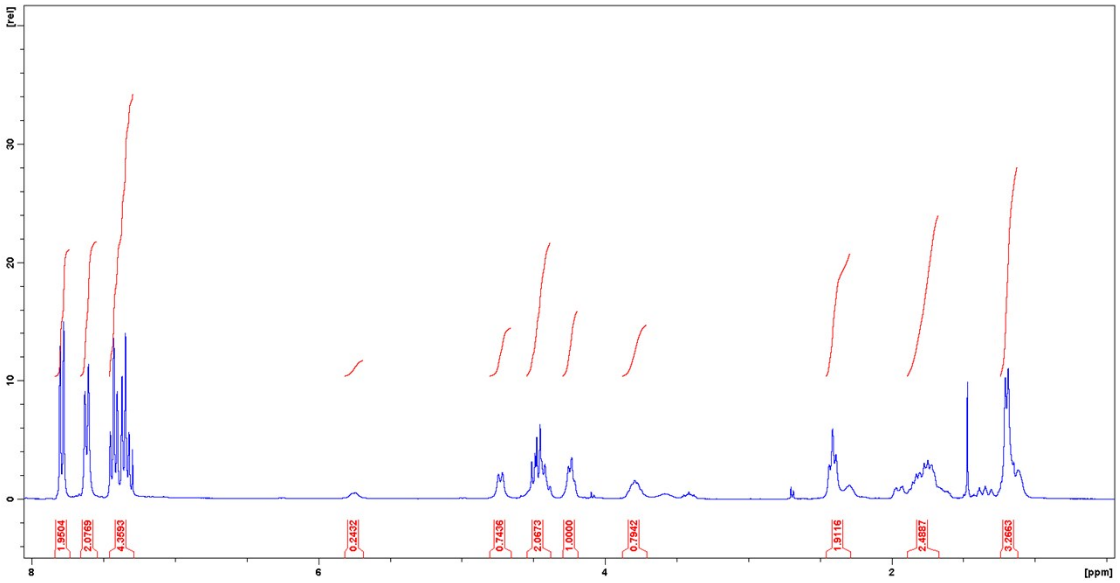

Figure S32 : ^{1}H NMR of γ-amino acid **AA1** in $CDCl_3$ (300 MHz)

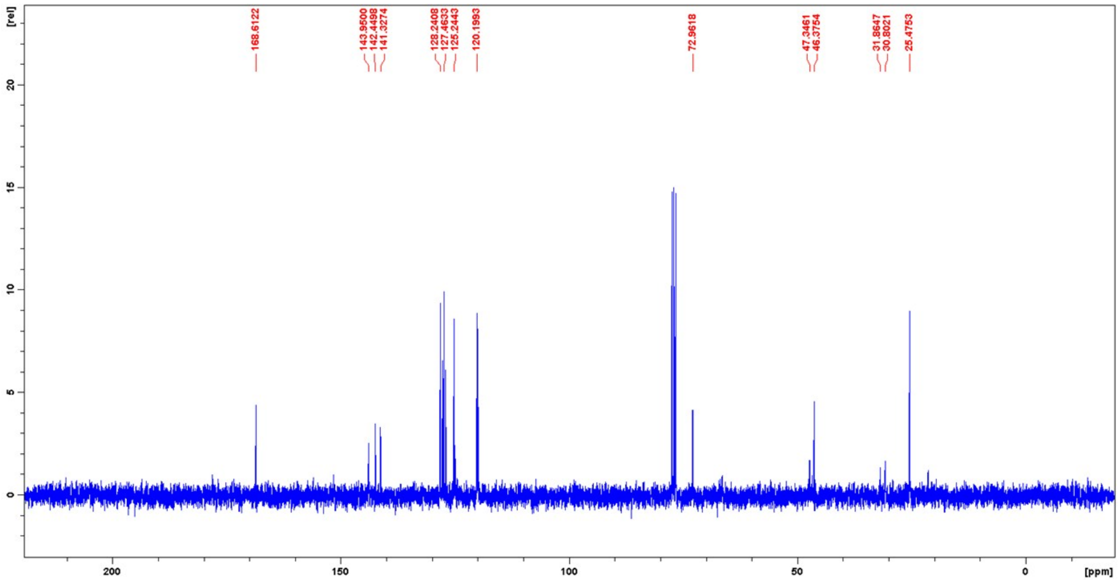

Figure S33 : ^{13}C NMR of γ-amino acid **AA1** in $CDCl_3$ (75 MHz).

Characterization of oligomers 2-15 and S1-S12 (analysis and biological activity)

H-HAEGTFTSDVSSYLEGQAAKEFIAWLVKGRG-NH$_2$ (2, URK-062)

Peptide **2** has been synthesized using the general procedure **P1** starting from sieber resin (160 mg, 0.1 mmol). The final product **2** was purified by semi-preparative HPLC. 40 mg was obtained (yield 12 %). **HPLC**: R_t= 5.19 min (10-100% CH_3CN 0.1 % TFA in H_2O 0.1% TFA, 10 min, C18); **ESI-MS** *(m/z 3354.73)*: 672.00 $[M+5H]^{5+}$, 839.80 $[M+4H]^{4+}$, 1119.53 $[M+3H]^{3+}$

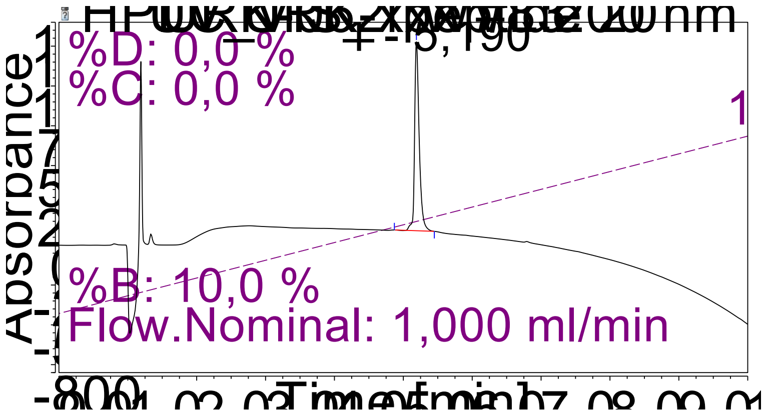

Figure S34: HPLC profile of peptide **2** (Gradient: 10-100%; CH_3CN 0.1 % TFA in H_2O 0.1% TFA, 10 min, C18).

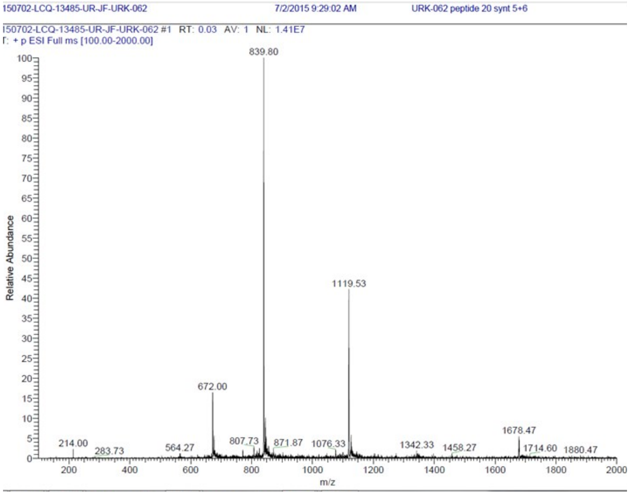

Figure S35 : electrospray ionization spectrum of peptide **2** performed on a DTIMS-Q-TOF spectrometer, with the dual-ESI source operated in positive ion mode.

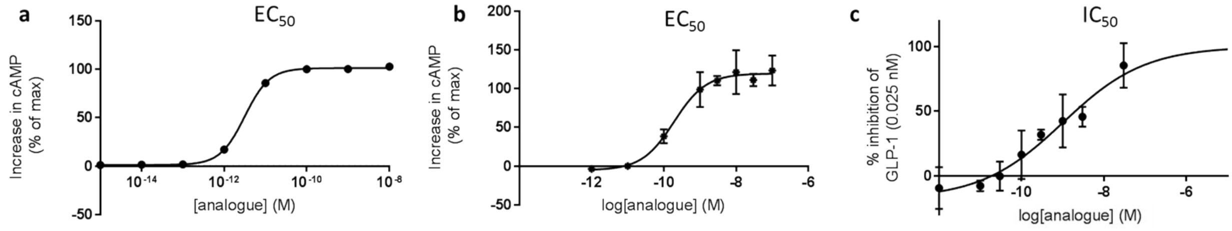

Figure S36 : (a-b) Concentration-response curve (receptor-mediated cAMP produced) for EC_{50} determination of **2** in cells expressing the GLP-1R (a) Method C1 (b) Method C2. (c) Concentration-inhibition curve of **2** in a radioligand ([^{125}I]GLP-1(7-36)) binding assay on cell membrane homogenates for affinity (IC_{50}) to GLP-1R determination.

H-HA^{u}EGTFTSDVSSYLEGQAAKEFIAWLVKGRG-NH$_2$ (3, URK-286)

Oligomer **3** has been synthesized using the general procedure **P1** starting from sieber resin (160 mg, 0.1 mmol). The final product **3** was purified by semi-preparative HPLC. 4.6 mg was obtained (yield 1.3 %). **HPLC**: R_t= 5.22 min (10-100% CH_3CN 0.1 % TFA in H_2O 0.1% TFA, 10 min, C18); **ESI-MS** *(m/z 3383.78)*: 677.60 $[M+5H]^{5+}$, 847.20 $[M+4H]^{4+}$, 1129.00 $[M+3H]^{3+}$

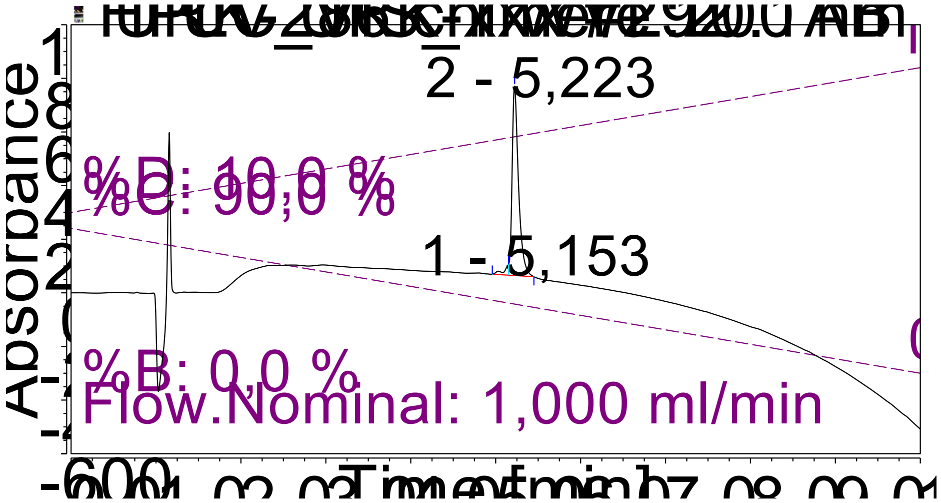

Figure S37 : HPLC profile of oligomer **3** (Gradient: 10-100%; CH_3CN 0.1 % TFA in H_2O 0.1% TFA, 10 min, C18).

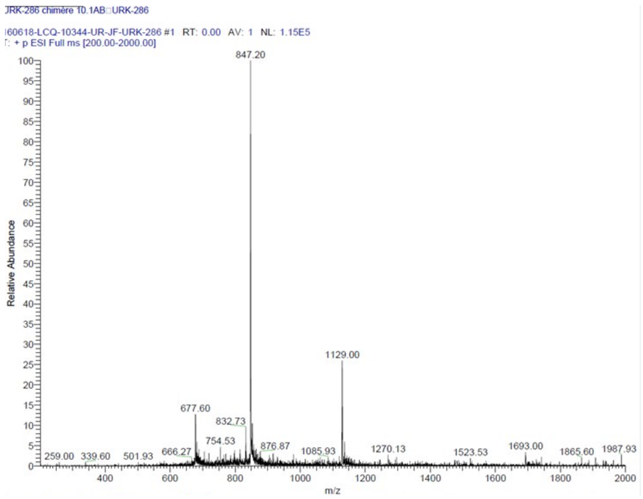

Figure S38 : electrospray ionization spectrum of peptide **3** performed on a DTIMS-Q-TOF spectrometer, with the dual-ESI source operated in positive ion mode.

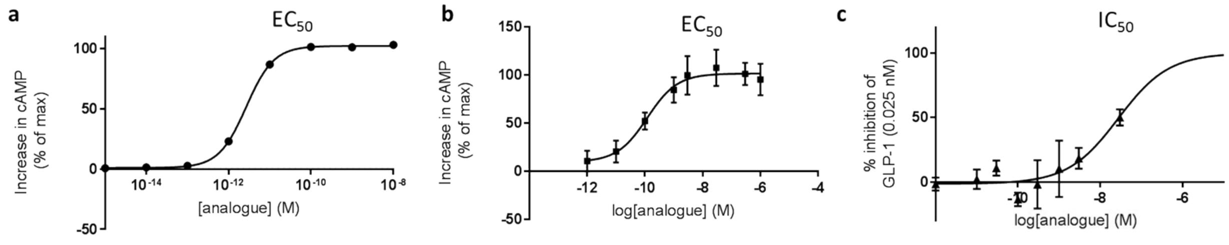

Figure S39 : (a-b) Concentration-response curve (receptor-mediated cAMP produced) for EC_{50} determination of **3** in cells expressing the GLP-1R (a) Method C1 (b) Method C2. (c) Concentration-inhibition curve of **3** in a radioligand ([^{125}I]GLP-1(7-36)) binding assay on cell membrane homogenates for affinity (IC_{50}) to GLP-1R determination.

H-HγAEGTFTSDVSSYLEGQAAKEFIAWLVKGRG-NH_2 (4, URK-459)

Peptide **4** has been synthesized using the general procedure **P1** starting from sieber resin (160 mg, 0.1 mmol). The final product **4** was purified by semi-preparative HPLC. 2.5 mg was obtained (yield 0.8 %). **HPLC**: R_t= 5.15 min (10-100%; CH_3CN 0.1 % TFA in H_2O 0.1% TFA, 10 min, C18); **LC-MS** *(m/z 3382.79)*: 677.49 $[M+5H]^{5+}$, 846.85 $[M+4H]^{4+}$, 1128.77 $[M+3H]^{3+}$, 1692.61 $[M+2H]^{2+}$

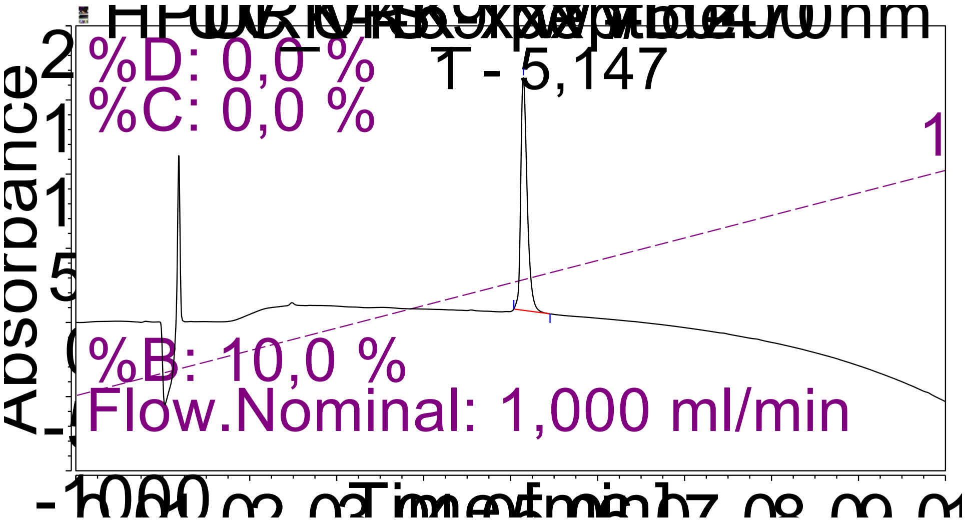

Figure S40 : HPLC profile of oligomer **4** (Gradient: 10-100%; CH_3CN 0.1 % TFA in H_2O 0.1% TFA, 10 min, C18).

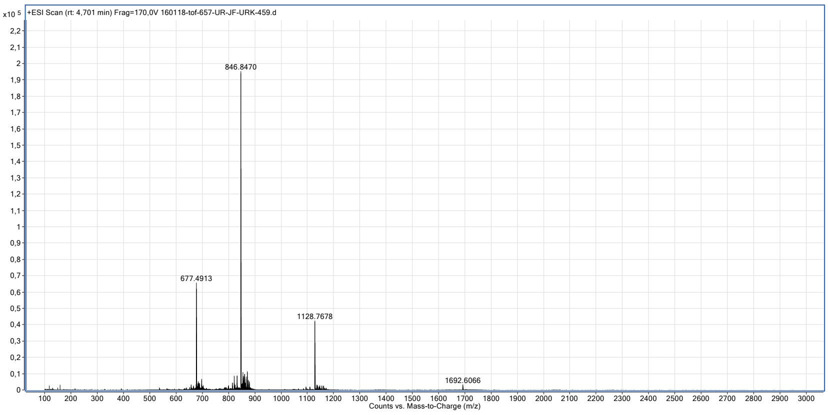

Figure S41 : LC-MS spectrum of oligomer **4** performed on UHPLC coupled to an ESI -MS Tof with the dual-ESI source operated in positive ion mode. (Gradient: 10-100%; CH_3CN 0.1 % Formic acid in H_2O 0.1% formic acid, 6 min, C18).

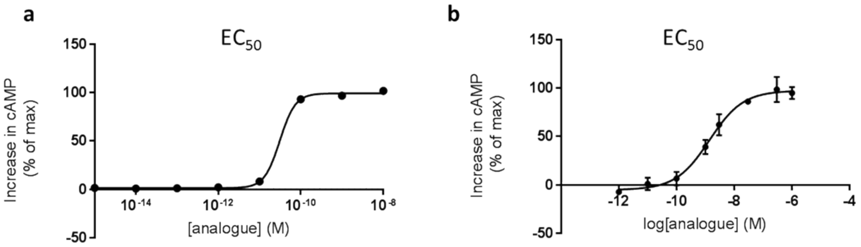

Figure S42 : Concentration-response curve (receptor-mediated cAMP produced) for EC_{50} determination of **4** in cells expressing the GLP-1R (a) Method C1 (b) Method C2.

H-HA^{c}EGTFTSDVSSYLEGQAAKEFIAWLVKGRG-NH_2 (5, URK-565)

Oligomer **5** has been synthesized using the general procedure **P1** starting from sieber resin (160 mg, 0.1 mmol). The final product **5** was purified by semi-preparative HPLC. 2.75 mg was obtained (yield 0.8 %). **HPLC**: R_t= 5.19 min (10-100%; CH_3CN 0.1 % TFA in H_2O 0.1% TFA, 10 min, C18); **LC-MS** *(m/z 3384.76)*: 677.95 $[M+5H]^{5+}$, 846.93 $[M+4H]^{4+}$, 1128.90 $[M+3H]^{3+}$, 1693.35 $[M+2H]^{2+}$

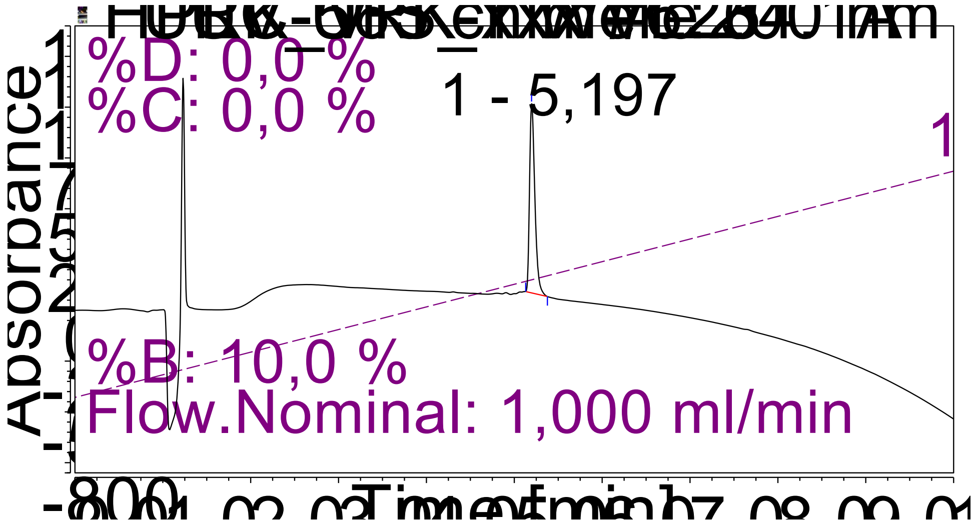

Figure S43 : HPLC profile of oligomer **5** (Gradient: 10-100%; CH_3CN 0.1 % TFA in H_2O 0.1% TFA, 10 min, C18).

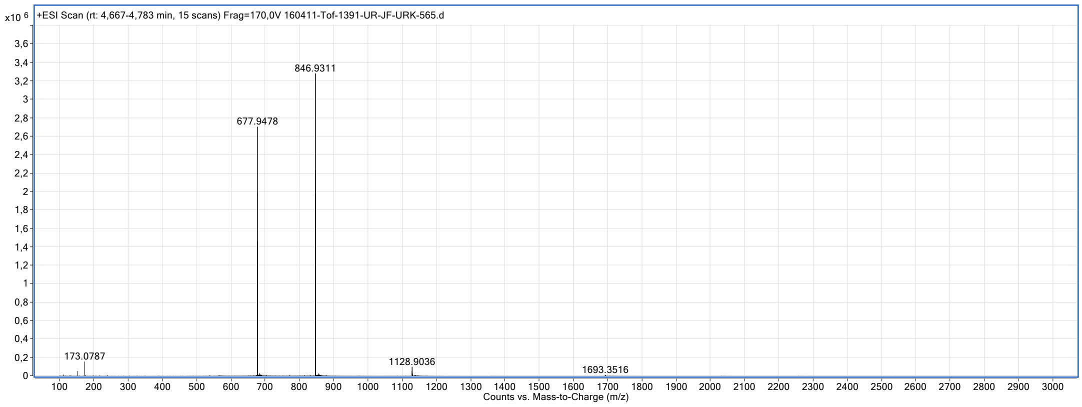

Figure S44 : LC-MS spectrum of oligomer **5** performed on UHPLC coupled to an ESI -MS Tof with the dual-ESI source operated in positive ion mode. (Gradient: 10-100%; CH_3CN 0.1 % Formic acid in H_2O 0.1% formic acid, 6 min, C18).

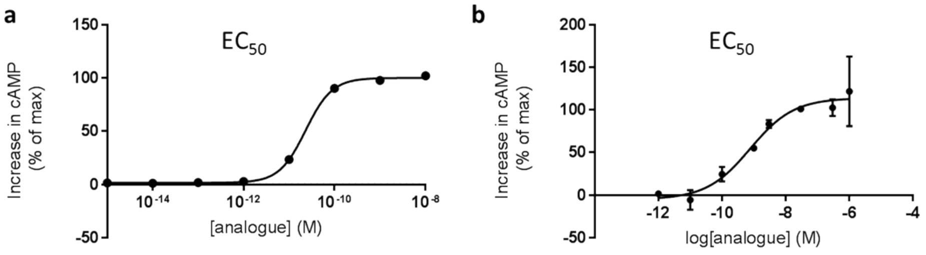

Figure S45 : Concentration-response curve (receptor-mediated cAMP produced) for EC_{50} determination of **5** in cells expressing the GLP-1R (a) Method C1 (b) Method C2.

H-HA$^{u\alpha}$EGTFTSDVSSYLEGQAAKEFIAWLVKGRG-NH$_2$ (6, URK-573)

Oligomer **6** has been synthesized using the general procedure **P1** starting from sieber resin (160 mg, 0.1 mmol). The final product **6** was purified by semi-preparative HPLC. 1.2 mg was obtained (yield 0.3 %). **HPLC**: R_t= 5.20 min (10-100% CH_3CN 0.1 % TFA in H_2O 0.1% TFA, 10 min, C18); **LC-MS** *(m/z 3383.78)*: 677.37 $[M+5H]^{5+}$, 846.49 $[M+4H]^{4+}$, 1128.35 $[M+3H]^{3+}$

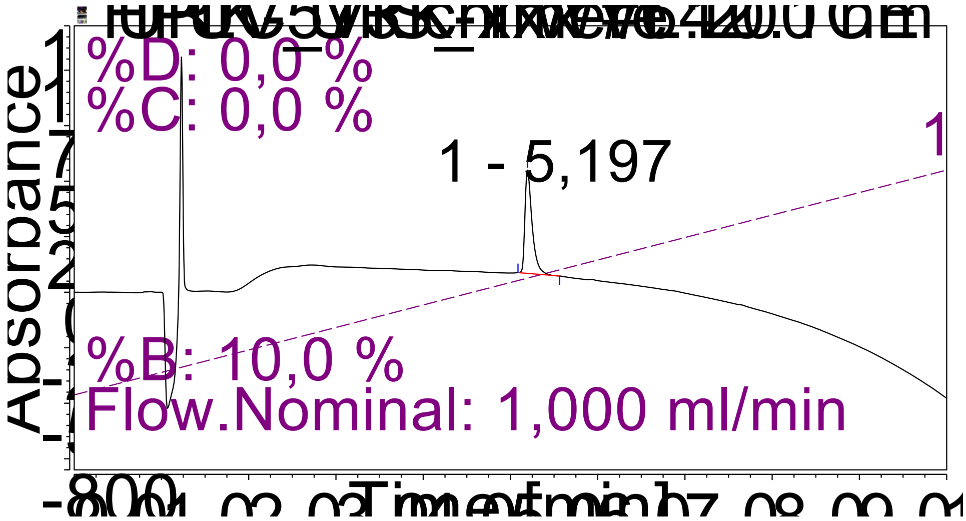

Figure S46 : HPLC profile of oligomer **6** (Gradient: 10-100%; CH_3CN 0.1 % TFA in H_2O 0.1% TFA, 10 min, C18).

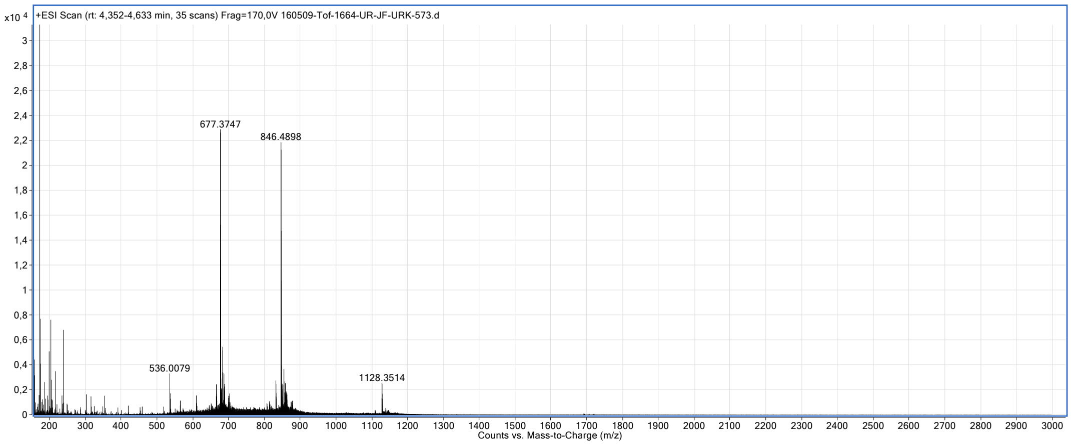

Figure S47 : LC-MS spectrum of oligomer **6** performed on UHPLC coupled to an ESI -MS Tof with the dual-ESI source operated in positive ion mode. (Gradient: 10-100%; CH_3CN 0.1 % Formic acid in H_2O 0.1% formic acid, 6 min, C18).

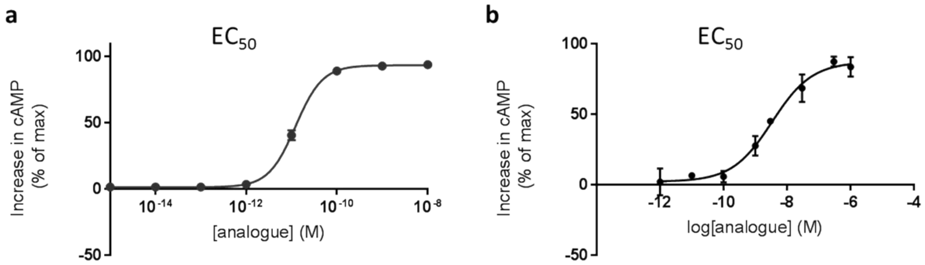

Figure S48 : Concentration-response curve (receptor-mediated cAMP produced) for EC_{50} determination of **6** in cells expressing the GLP-1R (a) Method C1 (b) Method C2.

H-HG^{u}EGTFTSDVSSYLEGQAAKEFIAWLVKGRG-NH$_2$ (7, URK-521)

Oligomer **7** has been synthesized using the general procedure **P1** starting from sieber resin (160 mg, 0.1 mmol). The final product **7** was purified by semi-preparative HPLC. 2.4 mg was obtained (yield 0.7 %). **HPLC**: R_t= 5.19 min (10-100% CH_3CN 0.1 % TFA in H_2O 0.1% TFA, 10 min, C18); **LC-MS** *(m/z 3369.75)*: 674.94 $[M+5H]^{5+}$, 843.42 $[M+4H]^{4+}$, 1123.89 $[M+3H]^{3+}$

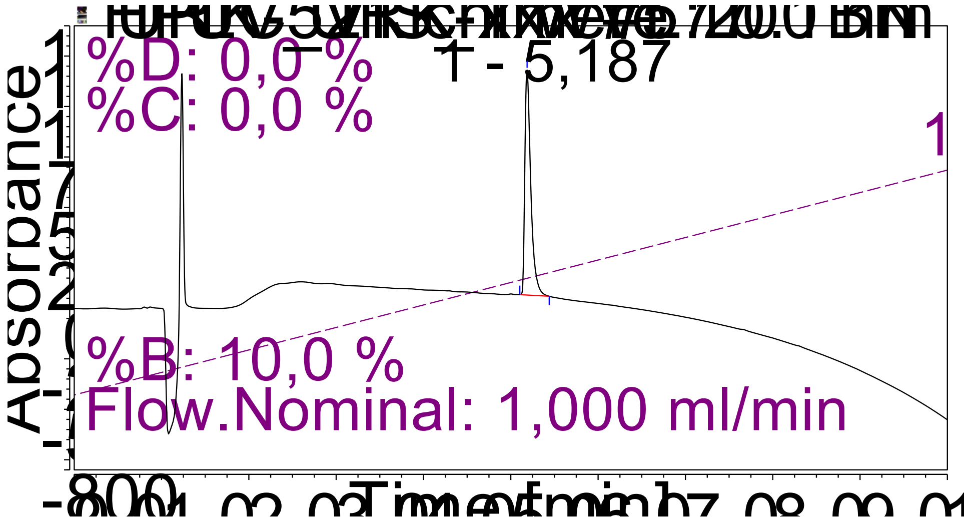

Figure S49 : HPLC profile of oligomer **7** (Gradient: 10-100%; CH_3CN 0.1 % TFA in H_2O 0.1% TFA, 10 min, C18).

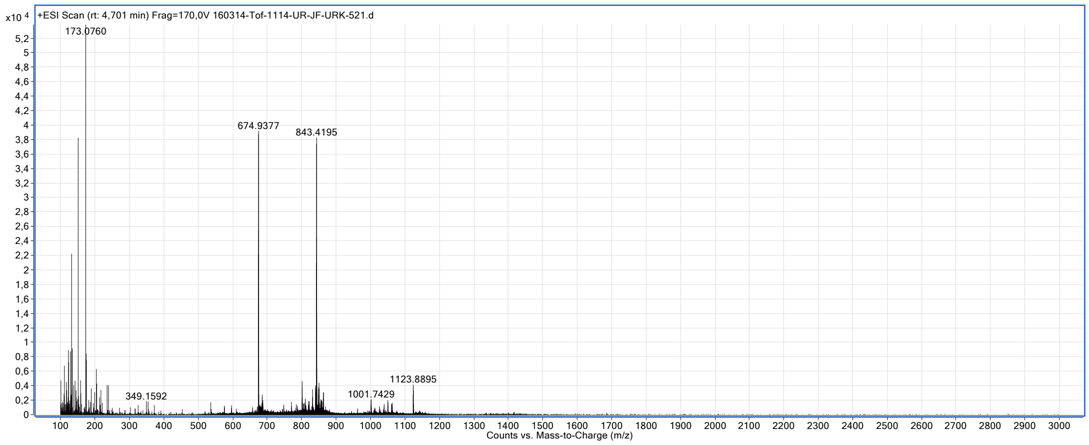

Figure S50 : LC-MS spectrum of oligomer **7** performed on UHPLC coupled to an ESI -MS Tof with the dual-ESI source operated in positive ion mode. (Gradient: 10-100%; CH_3CN 0.1 % Formic acid in H_2O 0.1% formic acid, 6 min, C18).

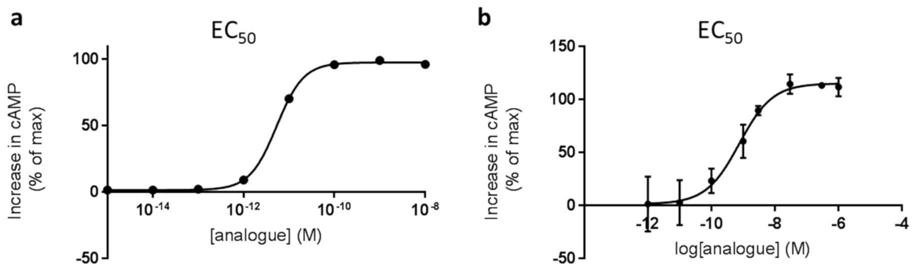

Figure S51 : Concentration-response curve (receptor-mediated cAMP produced) for EC_{50} determination of **7** in cells expressing the GLP-1R (a) Method C1 (b) Method C2.

H-HV^{u}EGTFTSDVSSYLEGQAAKEFIAWLVKGRG-NH$_2$ (8, URK-564)

Oligomer **8** has been synthesized using the general procedure **P1** starting from sieber resin (160 mg, 0.1 mmol). The final product **8** was purified by semi-preparative HPLC. 2.7 mg was obtained (yield 0.8 %). **HPLC**: R_t= 5.23 min (10-100% CH_3CN 0.1 % TFA in H_2O 0.1% TFA, 10 min, C18); **LC-MS** *(m/z 3411.83)*: 683.15 $[M+5H]^{5+}$, 853.69 $[M+4H]^{4+}$, 1138.25 $[M+3H]^{3+}$, 1706.87 $[M+2H]^{2+}$

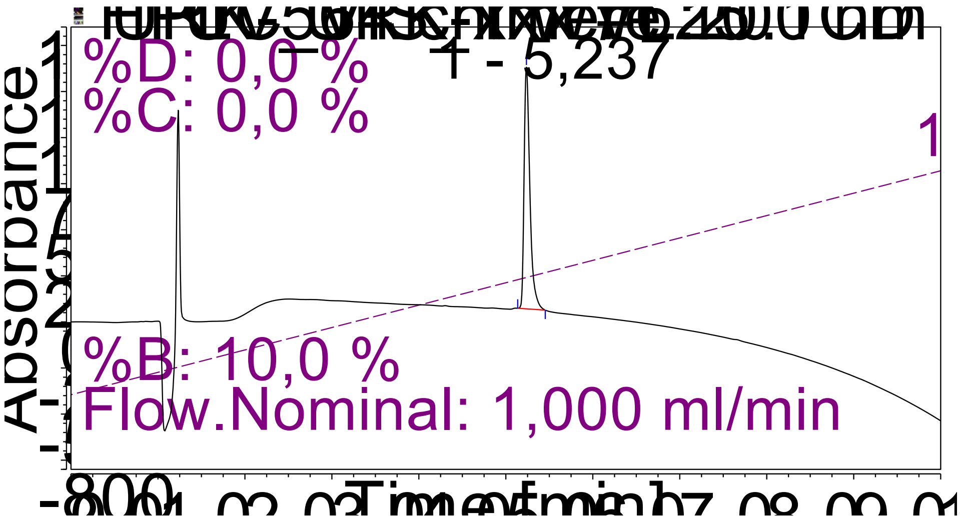

Figure S52 : HPLC profile of oligomer **8** (Gradient: 10-100%; CH_3CN 0.1 % TFA in H_2O 0.1% TFA, 10 min, C18).

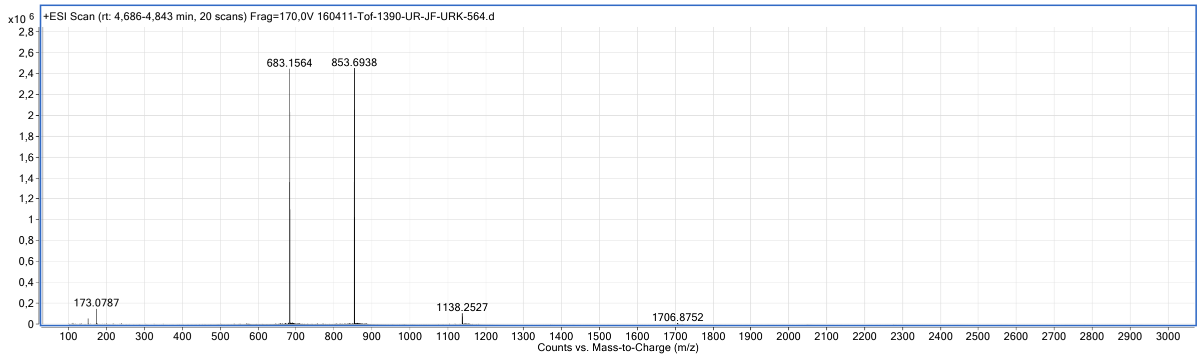

Figure S53 : LC-MS spectrum of oligomer **8** performed on UHPLC coupled to an ESI -MS Tof with the dual-ESI source operated in positive ion mode. (Gradient: 10-100%; CH_3CN 0.1 % Formic acid in H_2O 0.1% formic acid, 6 min, C18).

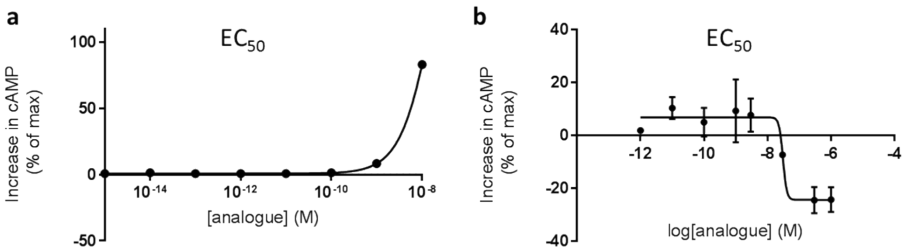

Figure S54 : Concentration-response curve (receptor-mediated cAMP produced) for EC_{50} determination of **8** in cells expressing the GLP-1R (a) Method C1 (b) Method C2.

H-HAEGTFTSDLSKQMEEEAVKLFIEWLKNGGPSSGAPPPS-NH$_2$ (9, URK-075)

Peptide **9** has been synthesized using the general procedure **P1** starting from sieber resin (160 mg, 0.1 mmol). The final product **9** was purified by semi-preparative HPLC. 23 mg was obtained (yield 5.5 %). **HPLC**: R_t= 5.19 min (10-100% CH_3CN 0.1 % TFA in H_2O 0.1% TFA, 10 min, C18); **ESI-MS** *(m/z 4186.63)*: 842.88 $[M+5H]^{5+}$, 1047.65 $[M+4H]^{4+}$, 1396.23 $[M+3H]^{3+}$

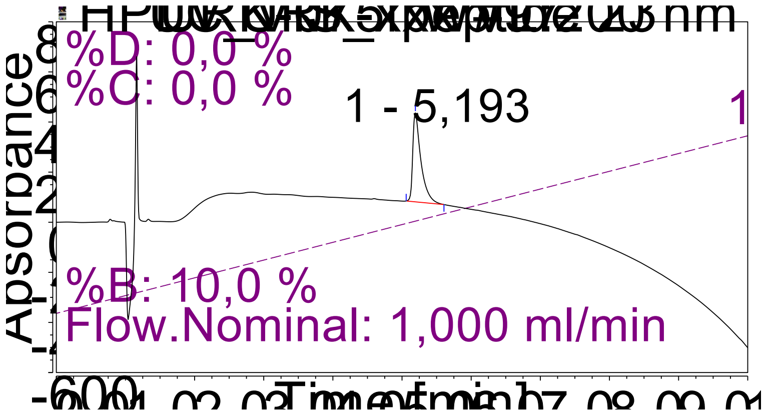

Figure S55 : HPLC profile of peptide **9** (Gradient: 10-100%; CH_3CN 0.1 % TFA in H_2O 0.1% TFA, 10 min, C18).

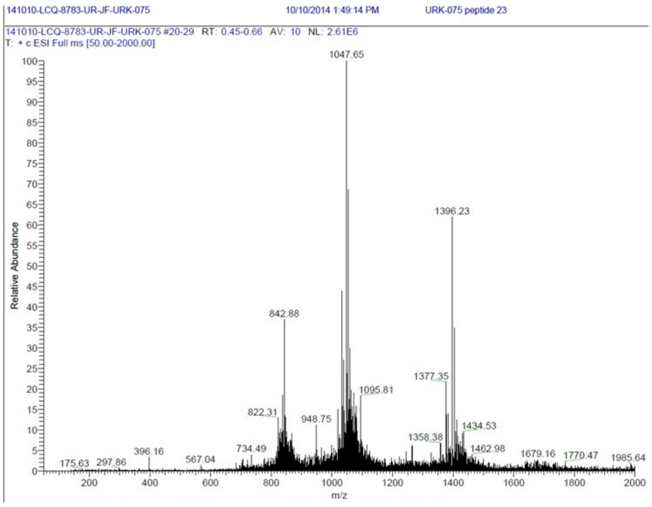

Figure S56 : electrospray ionization spectrum of peptide **9** performed on a DTIMS-Q-TOF spectrometer, with the dual-ESI source operated in positive ion mode.

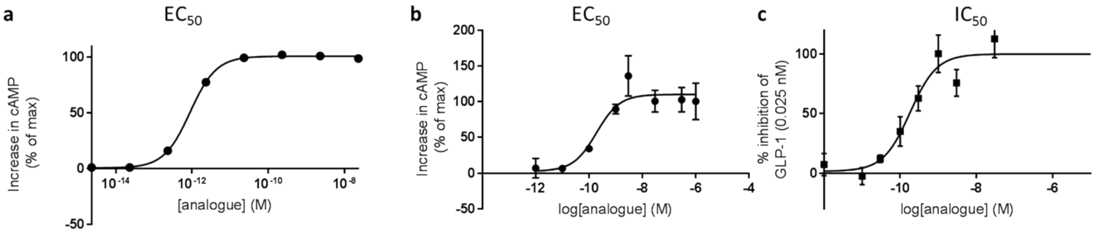

Figure S57 : Concentration-response curve (receptor-mediated cAMP produced) for EC_{50} determination of **9** in cells expressing the GLP-1R (a) Method C1 (b) Method C2. (c) Concentration-inhibition curve of **9** in a radioligand ($[^{125}I]$GLP-1(7-36)) binding assay on cell membrane homogenates for affinity (IC_{50}) to GLP-1R determination.

H-HAEGTFTSDLSKQLEEEAVKLFIEWLKNGGPSSGAPPPS-NH_2 (10, URK-200)

Peptide **10** has been synthesized using the general procedure **P1** starting from sieber resin (160 mg, 0.1 mmol). The final product **10** was purified by semi-preparative HPLC. 11.8 mg was obtained (yield 2.8 %). **HPLC**: R_t= 5.32 min (10-100% CH_3CN 0.1 % TFA in H_2O 0.1% TFA, 10 min, C18); **ESI-MS** *(m/z 4168.60)*: 834.62 $[M+5H]^{5+}$, 1043.02 $[M+4H]^{4+}$, 1390.36 $[M+3H]^{3+}$

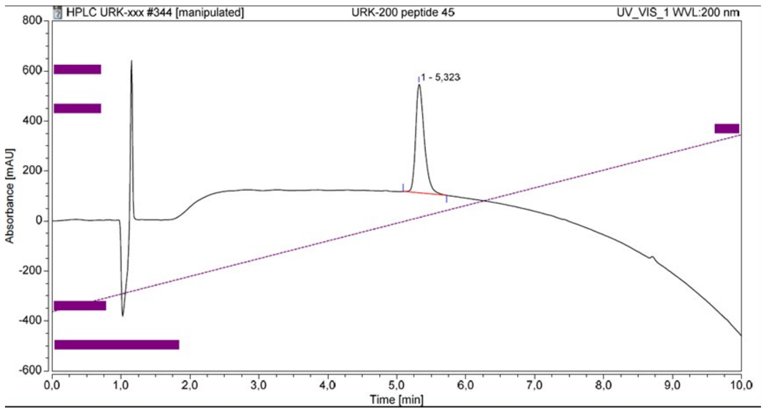

Figure S58 : HPLC profile of peptide **10** (Gradient: 10-100%; CH_3CN 0.1 % TFA in H_2O 0.1% TFA, 10 min, C18).

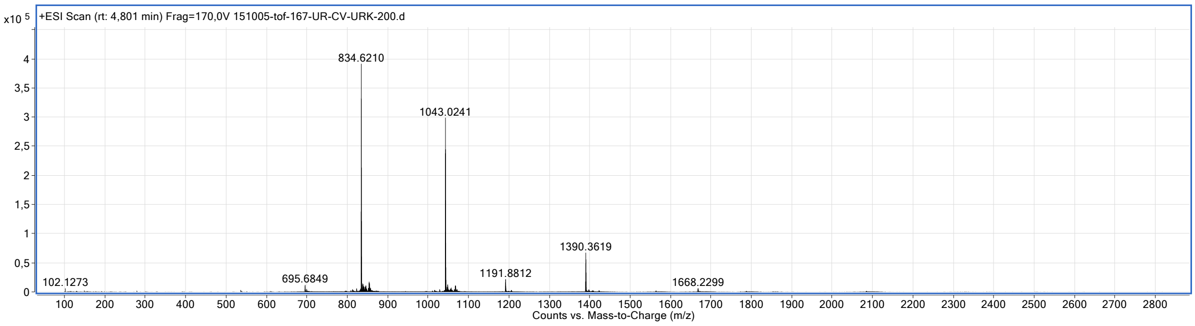

Figure S59 : LC-MS spectrum of peptide **10** performed on UHPLC coupled to an ESI -MS Tof with the dual-ESI source operated in positive ion mode. (Gradient: 10-100%; CH_3CN 0.1 % Formic acid in H_2O 0.1% formic acid, 6 min, C18).

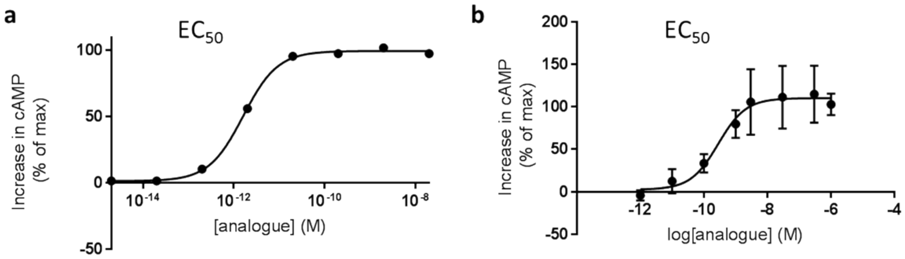

Figure S60 : Concentration-response curve (receptor-mediated cAMP produced) for EC_{50} determination of **10** in cells expressing the GLP-1R (a) Method C1 (b) Method C2 (c) Concentration-inhibition curve of **10** in a radioligand ([^{125}I]GLP-1(7-36)) binding assay on cell membrane homogenates for affinity (IC_{50}) to GLP-1R determination.

H-HA^{u}EGTFTSDLSKQLEEEAVKLFIEWLKNGGPSSGAPPPS-NH$_2$ (11, URK-434)

Oligomer **11** has been synthesized using the general procedure **P1** starting from sieber resin (160 mg, 0.1 mmol). The final product **11** was purified by semi-preparative HPLC. 2.9 mg was obtained (yield 0.7 %). **HPLC**: R$_t$= 5.28 min (10-100% CH_3CN 0.1 % TFA in H_2O 0.1% TFA, 10 min, C18); **LC-MS** *(m/z 4211.67)*: 843.24 $[M+4H]^{4+}$, 1053.80 $[M+3H]^{3+}$, 1404.73 $[M+2H]^{2+}$

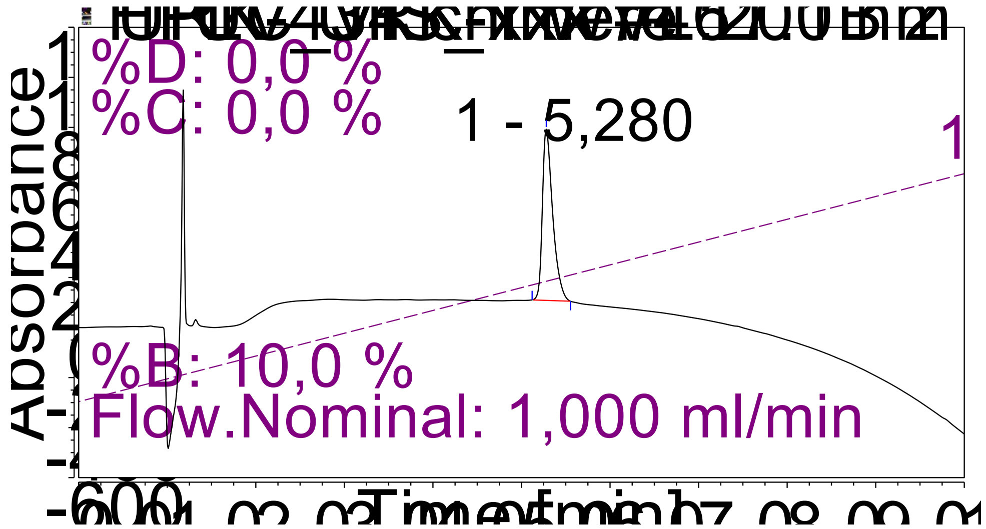

Figure S61 : HPLC profile of oligomer **11** (Gradient: 10-100%; CH_3CN 0.1 % TFA in H_2O 0.1% TFA, 10 min, C18).

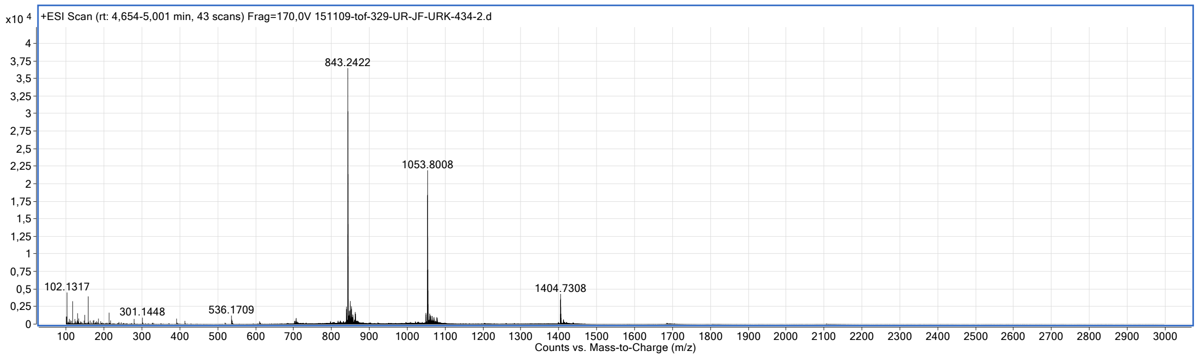

Figure S62 : LC-MS spectrum of oligomer **11** performed on UHPLC coupled to an ESI -MS Tof with the dual-ESI source operated in positive ion mode. (Gradient: 10-100%; CH_3CN 0.1 % Formic acid in H_2O 0.1% formic acid, 6 min, C18).

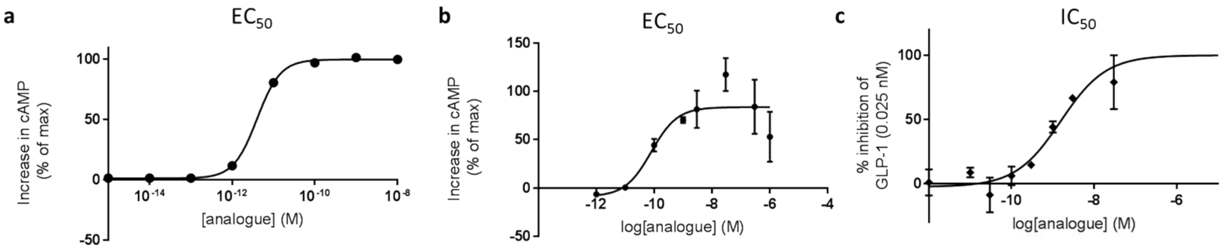

Figure S63 : Concentration-response curve (receptor-mediated cAMP produced) for EC$_{50}$ determination of **11** in cells expressing the GLP-1R (a) Method C1 (b) Method C2. (c) Concentration-inhibition curve of **11** in a radioligand ([^{125}I]GLP-1(7-36)) binding assay on cell membrane homogenates for affinity (IC$_{50}$) to GLP-1R determination.

H-HAEGTFTSDLSKQMEEEAVKLFIEWLKNGGPSSGAPPSKKKKKK-NH$_2$ (12, URK-782)

Peptide **12** has been synthesized using the general procedure **P1** starting from sieber resin (160 mg, 0.1 mmol). The final product **12** was purified by semi-preparative HPLC. 1.1 mg was obtained (yield 0.62%). **HPLC**: R_t= 4.90 min (10-100% CH_3CN 0.1 % TFA in H_2O 0.1% TFA, 10 min, C18); **ESI-MS** *(m/z 4858.56)*: 540.84 $[M+9H]^{9+}$, 608.31 $[M+8H]^{8+}$, 695.06 $[M+7H]^{7+}$, 810.73 $[M+6H]^{6+}$, 972.66 $[M+5H]^{5+}$

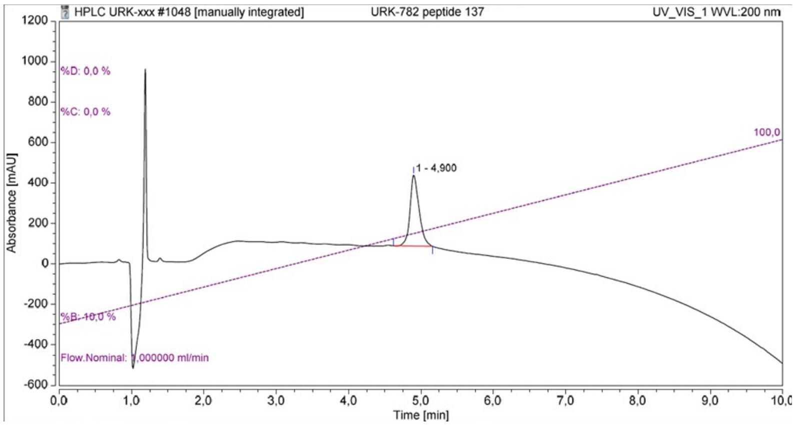

Figure S64 : HPLC profile of oligomer **12** (Gradient: 10-100%; CH_3CN 0.1 % TFA in H_2O 0.1% TFA, 10 min, C18).

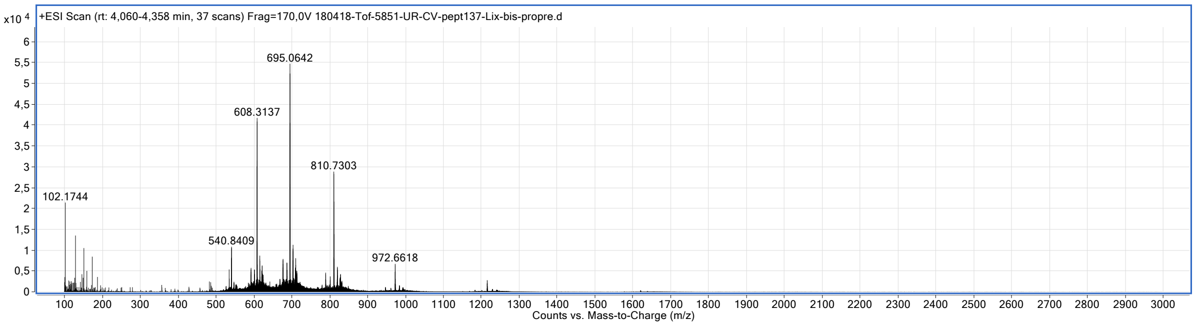

Figure S65 : LC-MS spectrum of oligomer **12** performed on UHPLC coupled to an ESI -MS Tof with the dual-ESI source operated in positive ion mode. (Gradient: 10-100%; CH_3CN 0.1 % Formic acid in H_2O 0.1% formic acid, 6 min, C18).

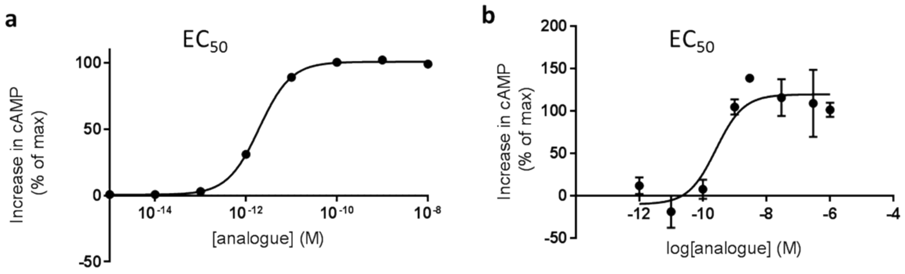

Figure S66 : Concentration-response curve (receptor-mediated cAMP produced) for EC_{50} determination of **12** in cells expressing the GLP-1R (a) Method C1 (b) Method C2.

H-HAEGTFTSDLSKQLEEEAVKLFIEWLKNGGPSSGAPPSKKKKKK-NH_2 (13, URK-479)

Peptide **13** has been synthesized using the general procedure **P1** starting from sieber resin (160 mg, 0.1 mmol). The final product **13** was purified by semi-preparative HPLC. 2.97 mg was obtained (yield 0.6 %). **HPLC**: R_t= 5.04 min (10-100% CH_3CN 0.1 % TFA in H_2O 0.1% TFA, 10 min, C18); **ESI-MS** *(m/z 4840.53)*: 538.68 $[M+9H]^{9+}$, 605.89 $[M+8H]^{8+}$, 692.31 $[M+7H]^{7+}$, 807.53 $[M+6H]^{6+}$, 969.04 $[M+5H]^{5+}$1210.81 $[M+4H]^{4+}$

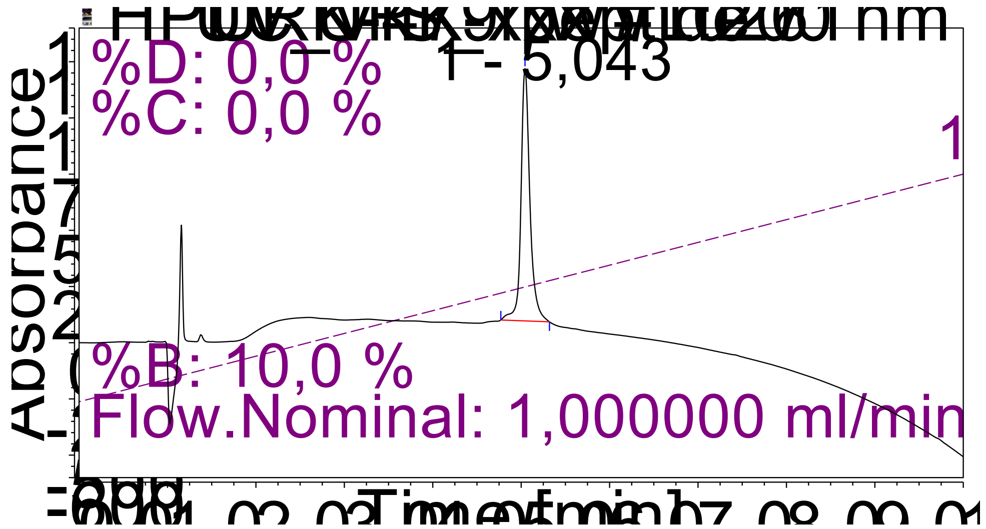

Figure S67 : HPLC profile of oligomer **13** (Gradient: 10-100%; CH_3CN 0.1 % TFA in H_2O 0.1% TFA, 10 min, C18).

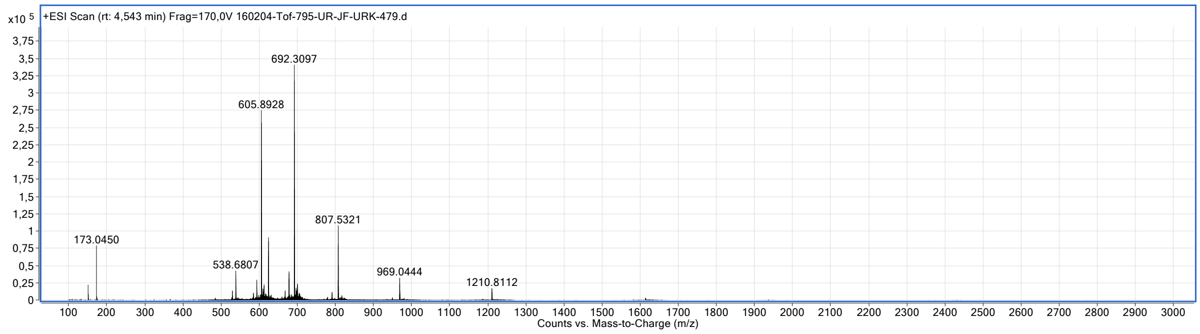

Figure S68 : LC-MS spectrum of oligomer **13** performed on UHPLC coupled to an ESI -MS Tof with the dual-ESI source operated in positive ion mode. (Gradient: 10-100%; CH_3CN 0.1 % Formic acid in H_2O 0.1% formic acid, 6 min, C18).

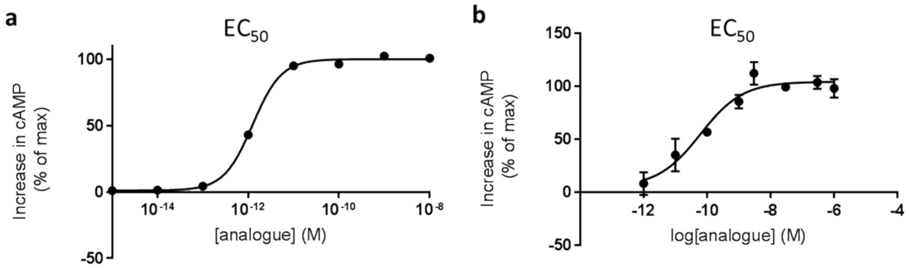

Figure S69 : Concentration-response curve (receptor-mediated cAMP produced) for EC_{50} determination of **13** in cells expressing the GLP-1R (a) Method C1 (b) Method C2.

H-HA^{u}EGTFTSDLSKQLEEEAVKLFIEWLKNGGPSSGAPPSKKKKKK-NH$_2$ (14, URK-468)

Oligomer **14** has been synthesized using the general procedure **P1** starting from sieber resin (160 mg, 0.1 mmol). The final product **14** was purified by semi-preparative HPLC. 3.4 mg was obtained (yield 0.7 %). **HPLC**: R_t= 5.06 min (10-100% CH_3CN 0.1 % TFA in H_2O 0.1% TFA, 10 min, C18); **LC-MS** *(m/z 4883.6)*: 611.45 $[M+8H]^{8+}$, 698.65 $[M+7H]^{7+}$, 814.91 $[M+6H]^{6+}$, 977.88 $[M+5H]^{5+}$

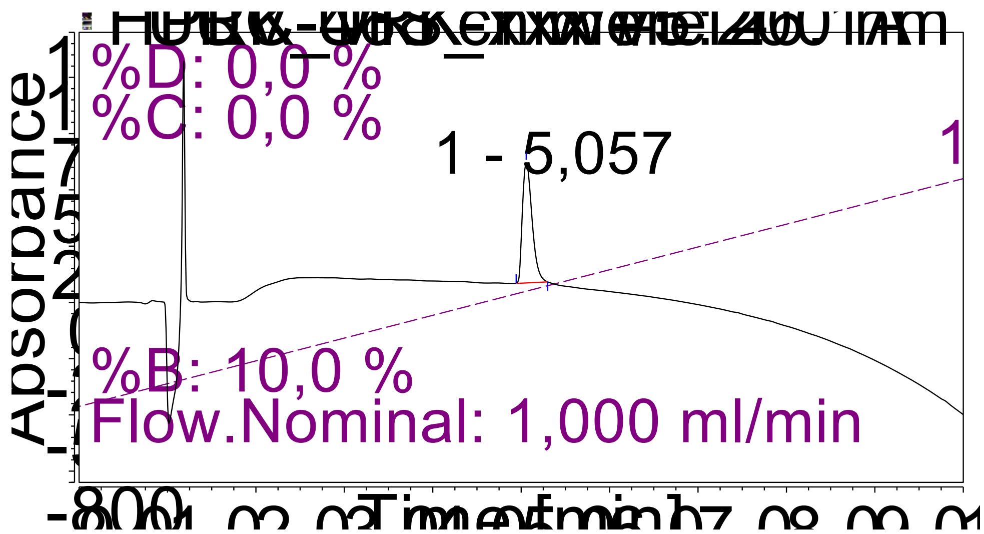

Figure S70 : HPLC profile of oligomer **14** (Gradient: 10-100%; CH_3CN 0.1 % TFA in H_2O 0.1% TFA, 10 min, C18).

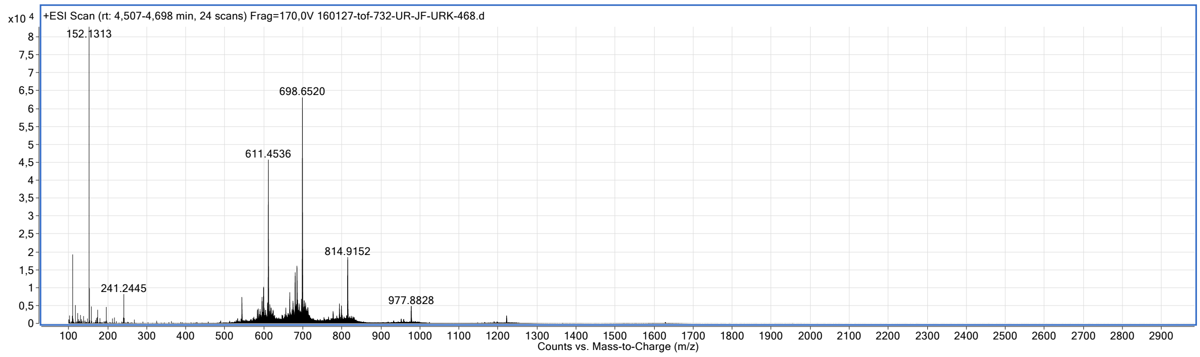

Figure S71 : LC-MS spectrum of oligomer **14** performed on UHPLC coupled to an ESI -MS Tof with the dual-ESI source operated in positive ion mode. (Gradient: 10-100%; CH_3CN 0.1 % Formic acid in H_2O 0.1% formic acid, 6 min, C18).

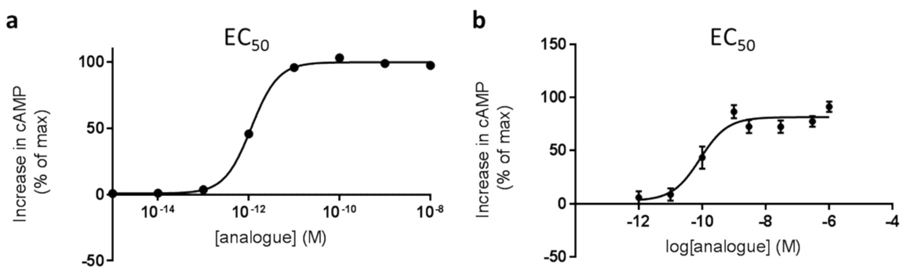

Figure S72 : Concentration-response curve (receptor-mediated cAMP produced) for EC_{50} determination of **14** in cells expressing the GLP-1R (a) Method C1 (b) Method C2.

H- HAEGTFTSDVSSYLEGQAAK*EFIAWLVRGRG-NH_2 (15, URK-781)

Oligomer **15** has been synthesized using the general procedure **P2** starting from sieber resin (160 mg, 0.1 mmol). The final product **15** was purified by semi-preparative HPLC. 0.77 mg was obtained (yield 0.2 %). **HPLC**: R_t= 6.70 min (10-100% CH_3CN 0.1 % TFA in H_2O 0.1% TFA, 10 min, C18); **LC-MS** *(m/z 3751.26)*: 938.82 $[M+4H]^{4+}$, 1251.41 $[M+3H]^{3+}$, 1876.58 $[M+2H]^{2+}$

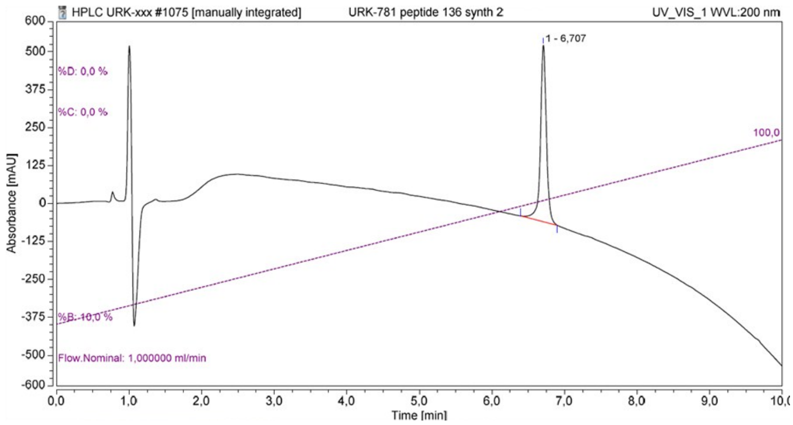

Figure S73 : HPLC profile of oligomer **15** (Gradient: 10-100%; CH_3CN 0.1 % TFA in H_2O 0.1% TFA, 10 min, C18).

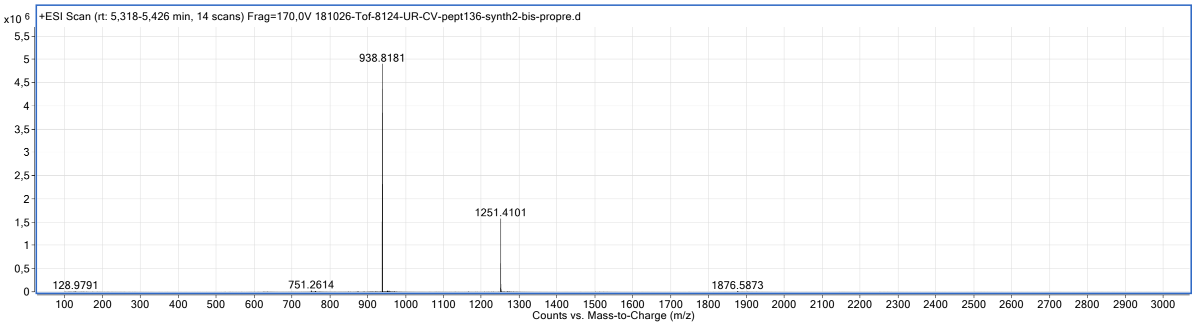

Figure S74 : LC-MS spectrum of oligomer **15** performed on UHPLC coupled to an ESI -MS Tof with the dual-ESI source operated in positive ion mode. (Gradient: 10-100%; CH_3CN 0.1 % Formic acid in H_2O 0.1% formic acid, 6 min, C18).

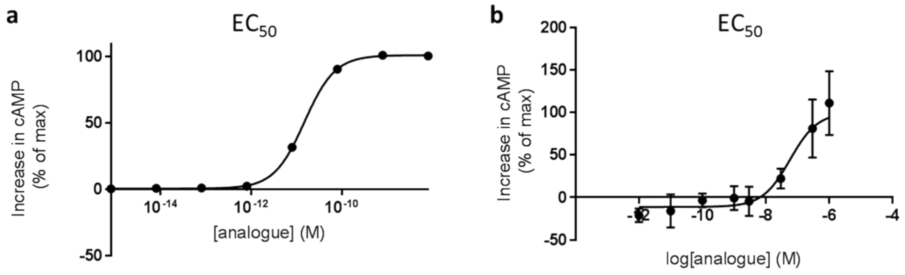

Figure S75 : Concentration-response curve (receptor-mediated cAMP produced) for EC_{50} determination of **15** in cells expressing the GLP-1R (a) Method C1 (b) Method C2.

H-HS^{u}EGTFTSDVSSYLEGQAAKEFIAWLVKGRG-NH$_2$ (S1, URK-524)

Oligomer **S1** has been synthesized using the general procedure **P1** starting from sieber resin (160 mg, 0.1 mmol). The final product **S1** was purified by semi-preparative HPLC. 1.8 mg was obtained (yield 0.5 %). **HPLC**: R_t= 5.17 min (10-100% CH_3CN 0.1 % TFA in H_2O 0.1% TFA, 10 min, C18); **LC-MS** *(m/z 3397.77)*: 674.94 $[M+5H]^{5+}$, 843.42 $[M+4H]^{4+}$, 1123.89 $[M+3H]^{3+}$

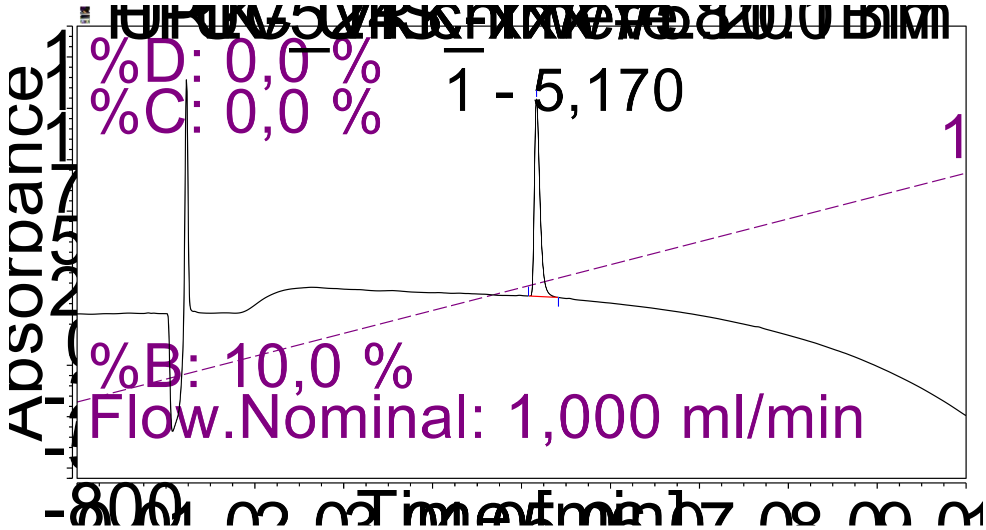

Figure S76 : HPLC profile of oligomer **S1** (Gradient: 10-100%; CH_3CN 0.1 % TFA in H_2O 0.1% TFA, 10 min, C18).

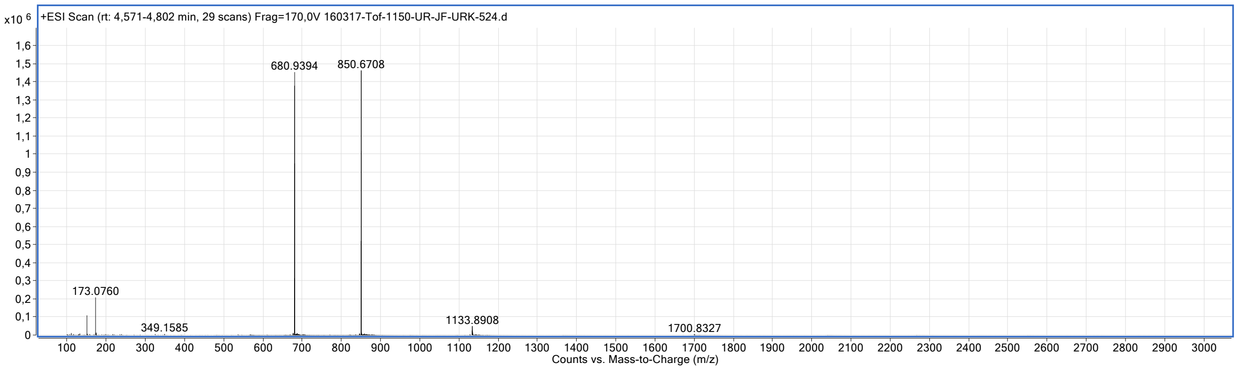

Figure S77 : LC-MS spectrum of oligomer **S1** performed on UHPLC coupled to an ESI -MS Tof with the dual-ESI source operated in positive ion mode. (Gradient: 10-100%; CH_3CN 0.1 % Formic acid in H_2O 0.1% formic acid, 6 min, C18).

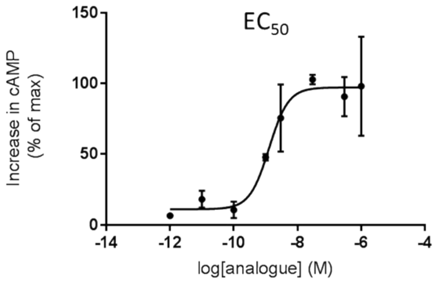

Figure S78 : Concentration-response curve (receptor-mediated cAMP produced) for EC_{50} determination of **S1** in cells expressing the GLP-1R (Method C2).

H-HR^{u}EGTFTSDVSSYLEGQAAKEFIAWLVKGRG-NH$_2$ (S2, URK-555)

Oligomer **S2** has been synthesized using the general procedure **P1** starting from sieber resin (160 mg, 0.1 mmol). The final product **S2** was purified by semi-preparative HPLC. 2.2 mg was obtained (yield 0.6 %). **HPLC**: R_t= 5.13 min (10-100% CH_3CN 0.1 % TFA in H_2O 0.1% TFA, 10 min, C18); **LC-MS** *(m/z 3468.89)*: 578.97 $[M+6H]^{6+}$, 694.76 $[M+5H]^{5+}$, 868.20 $[M+4H]^{4+}$, 1157.26 $[M+3H]^{3+}$

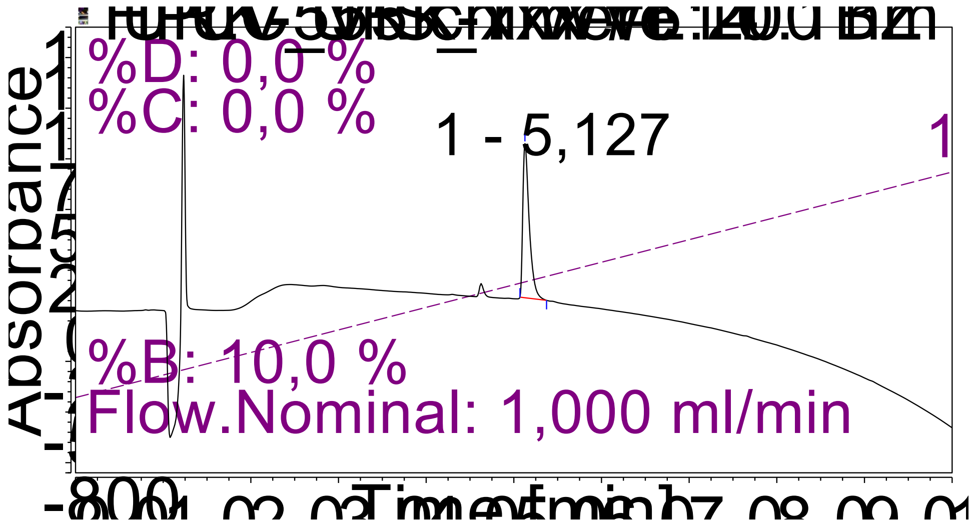

Figure S79 : HPLC profile of oligomer **S2** (Gradient: 10-100%; CH_3CN 0.1 % TFA in H_2O 0.1% TFA, 10 min, C18).

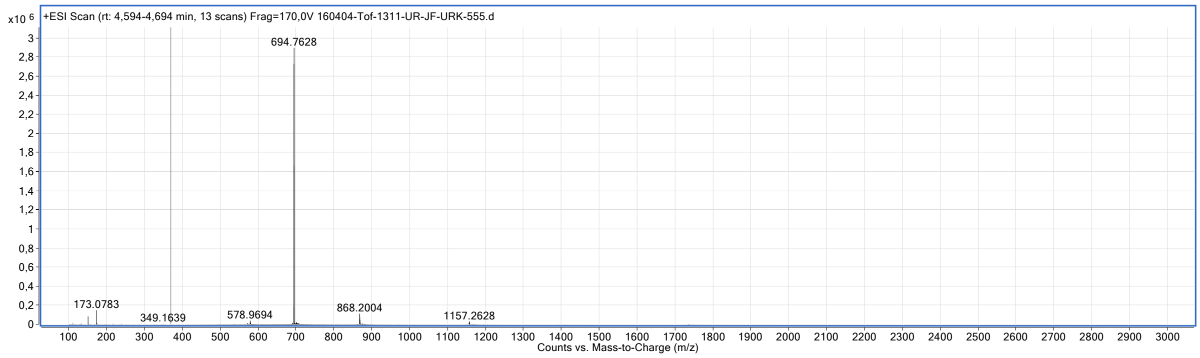

Figure S80 : LC-MS spectrum of oligomer **S2** performed on UHPLC coupled to an ESI -MS Tof with the dual-ESI source operated in positive ion mode. (Gradient: 10-100%; CH_3CN 0.1 % Formic acid in H_2O 0.1% formic acid, 6 min, C18).

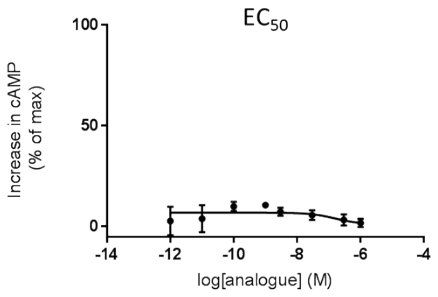

Figure S81 : Concentration-response curve (receptor-mediated cAMP produced) for EC_{50} determination of **S2** in cells expressing the GLP-1R (Method C2).

H-HC[u]EGTFTSDVSSYLEGQAAKEFIAWLVKGRG-NH$_2$ (S3, URK-642)

Oligomer **S3** has been synthesized using the general procedure **P1** starting from sieber resin (160 mg, 0.1 mmol). The final product **S3** was purified by semi-preparative HPLC. 3.3 mg was obtained (yield 0.96 %). **HPLC**: R_t= 5.19 min (10-100% CH_3CN 0.1 % TFA in H_2O 0.1% TFA, 10 min, C18); **LC-MS** *(m/z 3415.84)*: 684.04 $[M+5H]^{5+}$, 854.81 $[M+4H]^{4+}$, 1139.43 $[M+3H]^{3+}$, 1708.69 $[M+2H]^{2+}$

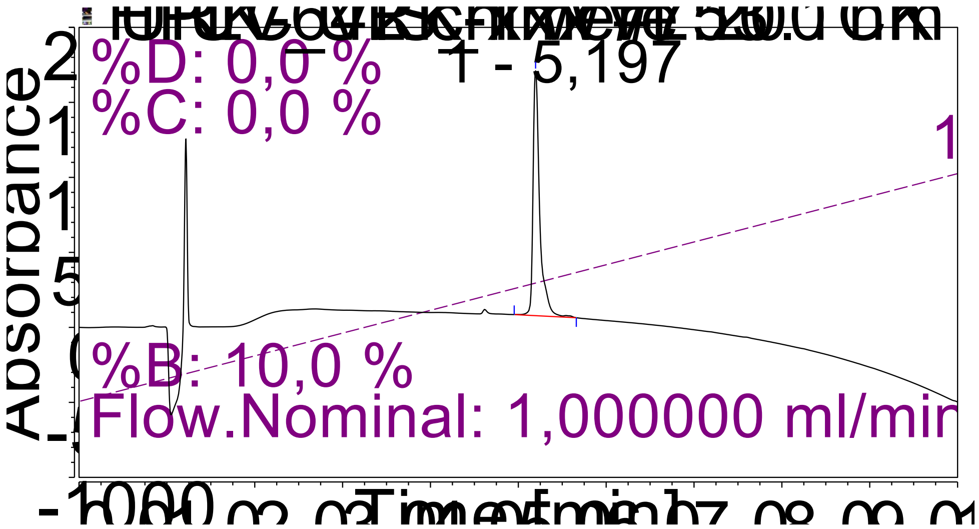

Figure S82 : HPLC profile of oligomer **S3** (Gradient: 10-100%; CH_3CN 0.1 % TFA in H_2O 0.1% TFA, 10 min, C18).

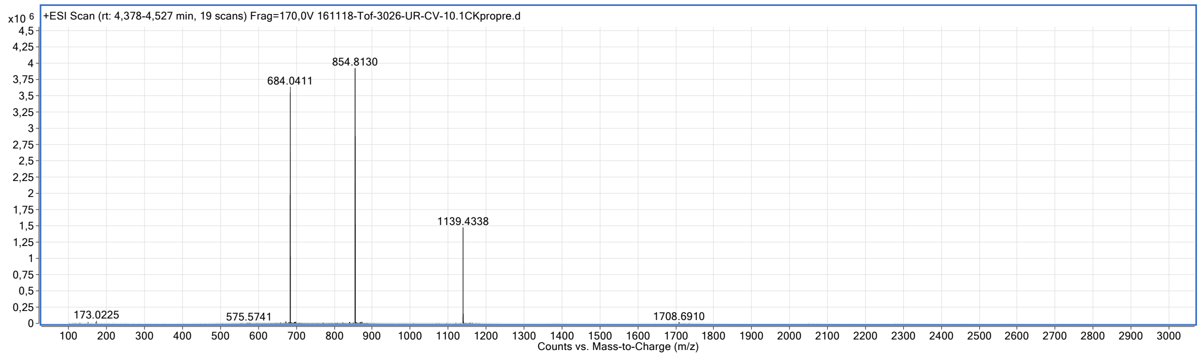

Figure S83 : LC-MS spectrum of oligomer **S3** performed on UHPLC coupled to an ESI -MS Tof with the dual-ESI source operated in positive ion mode. (Gradient: 10-100%; CH_3CN 0.1 % Formic acid in H_2O 0.1% formic acid, 6 min, C18).

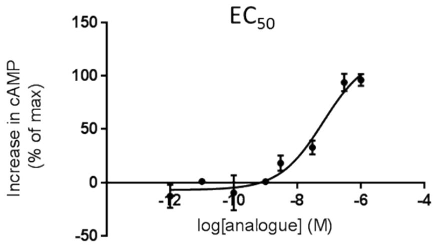

Figure S84 : Concentration-response curve (receptor-mediated cAMP produced) for EC_{50} determination of **S3** in cells expressing the GLP-1R (Method C2).

H-HQ^{u}EGTFTSDVSSYLEGQAAKEFIAWLVKGRG-NH_2 (S4, URK-548)

Oligomer **S4** has been synthesized using the general procedure **P1** starting from sieber resin (160 mg, 0.1 mmol). The final product **S4** was purified by semi-preparative HPLC. 2.8 mg was obtained (yield 0.8 %). **HPLC**: R_t= 5.16 min (10-100% CH_3CN 0.1 % TFA in H_2O 0.1% TFA, 10 min, C18); **LC-MS** *(m/z 3440.83)*: 688.95 $[M+5H]^{5+}$, 860.94 $[M+4H]^{4+}$, 1147.91 $[M+3H]^{3+}$, 1721.36 $[M+2H]^{2+}$

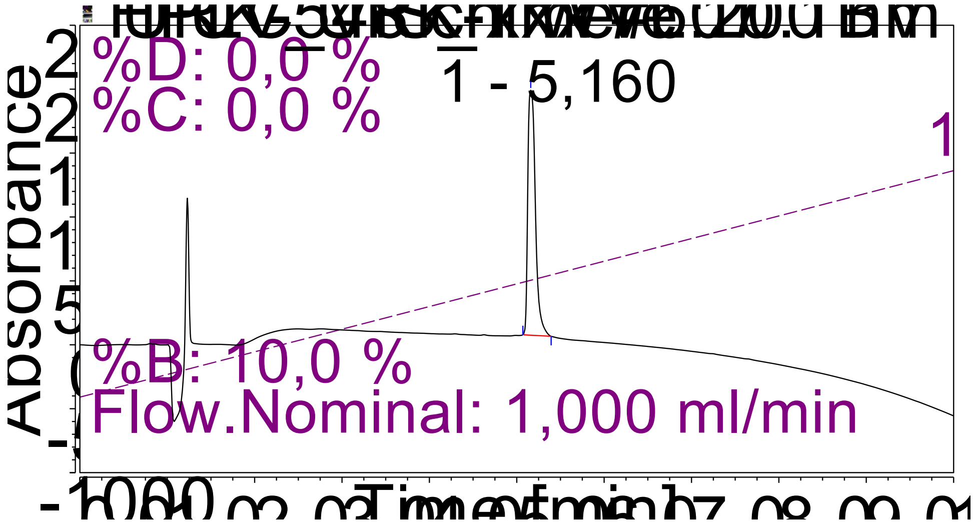

Figure S85 : HPLC profile of oligomer **S4** (Gradient: 10-100%; CH_3CN 0.1 % TFA in H_2O 0.1% TFA, 10 min, C18).

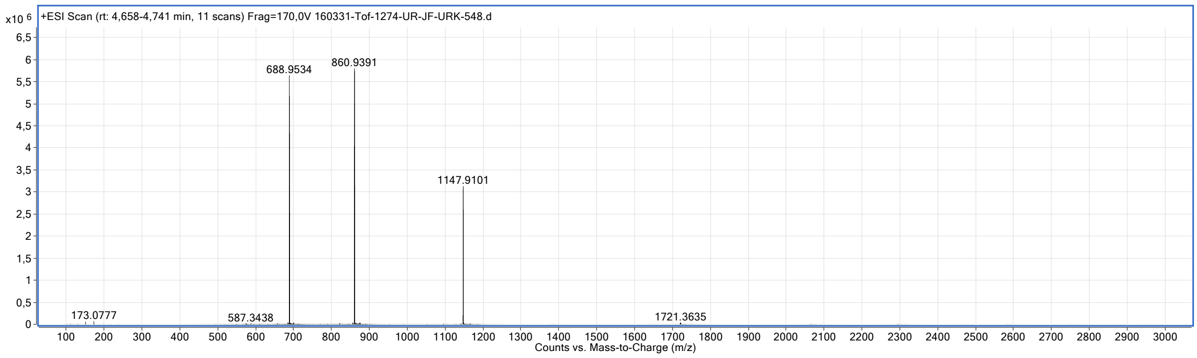

Figure S86 : LC-MS spectrum of oligomer **S4** performed on UHPLC coupled to an ESI -MS Tof with the dual-ESI source operated in positive ion mode. (Gradient: 10-100%; CH_3CN 0.1 % Formic acid in H_2O 0.1% formic acid, 6 min, C18).

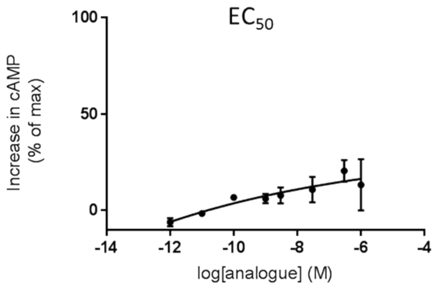

Figure S87 : Concentration-response curve (receptor-mediated cAMP produced) for EC_{50} determination of **S4** in cells expressing the GLP-1R (Method C2)**.**

H-HE^uEGTFTSDVSSYLEGQAAKEFIAWLVKGRG-NH$_2$ (S5, URK-544)

Oligomer **S5** has been synthesized using the general procedure **P1** starting from sieber resin (160 mg, 0.1 mmol). The final product **S5** was purified by semi-preparative HPLC. 2.8 mg was obtained (yield 0.8 %). **HPLC**: R_t= 5.16 min (10-100% CH_3CN 0.1 % TFA in H_2O 0.1% TFA, 10 min, C18); **LC-MS** *(m/z 3441.81)*: 689.15 $[M+5H]^{5+}$, 861.43 $[M+4H]^{4+}$, 1148.24 $[M+3H]^{3+}$, 1721.85 $[M+2H]^{2+}$

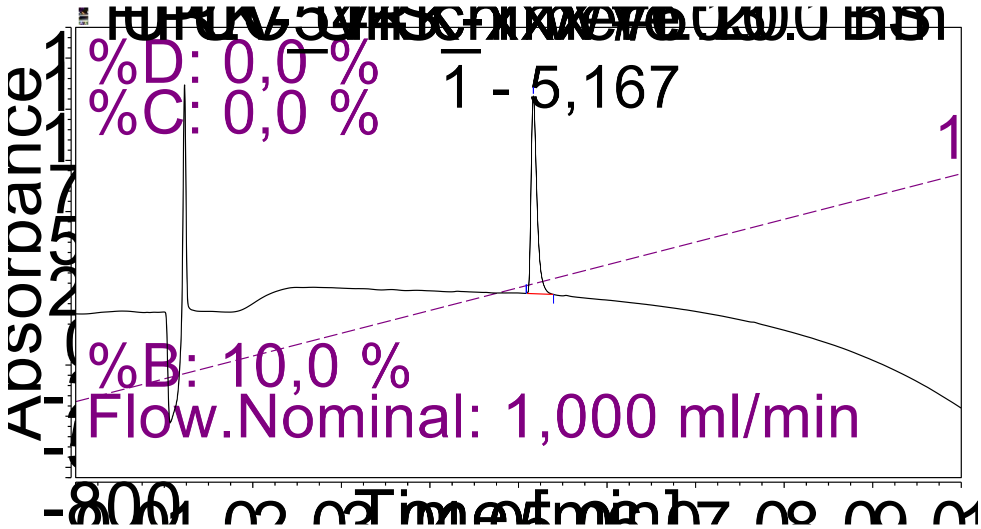

Figure S88 : HPLC profile of oligomer **S5** (Gradient: 10-100%; CH_3CN 0.1 % TFA in H_2O 0.1% TFA, 10 min, C18).

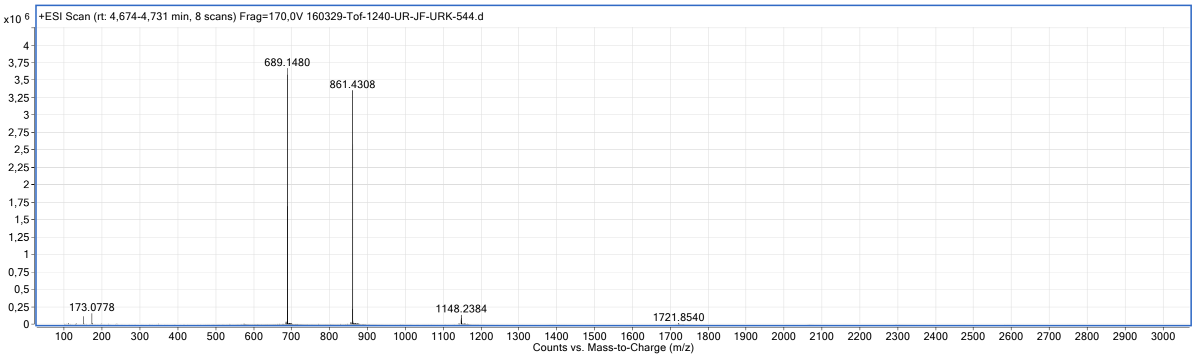

Figure S89 : LC-MS spectrum of oligomer **S5** performed on UHPLC coupled to an ESI -MS Tof with the dual-ESI source operated in positive ion mode. (Gradient: 10-100%; CH_3CN 0.1 % Formic acid in H_2O 0.1% formic acid, 6 min, C18).

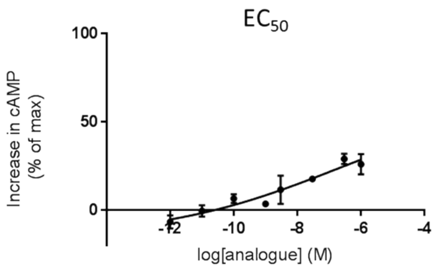

Figure S90 : Concentration-response curve (receptor-mediated cAMP produced) for EC_{50} determination of **S5** in cells expressing the GLP-1R (Method C2).

H-HI^{u}EGTFTSDVSSYLEGQAAKEFIAWLVKGRG-NH$_2$ (S6, URK-562)

Oligomer **S6** has been synthesized using the general procedure **P1** starting from sieber resin (160 mg, 0.1 mmol). The final product **S6** was purified by semi-preparative HPLC. 2.2 mg was obtained (yield 0.6 %). **HPLC**: R_t= 5.27 min (10-100% CH_3CN 0.1 % TFA in H_2O 0.1% TFA, 10 min, C18); **LC-MS** *(m/z 3425.86)*: 685.96 $[M+5H]^{5+}$, 857.19 $[M+4H]^{4+}$, 1142.92 $[M+3H]^{3+}$, 1713.88 $[M+2H]^{2+}$

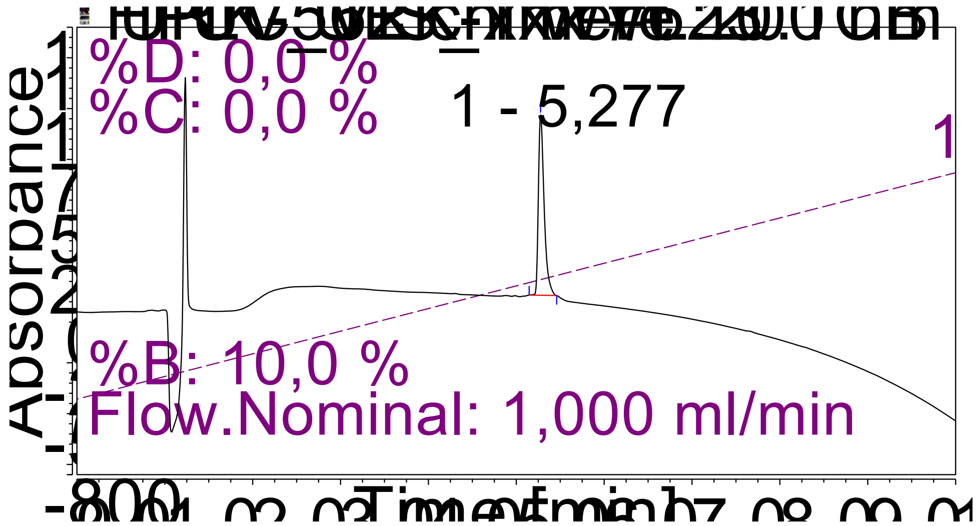

Figure S91 : HPLC profile of oligomer **S6** (Gradient: 10-100%; CH_3CN 0.1 % TFA in H_2O 0.1% TFA, 10 min, C18).

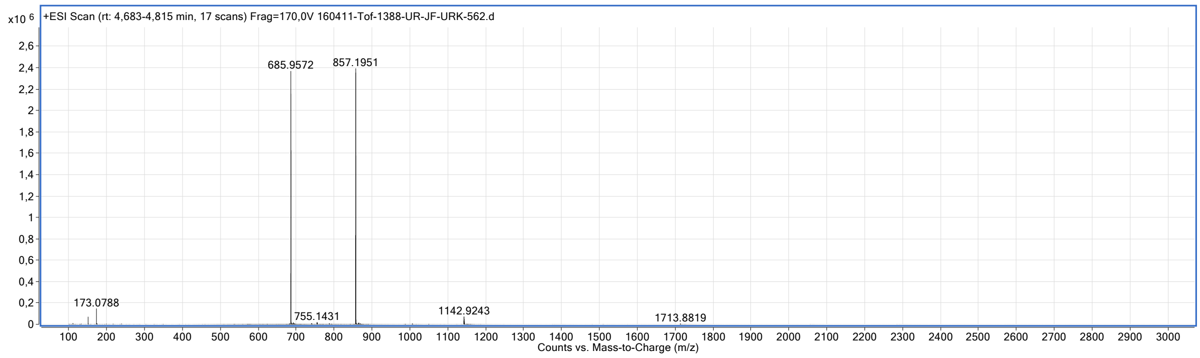

Figure S92 : LC-MS spectrum of oligomer **S6** performed on UHPLC coupled to an ESI -MS Tof with the dual-ESI source operated in positive ion mode. (Gradient: 10-100%; CH_3CN 0.1 % Formic acid in H_2O 0.1% formic acid, 6 min, C18).

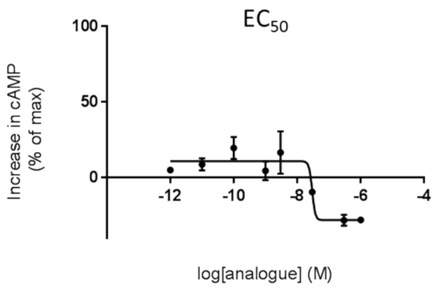

Figure S93 : Concentration-response curve (receptor-mediated cAMP produced) for EC_{50} determination of **S6** in cells expressing the GLP-1R (Method C2).

H-HL[u]EGTFTSDVSSYLEGQAAKEFIAWLVKGRG-NH$_2$ (S7, URK-551)

Oligomer **S7** has been synthesized using the general procedure **P1** starting from sieber resin (160 mg, 0.1 mmol). The final product **S7** was purified by semi-preparative HPLC. 2.1 mg was obtained (yield 0.6 %). **HPLC**: R_t= 5.29 min (10-100% CH_3CN 0.1 % TFA in H_2O 0.1% TFA, 10 min, C18); **LC-MS** *(m/z 3425.86)*: 686.15 $[M+5H]^{5+}$, 857.44 $[M+4H]^{4+}$, 1142.92 $[M+3H]^{3+}$

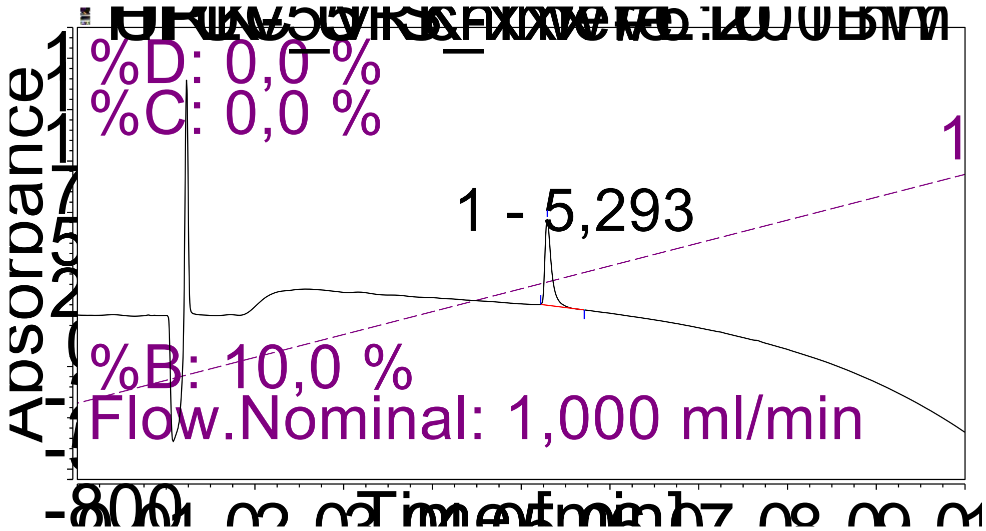

Figure S94 : HPLC profile of oligomer **S7** (Gradient: 10-100%; CH_3CN 0.1 % TFA in H_2O 0.1% TFA, 10 min, C18).

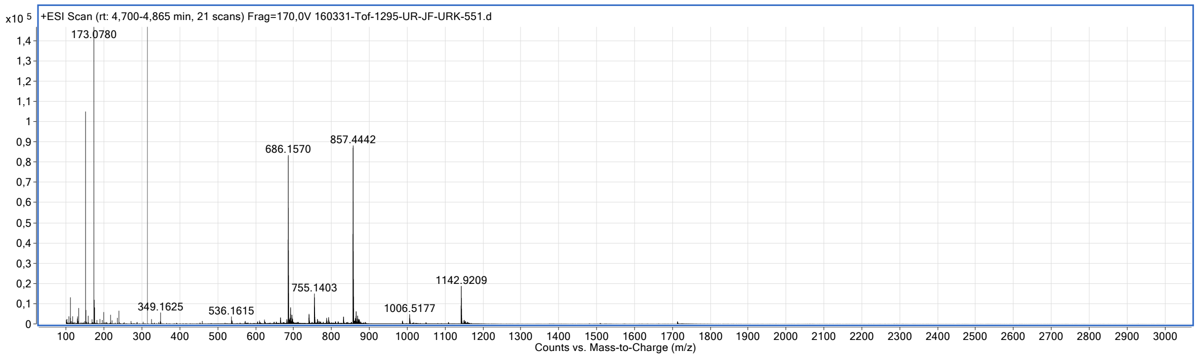

Figure S95 : LC-MS spectrum of oligomer **S7** performed on UHPLC coupled to an ESI -MS Tof with the dual-ESI source operated in positive ion mode. (Gradient: 10-100%; CH_3CN 0.1 % Formic acid in H_2O 0.1% formic acid, 6 min, C18).

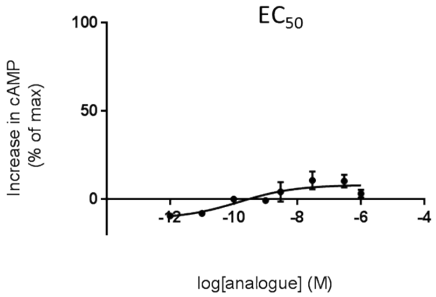

Figure S96 : Concentration-response curve (receptor-mediated cAMP produced) for EC_{50} determination of **S7** in cells expressing the GLP-1R (Method C2).

H-HK^{u}EGTFTSDVSSYLEGQAAKEFIAWLVKGRG-NH$_2$ (S8, URK-545)

Oligomer **S8** has been synthesized using the general procedure **P1** starting from sieber resin (160 mg, 0.1 mmol). The final product **S8** was purified by semi-preparative HPLC. 3.1 mg was obtained (yield 0.9 %). **HPLC**: R_t= 5.08 min (10-100% CH_3CN 0.1 % TFA in H_2O 0.1% TFA, 10 min, C18); **LC-MS** *(m/z 3440.87)*: 574.30 $[M+6H]^{6+}$, 686.16 $[M+5H]^{5+}$, 860.94 $[M+4H]^{4+}$, 1147.92 $[M+3H]^{3+}$

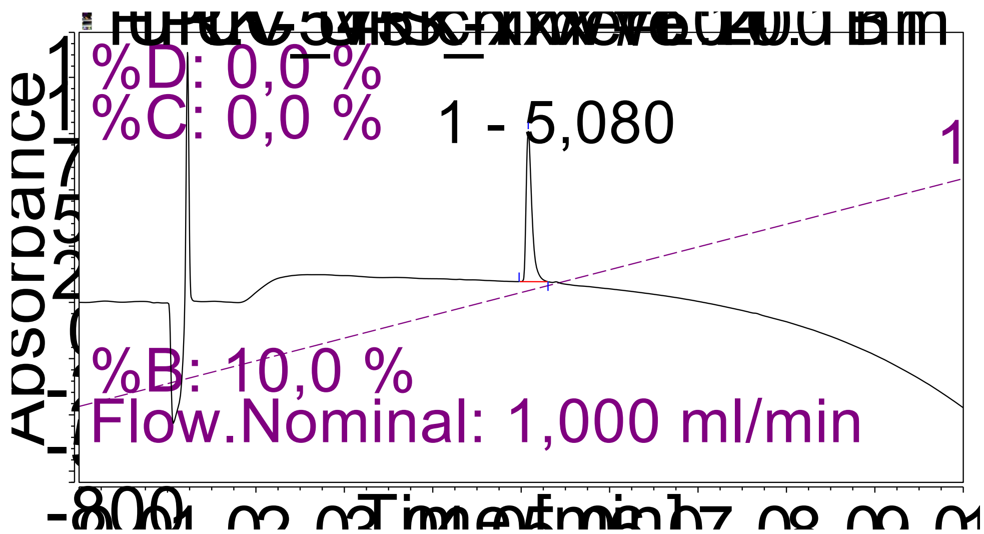

Figure S97 : HPLC profile of oligomer **S8** (Gradient: 10-100%; CH_3CN 0.1 % TFA in H_2O 0.1% TFA, 10 min, C18).

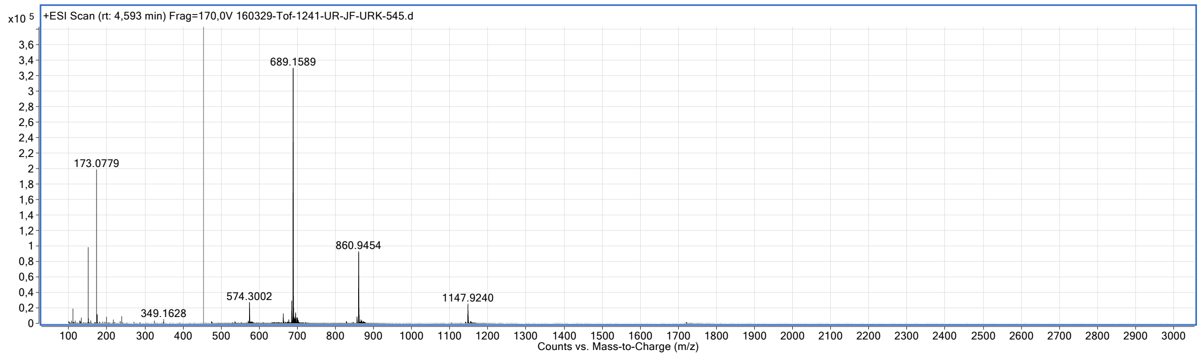

Figure S98 : LC-MS spectrum of oligomer **S8** performed on UHPLC coupled to an ESI -MS Tof with the dual-ESI source operated in positive ion mode. (Gradient: 10-100%; CH_3CN 0.1 % Formic acid in H_2O 0.1% formic acid, 6 min, C18).

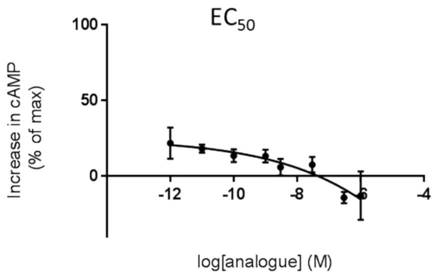

Figure S99 : Concentration-response curve (receptor-mediated cAMP produced) for EC_{50} determination of **S8** in cells expressing the GLP-1R (Method C2).

H-HF^{u}EGTFTSDVSSYLEGQAAKEFIAWLVKGRG-NH$_2$ (S9, URK-546)

Oligomer **S9** has been synthesized using the general procedure **P1** starting from sieber resin (160 mg, 0.1 mmol). The final product **S9** was purified by semi-preparative HPLC. 2.6 mg was obtained (yield 0.8 %). **HPLC**: R_t= 5.08 min (10-100% CH_3CN 0.1 % TFA in H_2O 0.1% TFA, 10 min, C18); **LC-MS** *(m/z 3459.87)*: 577.63 $[M+6H]^{6+}$, 692.75 $[M+5H]^{5+}$, 865.68 $[M+4H]^{4+}$, 1154.25 $[M+3H]^{3+}$, 1730.87 $[M+2H]^{2+}$

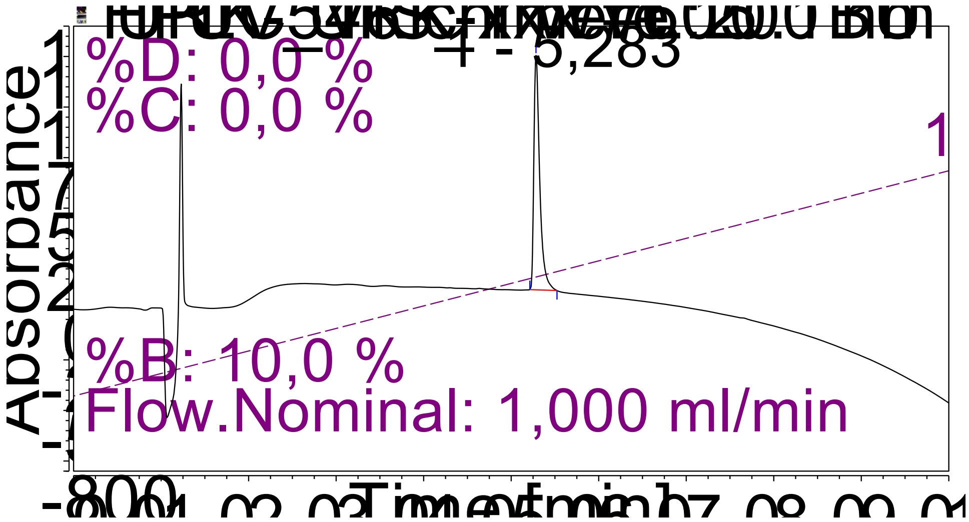

Figure S100 : HPLC profile of oligomer **S9** (Gradient: 10-100%; CH_3CN 0.1 % TFA in H_2O 0.1% TFA, 10 min, C18).

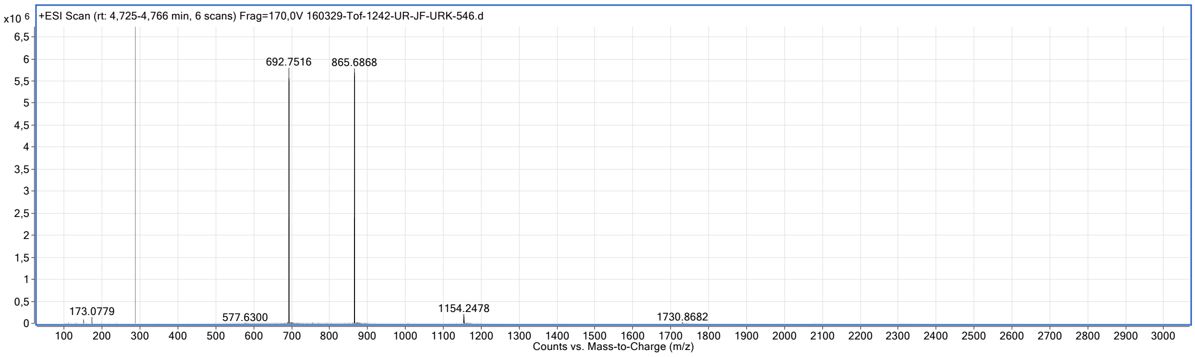

Figure S101 : LC-MS spectrum of oligomer **S9** performed on UHPLC coupled to an ESI -MS Tof with the dual-ESI source operated in positive ion mode. (Gradient: 10-100%; CH_3CN 0.1 % Formic acid in H_2O 0.1% formic acid, 6 min, C18).

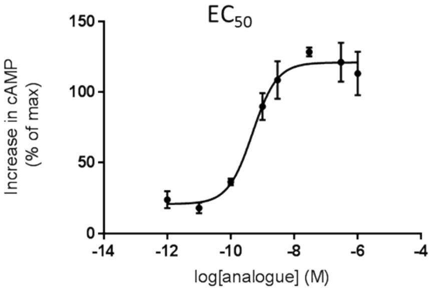

Figure S102 : Concentration-response curve (receptor-mediated cAMP produced) for EC_{50} determination of **9** in cells expressing the GLP-1R (Method C2).

H-HT^{u}EGTFTSDVSSYLEGQAAKEFIAWLVKGRG-NH$_2$ (S10, URK-563)

Oligomer **S10** has been synthesized using the general procedure **P1** starting from sieber resin (160 mg, 0.1 mmol). The final product **S10** was purified by semi-preparative HPLC. 2.1 mg was obtained (yield 0.6 %). **HPLC**: R_t= 5.18 min (10-100% CH_3CN 0.1 % TFA in H_2O 0.1% TFA, 10 min, C18); **LC-MS** *(m/z 3413.8)*: 569.79 $[M+6H]^{6+}$, 683.75 $[M+5H]^{5+}$, 854.19 $[M+4H]^{4+}$, 1138.57 $[M+3H]^{3+}$, 1707.86 $[M+2H]^{2+}$

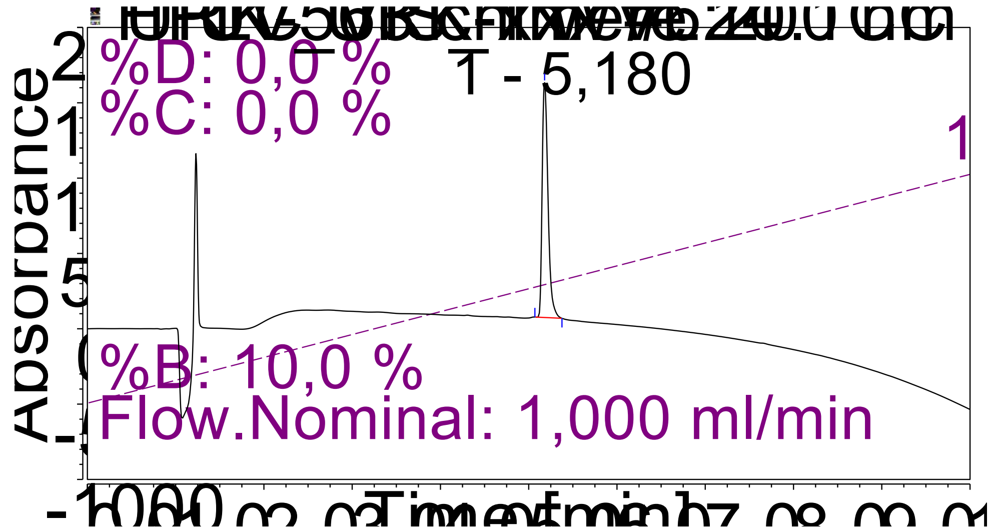

Figure S103 : HPLC profile of oligomer **S10** (Gradient: 10-100%; CH_3CN 0.1 % TFA in H_2O 0.1% TFA, 10 min, C18).

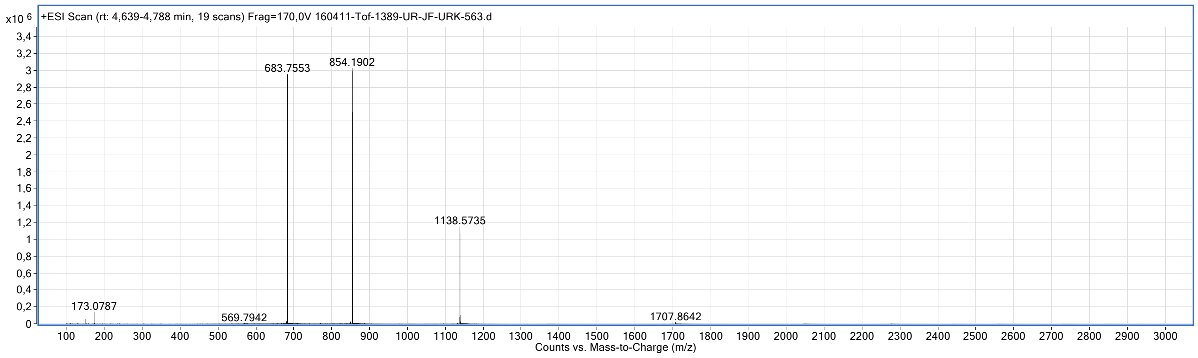

Figure S104 : LC-MS spectrum of oligomer **S10** performed on UHPLC coupled to an ESI -MS Tof with the dual-ESI source operated in positive ion mode. (Gradient: 10-100%; CH_3CN 0.1 % Formic acid in H_2O 0.1% formic acid, 6 min, C18).

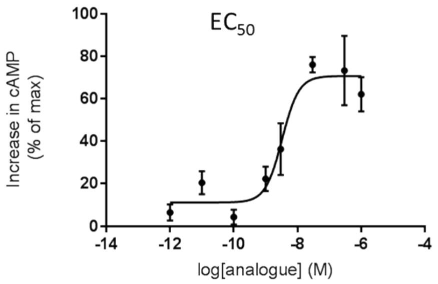

Figure S105 : Concentration-response curve (receptor-mediated cAMP produced) for EC_{50} determination of **S10** in cells expressing the GLP-1R (Method C2).

H-HW^{u}EGTFTSDVSSYLEGQAAKEFIAWLVKGRG-NH$_2$ (S11, URK-554)

Oligomer **S11** has been synthesized using the general procedure **P1** starting from sieber resin (160 mg, 0.1 mmol). The final product **S11** was purified by semi-preparative HPLC. 2.2 mg was obtained (yield 0.6 %). **HPLC**: R_t= 5.34 min (10-100% CH_3CN 0.1 % TFA in H_2O 0.1% TFA, 10 min, C18); **LC-MS** *(m/z 3498.91)*: 700.75 $[M+5H]^{5+}$, 875.69 $[M+4H]^{4+}$, 1167.25 $[M+3H]^{3+}$

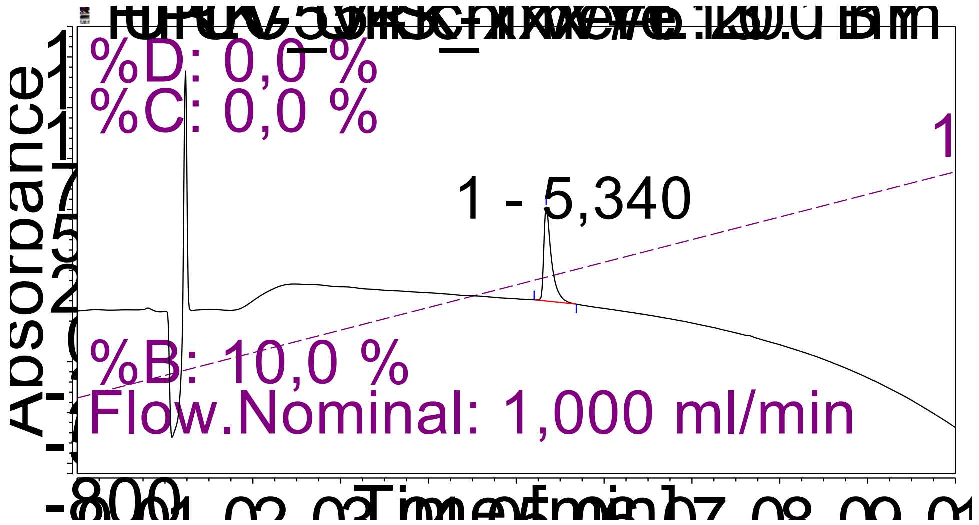

Figure S106 : HPLC profile of oligomer **S11** (Gradient: 10-100%; CH_3CN 0.1 % TFA in H_2O 0.1% TFA, 10 min, C18).

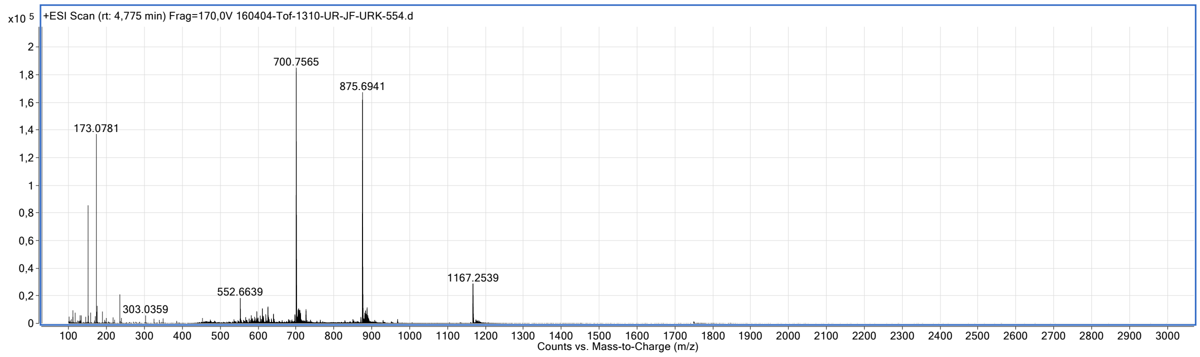

Figure S107 : LC-MS spectrum of oligomer **S11** performed on UHPLC coupled to an ESI -MS Tof with the dual-ESI source operated in positive ion mode. (Gradient: 10-100%; CH_3CN 0.1 % Formic acid in H_2O 0.1% formic acid, 6 min, C18).

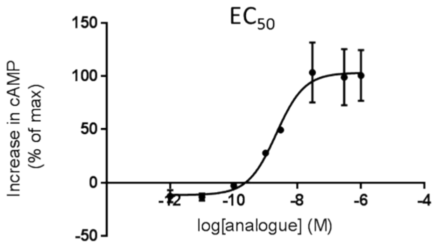

Figure S108 : Concentration-response curve (receptor-mediated cAMP produced) for EC_{50} determination of **S11** in cells expressing the GLP-1R (Method C2).

H-HGEGTFTSDVSSYLEGQAAKEFIAWLVKGRG-NH$_2$ (S12)

The synthesis of peptide **S12** has already been described [1].

References

[1] J. Fremaux, C. Venin, L. Mauran, R. H. Zimmer, G. Guichard, S. R. Goudreau, *Nat. Commun.* **2019**, *10*, DOI 10.1038/s41467-019-08793-y.

[2] J. M. Collins, K. A. Porter, S. K. Singh, G. S. Vanier, *Org. Lett.* **2014**, *16*, 940–943.

[3] S. Runge, B. S. Wulff, K. Madsen, H. Bräuner-Osborne, L. B. Knudsen, *Br. J. Pharmacol.* **2003**, *138*, 787–794.

[4] H. C. Fehmann, J. Jiang, J. Schweinfurth, M. B. Wheeler, A. E. Boyd, B. Göke, *Peptides* **1994**, *15*, 453–456.

[5] J. Naylor, A. T. Suckow, A. Seth, D. J. Baker, I. Sermadiras, P. Ravn, R. Howes, J. Li, M. R. Snaith, M. P. Coghlan, et al., *Biochem. J.* **2016**, *473*, 2881–2891.

[6] T. Kenakin, C. Watson, V. Muniz-Medina, A. Christopoulos, S. Novick, *ACS Chem. Neurosci.* **2012**, *3*, 193–203.

[7] B. Jones, T. Buenaventura, N. Kanda, P. Chabosseau, B. M. Owen, R. Scott, R. Goldin, N. Angkathunyakul, I. R. Corrêa Jr, D. Bosco, et al., *Nat. Commun.* **2018**, *9*, DOI 10.1038/s41467-018-03941-2.

[8] Y. Zhang, B. Sun, D. Feng, H. Hu, M. Chu, Q. Qu, J. T. Tarrasch, S. Li, T. Sun Kobilka, B. K. Kobilka, et al., *Nature* **2017**, *546*, 248–253.

[9] A. Violette, M. C. Averlant-Petit, V. Semetey, C. Hemmerlin, R. Casimir, R. Graff, M. Marraud, J.-P. Briand, D. Rognan, G. Guichard, *J. Am. Chem. Soc.* **2005**, *127*, 2156–2164.

[10] S. Jo, T. Kim, V. G. Iyer, W. Im, *J. Comput. Chem.* **2008**, *29*, 1859–1865.

[11] J. A. Maier, C. Martinez, K. Kasavajhala, L. Wickstrom, K. E. Hauser, C. Simmerling, *J. Chem. Theory Comput.* **2015**, *11*, 3696–3713.

[12] C. J. Dickson, B. D. Madej, Å. A. Skjevik, R. M. Betz, K. Teigen, I. R. Gould, R. C. Walker, *J. Chem. Theory Comput.* **2014**, *10*, 865–879.

[13] F. Da Silva, J. Desaphy, D. Rognan, *ChemMedChem* **2018**, *13*, 507–510.

[14] G. Marcou, D. Rognan, *J. Chem. Inf. Model.* **2007**, *47*, 195–207.

[15] G. W. Collie, K. Pulka-Ziach, G. Guichard, *Chem. Sci.* **2016**, *7*, 3377–3383.

[16] G. W. Collie, K. Pulka-Ziach, C. M. Lombardo, J. Fremaux, F. Rosu, M. Decossas, L. Mauran, O. Lambert, V. Gabelica, C. D. Mackereth, et al., *Nat. Chem.* **2015**, *7*, 871–878.

[17] N. Pendem, C. Douat, P. Claudon, M. Laguerre, S. Castano, B. Desbat, D. Cavagnat, E. Ennifar, B. Kauffmann, G. Guichard, *J. Am. Chem. Soc.* **2013**, *135*, 4884–4892.

[18] C. Douat-Casassus, K. Pulka, P. Claudon, G. Guichard, *Org. Lett.* **2012**, *14*, 3130–3133.

[19] M. Smrcina, P. Majer, E. Majerová, T. A. Guerassina, M. A. Eissenstat, *Tetrahedron* **1997**, *53*, 12867–12874.
